# CO_2_ Reduction by an Iron(I) Porphyrinate System: Effect of Hydrogen Bonding on the Second Coordination Sphere

**DOI:** 10.1021/acs.inorgchem.3c04246

**Published:** 2024-02-26

**Authors:** Chengxu Zhu, Carmine D’Agostino, Sam P. de Visser

**Affiliations:** § Manchester Institute of Biotechnology, The University of Manchester, 131 Princess Street, Manchester M1 7DN, United Kingdom; ⊥ Department of Chemical Engineering, The University of Manchester, Oxford Road, Manchester M13 9PL, United Kingdom; † Dipartimento di Ingegneria Civile, Chimica, Ambientale e dei Materiali, Alma Mater Studiorum−Università di Bologna, Via Terracini 28, 40131 Bologna, Italy

## Abstract

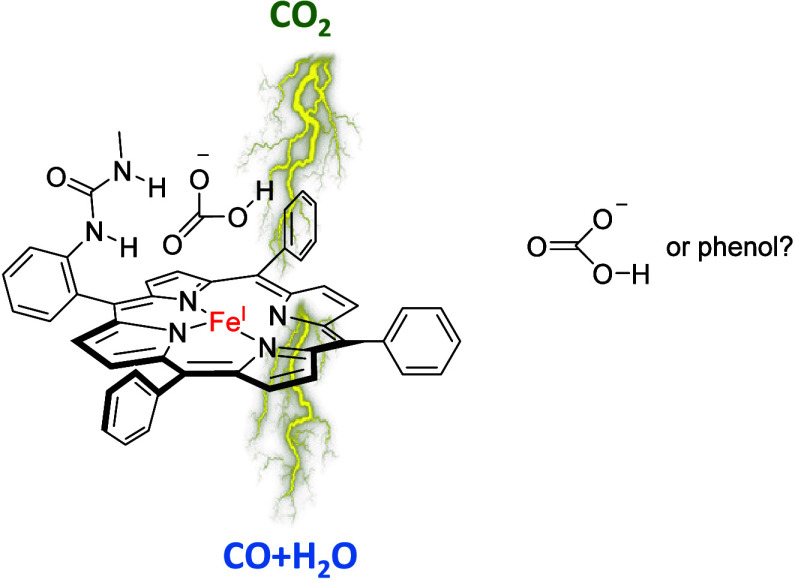

Transforming CO_2_ into valuable materials is an important reaction in catalysis, especially because CO_2_ concentrations in the atmosphere have been growing steadily due to extensive fossil fuel usage. From an environmental perspective, reduction of CO_2_ to valuable materials should be catalyzed by an environmentally benign catalyst and avoid the use of heavy transition-metal ions. In this work, we present a computational study into a novel iron(I) porphyrin catalyst for CO_2_ reduction, namely, with a tetraphenylporphyrin ligand and analogues. In particular, we investigated iron(I) tetraphenylporphyrin with one of the *meso*-phenyl groups substituted with *o*-urea, *p*-urea, or *o*-2-amide groups. These substituents can provide hydrogen-bonding interactions in the second coordination sphere with bound ligands and assist with proton relay. Furthermore, our studies investigated bicarbonate and phenol as stabilizers and proton donors in the reaction mechanism. Potential energy landscapes for double protonation of iron(I) porphyrinate with bound CO_2_ are reported. The work shows that the bicarbonate bridges the urea/amide groups to the CO_2_ and iron center and provides a tight bonding pattern with strong hydrogen-bonding interactions that facilitates easy proton delivery and reduction of CO_2_. Specifically, bicarbonate provides a low-energy proton shuttle mechanism to form CO and water efficiently. Furthermore, the *o*-urea group locks bicarbonate and CO_2_ in a tight orientation and helps with ideal proton transfer, while there is more mobility and lesser stability with an *o*-amide group in that position instead. Our calculations show that the *o*-urea group leads to reduction in proton-transfer barriers, in line with experimental observation. We then applied electric-field-effect calculations to estimate the environmental effects on the two proton-transfer steps in the reaction. These calculations describe the perturbations that enhance the driving forces for the proton-transfer steps and have been used to make predictions about how the catalysts can be further engineered for more enhanced CO_2_ reduction processes.

## Abstract 2

Density functional theory calculations show the importance of second-coordination-sphere effects for positioning of the substrate and proton donor in CO_2_ reduction processes.

## Introduction

The cumulative emission of CO_2_ is raising environmental concerns for its role in global warming, and consequently CO_2_ reduction is an urgent global issue. In the face of these serious environmental problems, the capture of CO_2_ has become a hot issue in recent years.^1−9^ However, apart from CO_2_ capture and storage, research has started into the utilization of CO_2_ for the synthesis of valuable chemicals and materials.^1−9^ Because CO_2_ is a cheap, abundant, and nontoxic C_1_ feedstock, it can be converted into various carbon resources such as alcohols, acids, esters, and hydrocarbons.^10−18^ This approach has 2-fold advantages, namely, CO_2_ reduction in the atmosphere as well as reduction in the use of unrenewable fossil fuels for synthesis of these compounds. Therefore, transforming CO_2_ into valuable chemicals may provide a solution to not only environmental problems caused by the emission of CO_2_ but also the anticipated depletion of fossil resources.

**Scheme 1:**
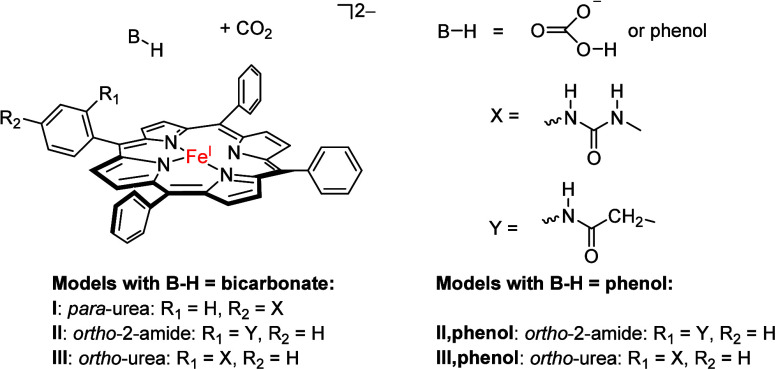
Models Investigated in This Work

Because CO_2_ reduction is a challenging reaction, often transition-metal catalysts are used for this process.^10−22^ In recent years, however, a number of catalysts with first-row transition-metal elements such as iron or manganese have been identified.^23−31^ Particularly promising CO_2_ reduction catalysts contain an iron(I) porphyrinate group.^32,33^ Thus, Chang et al. took an iron(I) tetraphenylporphyrin (TPP) complex and inserted substituents on the ortho and para positions of one of the phenyl groups (Scheme 1).^34^ These porphyrin derivatives with amide groups in the second coordination sphere were shown to give an enhanced reduction in CO_2_ to CO and water. They proposed a better positioning of CO_2_ on the iron center that enabled more efficient proton transfer from solution. Subsequent, density functional theory (DFT) calculations of some of us on CO_2_ reduction by [Fe(TPP)]^−^ and [Fe(*o*-2-amide-TPP)]^−^ showed the same electronic configurations and mechanisms for the catalytic cycle with small proton-transfer barriers from phenol.^35^ Recent work of Chang and co-workers used a system with a urea group linked to one of the *meso*-phenyl groups.^36^ In combination with bicarbonate as the proton donor, the authors measured a 1500-fold rate enhancement for CO_2_ reduction compared to that of a system without bicarbonate. They also determined a crystal structure of the bicarbonate-bound *o*-urea-TPP with Zn^2+^ as the central ion. To understand how and why bicarbonate can enhance the reaction rates for CO_2_ reduction processes with iron porphyrins, we decided to do a DFT study into the mechanism of the CO_2_ reduction process using an iron(I) porphyrin system with either *p*-urea, *o*-2-amide, or *o*-urea as substituents to one of the *meso*-phenyl groups of the porphyrin system with bicarbonate as the proton donor (models **I**–**III** in Scheme 1) and the models with *o*-2-amide and *o*-urea but with phenol as the proton donor (models **II,phenol** and **III,phenol**). The work shows that a hydrogen-bonding interaction in the second coordination sphere enhances proton-transfer rates. Moreover, the urea group can lock bicarbonate into a double hydrogen-bonding interaction mimicking a salt bridge in enzymatic structures that stabilizes the system and provides the best network for proton relay.

## Methods

The model was based on the CO_2_-bound optimized geometry of our previous studies on CO_2_ reduction by an [Fe^I^(TPP)] complex, where TPP = *meso*-tetraphenylporphyrin,^35^ whereby we manually inserted the *p*-urea, *o*-2-amide, or *o*-urea groups into one of the *meso*-phenyl substituents to create models **I**–**III** (Scheme 1) and replaced the other three *meso*-phenyl groups by a hydrogen atom. In addition, a molecule of bicarbonate was added to the distal site to create a cluster model with overall charge of 3– and odd multiplicity. To test whether the large negative charge in the model influences the structure and kinetics, we also used phenol as a proton donor because it has a p*K*_a_ value similar to that of bicarbonate, which gave the model an overall charge of 2–. Calculations were performed in *Gaussian 09* using the unrestricted B3LYP density functional method supplemented with the GD3 dispersion correction of Grimme, with Becke–Johnson damping included.^37−40^ An all-electron def2-SVP^41^ basis set was selected for all atoms during the geometry optimizations, analytical frequency calculations, and constraint geometry scans designated as BS1. Single-point energy calculations were performed with the def2-TZVP basis set on all atoms designated as BS2. Although the experimental work was performed in a dimethylformamide (DMF) solution, we tested several implicit solvent models with a range of dielectric constants of 37.2 (DMF) and 78.4 (water).^42^ In general, a change in the dielectric constant for the solvent model gives minor differences to the energies and optimized geometries and therefore does not change the results dramatically; see the Supporting Information. Calculations were performed on all low-lying triplet and quintet-spin-state surfaces. These methods and approaches were used before in our group and reproduced experimental free energies of activation within a few kilocalories per mole and predicted product selectivities in line with experimental observation.^43,44^

## Results and Discussion

**Figure 1:**
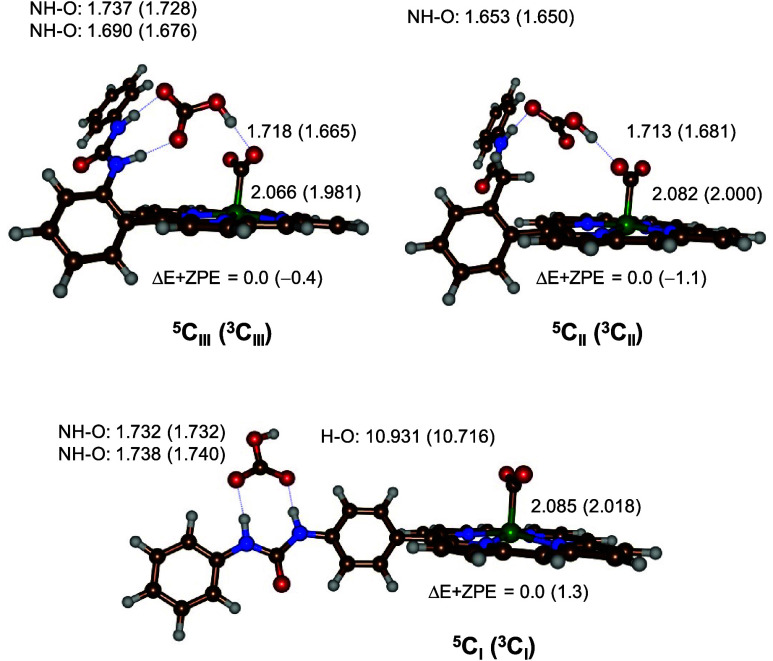
UB3LYP-GD3/BS1-optimized geometries of structure **C** for models **I**–**III**. Relative energies include energies at the BS2 level of theory and zero-point corrections in kilocalories per mole. Bond lengths are in angstroms.

**Scheme 2:**
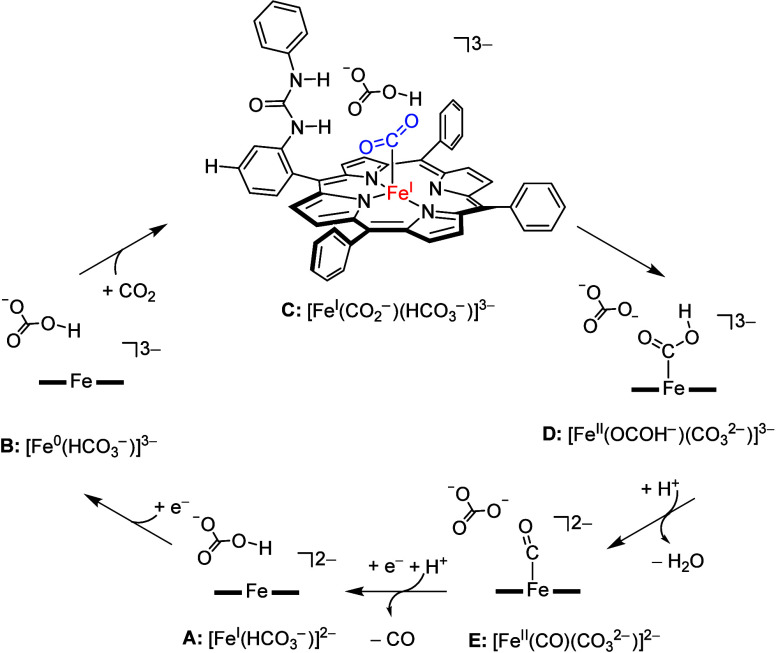
Catalytic Cycle of CO_2_ Reduction on a Bicarbonate-Bound Iron Porphyrinate System Studied in This Work Formal oxidation states of iron are given in square brackets.

The catalytic cycle for the reduction of CO_2_ to CO and water on an iron(I) porphyrinate complex as calculated here is shown in Scheme 2. The cycle starts from an iron(I) porphyrinate system with nearby bicarbonate, structure **A** or [Fe^I^(HCO_3_^–^)]^2–^. This complex is reduced from iron(I) to iron(0) to form **B** and triggers the binding of a molecule of CO_2_ and its reaction to give **C** or [Fe^I^(CO_2_^–^)(HCO_3_^–^)]^3–^. An internal proton transfer from bicarbonate to CO_2_ is expected to lead to protonated CO_2_ and carbonate in complex **D**: [Fe^II^(OCOH^–^)(CO_3_^2–^)]^3–^. The carbonate picks up a proton from the solvent/buffer and assists with the second protonation step of CO_2_ that leads to the formation of CO and water in complex **E**. The cycle is closed with the release of CO and the reduction of iron(II) to iron(I). In our previous studies,^35^ we showed that the first two steps in the CO_2_ reduction cycle, i.e., **A** → **B** and **B** → **C**, are little affected by second-coordination-sphere effects and substitution of the *meso*-phenyl groups of the porphyrin complex. Therefore, our work presented here is mostly focused on the protonation steps in the cycle and on how bicarbonate is involved in the process. In particular, we created CO_2_-bound iron(II) porphyrinate complexes with *p*-urea, *o*-2-amide, and *o*-urea as substituents to one of the phenyl groups on the *meso* position: structures **C**_**I**_, **C**_**II**_, and **C**_**III**_. Optimized geometries of structures **C** of models **I**–**III** in the triplet and quintet spin states are shown in Figure 1. The spin multiplicity is given as a superscript in front of the label and the model type as a subscript after the label.

In all structures, the triplet and quintet spin states are within 1.5 kcal mol^–1^, which implies that both may be accessible. Structures **II** and **III** have the same spin-state ordering and spin ground state; hence, these are not affected by the modification in the second coordination sphere. In the *p*-urea system (**I**), we find the quintet spin below the triplet spin state by 1.3 kcal mol^–1^ for structure **C**. Nevertheless, for all structures **C**, the triplet–quintet energy gap is within 1.3 kcal mol^–1^; consequently, the spin states are degenerate, and both will be accessible at room temperature conditions. We also calculated the CO_2_ binding energies for all complexes. In previous work, we reported CO_2_ binding energies of Δ*G* = 11.3 kcal mol^–1^ for the difference in energy between [Fe^I^(CO_2_^–^)(*o*-2-amide-TPP)]^2–^ and an isolated CO_2_ and [Fe^0^(*o*-2-amide-TPP)]^2–^ complex. With additional bicarbonate in the model in hydrogen-bonding interactions with CO_2_ in the **C**_**II**_ and **C**_**III**_ structures, CO_2_ release requires a free energy of Δ*G* = 9.6 and 9.2 kcal mol^–1^ in the triplet spin state, whereas we find Δ*G* = 4.6 and 3.5 kcal mol^–1^ in the quintet spin state. As such, the double hydrogen bond from urea to bicarbonate does not appear to dramatically influence the CO_2_ binding energy compared to the *o*-2-amide system. Despite the fact that the bicarbonate anion forms hydrogen-bonding interactions with the CO_2_ group bound with *o*-urea and *o*-2-amide, there does not appear to be a major stabilization energy as a result of this. As a matter of fact, our previous study also had a hydrogen bond to the bound CO_2_ group in [Fe^II^(CO_2_)(*o*-2-amide-TPP)]^2–^ but directly with the amide proton. However, the hydrogen-bonding network will facilitate the CO_2_ binding and lock it in position.

The structures shown in Figure 1 give tight binding of CO_2_ and bicarbonate between the iron(II) and hydrogen-bonding *o*-amide and *o*-urea groups. In the *p*-urea structure, the hydrogen-bonding groups are relatively far from the iron center by more than 10 Å, and, consequently, a bridge will require multiple water molecules, which were not included in the model. Nevertheless, the hydrogen-bonding network to the CO_2_ group only has a minor effect on the Fe–C bond lengths, which is 2.085 Å in ^**5**^**C**_**I**_ and 2.018 Å in ^**3**^**C**_**I**_, whereas the quintet-spin-state structures for ^**5**^**C**_**II**_ and ^**5**^**C**_**III**_ have values within 0.02 Å. These Fe–C distances are in line with those reported previously for the interaction of iron with a first-row element such as oxygen, nitrogen, or carbon.^45^ The optimized geometries compare well to those calculated before using the B97-D approach with strong hydrogen-bonding interactions between bicarbonate and the two NH groups of the urea substituent and the oxygen atom of CO_2_.^36^ The porphyrin scaffold is close to planarity, similar to previous calculations on porphyrin models in the gas phase.^46−50^

In model **III**, the bicarbonate ion is locked in position through two hydrogen-bonding interactions with the urea N–H groups, while its OH group forms a hydrogen bond to the CO_2_ group. This orientation is reminiscent of salt bridges in protein structures with a negatively charged carboxylate group opposite of a positively charged arginine side chain.^51−53^ However, different from a salt bridge in a protein structure, the urea system investigated here is charge-neutral; hence, the binding strength of the interaction with bicarbonate will be much weaker than that in a typical salt bridge. The two urea hydrogen-bonding interactions are different in length; namely, the one closest to the porphyrin is shortest at 1.690 Å in ^**5**^**C**_**III**_, while the other one has a distance of 1.737 Å. At the same time, the OH group of bicarbonate forms a hydrogen bond with one of the oxygen atoms of CO_2_ at 1.718 Å. These distances are very similar for the *o*-2-amide complex, but because it can form only two hydrogen bonds, the bicarbonate is lesser restraint.

**Figure 2:**
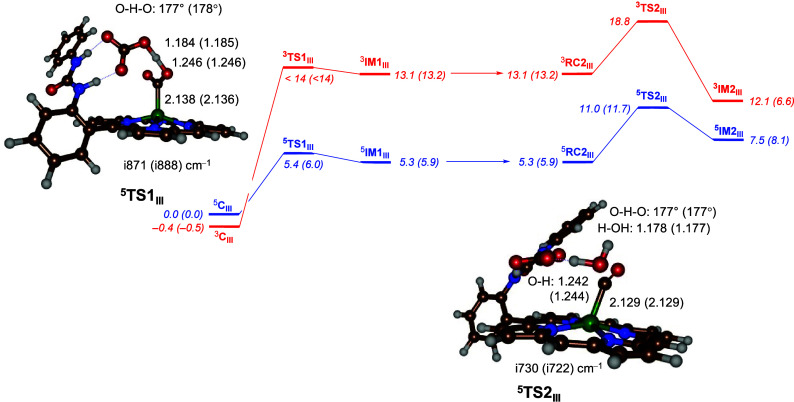
UB3LYP-GD3/BS2//UB3LYP-GD3/BS1 calculated the free energy profile for the two proton-transfer steps from the CO_2_-bound iron(II) porphyrin complex [Fe^II^(CO_2_)(*o*-urea-TPP)(HCO_3_^–^)]^3–^. Free energies (Δ*G*) were calculated at 298 K and include energies at the BS2 level of theory and zero-point, solvent, thermal, and entropic corrections in kilocalories per mole relative to ^**5**^**C**_**III**_, whereby calculations in the water solvent are the data outside parentheses and those in the DMF solvent are the data inside parentheses. Optimized transition-state geometries for the first and second proton transfer for model **III** are shown with bond lengths in angstroms, angles in degrees, and the imaginary frequency in reciprocal centimeters. In **RC2**, a proton is added to the model of **IM1** and the energy is set to the value of **IM1** for comparison.

Next, we studied the proton-transfer step from ^**5,3**^**C** (models **I**–**III**) to form an iron(II) with a protonated CO_2_ group (structures **IM1**) via transition state **TS_1_**. Thereafter, we took structures ^**5,3**^**IM1** and added a proton to the carbonate group to form the ^**5,3**^**RC2** complexes and searched for a proton-transfer transition state (**TS2**) to form water and CO products (structures **IM2**). The optimized transition-state structures, i.e., **TS1** and **TS_2_**, for the model **III** pathway are shown in Figure 2, together with the calculated energy landscapes. Both proton-transfer free energies of activation are small, namely, Δ*G*^⧧^ = 5.4 kcal mol^–1^ for the first proton transfer with respect to ^**5**^**C**_**III**_ and Δ*G*^⧧^ = 5.7 kcal mol^–1^ with respect to ^**5**^**RC2**_**III**_ (or Δ*G*^⧧^ = 11.0 kcal mol^–1^ with respect to ^**5**^**C**_**III**_). On the triplet spin state, the barriers are considerably higher in energy, namely, Δ*G*^⧧^ > 14 kcal mol^–1^ for the first proton transfer to reach ^**3**^**IM1**_**III**_ in an endergonic step of 13.1 kcal mol^–1^. On the triplet spin state, we were not able to fully characterize it transition state, but a constraint geometry scan shows it to be only slightly higher in energy than the value for ^**3**^**IM1**_**III**_; hence, a value of <14 kcal mol^–1^ is reported. The second proton transfer, similar to the quintet-spin-state surface, is 5.7 kcal mol^–1^. This is not surprising as no electron transfer happens in these steps and all group spin densities remain the same. Interestingly, for the **IM2**_**III**_ complex, the quintet-spin-state structure is higher in energy than the corresponding triplet spin complex. Thermal collisions of ^**5**^**IM2**_**III**_ in solution, however, are likely to revert the spin to the triplet spin ground state for the CO-bound structures **IM2**.

Structurally, the proton-transfer transition state ^**5**^**TS1**_**III**_ has the transferring proton midway between the donor and acceptor groups at distances of 1.184 and 1.246 Å. The O–H–O angle is close to linearity (177°), and the imaginary frequency of the transition state is i871 cm^–1^ for the proton-transfer mode. This is a relatively low imaginary frequency for proton transfer because usually values well over i1000 cm^–1^ are found.^54−56^ For ^**5**^**TS2**_**III**_, the imaginary frequency is similar (i730 cm^–1^) and represents simultaneous proton transfer and C–O cleavage, i.e., water release. The distance of the proton from the carbonate in ^**5**^**TS2**_**III**_ is 1.242 Å, while the distance to the OH group is 1.178 Å. Note that the Fe–C distances in ^**5**^**TS1**_**III**_ and ^**5**^**TS2**_**III**_ are similar (2.138 vs 2.129 Å, respectively), and consequently no electron transfer has happened in this process. Both energy landscapes were calculated with either water or DMF as an implicit solvent model. Both sets of data are shown in Figure 2, and as can be seen, the energy and structural differences are small when the solvent model is changed from water to DMF. These results match previous conclusions drawn from changing the value of the dielectric constant in implicit solvent calculations.^57^

**Figure 3:**
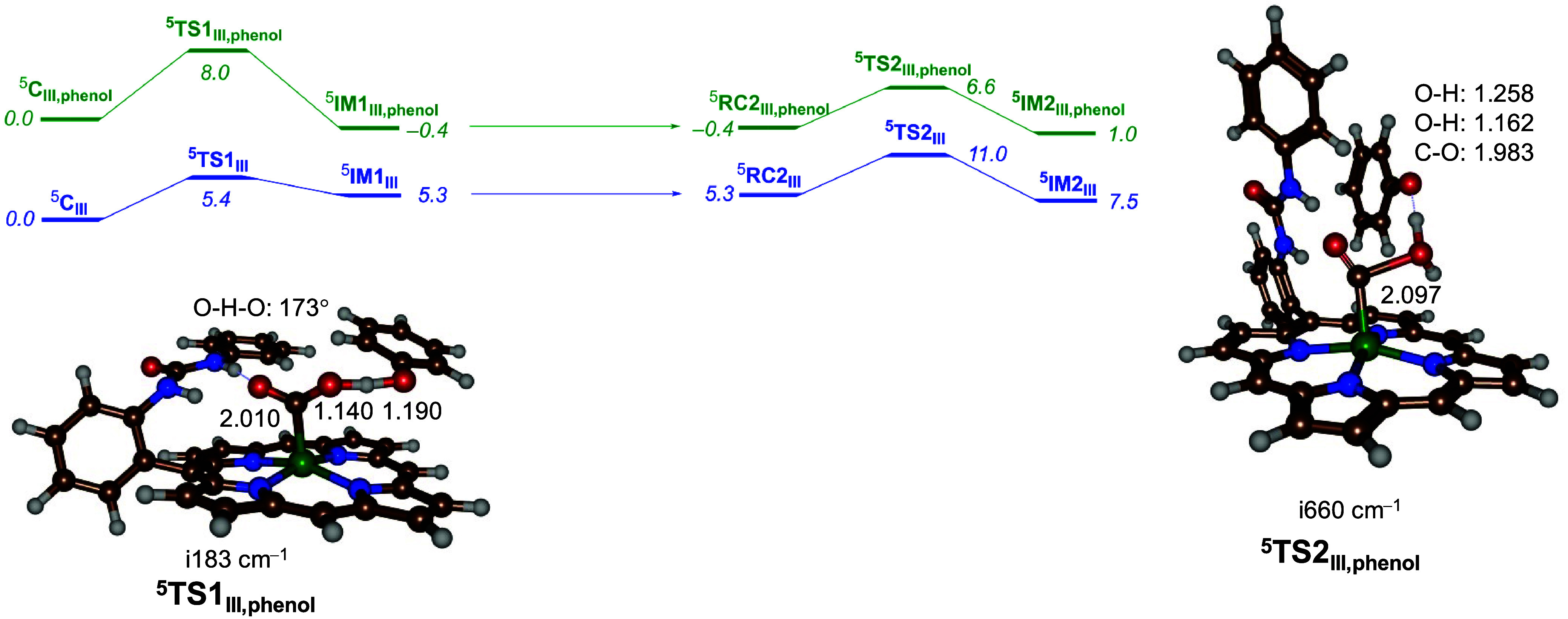
UB3LYP-GD3/BS2//UB3LYP-GD3/BS1 calculated the free energy profile for the two proton-transfer steps from the CO_2_-bound iron(II) porphyrin complex [Fe^II^(CO_2_)(*o*-urea-TPP)(phenol)]^2–^. The data for bicarbonate are in blue, whereas the data for phenol are in green. Free energies (Δ*G*) were calculated at 298 K and include energies at the BS2 level of theory and zero-point, solvent, thermal, and entropic corrections in kilocalories per mole relative to ^**5**^**C**_**III,phenol**_. Optimized transition-state geometries for the first and second proton transfer for model **III** are shown with bond lengths in angstroms, angles in degrees, and the imaginary frequency in reciprocal centimeters. In **RC2**, a proton is added to the model of **IM1**, and the energy is set to the value of **IM1** for comparison.

To find out how bicarbonate as a proton donor reacts compared to alternative proton donors, we removed bicarbonate from structures ^**3,5**^**C**_**III**_ and replaced it with a phenol molecule: ^**3,5**^**C**_**III,phenol**_. Subsequently, the proton-transfer pathways of CO_2_ were investigated, leading to CO and water as products. Figure 3 shows the optimized transition-state geometry for the first and second proton transfer on the quintet-spin-state surface with phenol as a proton donor. The obtained landscape for the phenol-bound structures compared to the bicarbonate reaction is shown in Figure 3, and the data for all local minima and transition states for all calculated models are reported in Table 1. The free energy of activation for the first proton transfer from ^**5**^**C**_**III,phenol**_ is Δ*G*^⧧^ = 8.0 kcal mol^–1^, while the second proton transfer was calculated at Δ*G*^⧧^ = 6.6 kcal mol^–1^ with respect to ^**5**^**C**_**III,phenol**_. Previously, for proton transfer from phenol to CO_2_ bound to Fe^II^(*o*-2-amide-TPP), we found a proton-transfer free energy of activation of 7.0 kcal mol^–1^ for the second proton-transfer step in the catalytic cycle, in agreement with what we find here with different methods and models.^35^ Taken together, the two consecutive proton-transfer steps for ^**5**^**C**_**III**_ have a maximum barrier of 11.0 kcal mol^–1^ using bicarbonate as a proton donor, while with phenol as the proton donor, the barrier is reduced to 8.0 kcal mol^–1^. Although the energy differences between the bicarbonate and phenol models are small, they do indicate a slowing down of the rate by a factor of 600 for the replacement of phenol by bicarbonate. This is not a surprising result because the p*K*_a_ value of the free bicarbonate/carbonate couple is 10.3, while the deprotonation of phenol has a p*K*_a_ value of 9.95.^58^ As such, free phenol should react with lower proton-transfer barriers than free bicarbonate, as indeed is observed here. These p*K*_a_ values, of course, will be affected upon binding to a hydrogen-bonding scaffold, such as *o*-urea, which can hold bicarbonate stronger than phenol. Despite the strong binding of bicarbonate to the urea scaffold, we see little effect on the rates based on the p*K*_a_ differences with phenol. The p*K*_a_ values of the *o*-urea and *o*-2-amide groups were calculated and found to be much higher than those for phenol. Therefore, the *o*-urea and *o*-2-amide groups cannot serve as proton donors in the reaction mechanisms because their N–H bonds are too strong to break. In none of the geometry optimizations reported here was a proton transfer from the *o*-urea or *o*-2-amide groups to either bicarbonate, CO_2_, or phenol observed. Therefore, the calculations show that a proton donor is needed and that phenol and bicarbonate are strong enough bases to provide protons for the CO_2_ reduction reaction.

**Table 1:** Free Energies (BS2//BS1) for Calculated Reaction Pathways for Double Protonation of CO_2_ Bound to Various Porphyrin Models^a^

	**III**	**III,phenol**	**II**	**II,phenol**^b^
^**5**^**RC**	0.0	0.0	0.0	0.0
^**5**^**TS1**	5.4	8.0	<5	<1
^**5**^**IM1**	5.3	–0.4	4.6	–11.4
^**5**^**RC2**	5.3	–0.4	4.6	–11.4
^**5**^**TS2**	11.0	6.6	30.7	7.0
^**5**^**IM2**	7.5	1.0	7.3	–14.4

a Δ*G*(BS2) values in kilocalories per mole.

b Data from ref (35).

The optimized transition-state structures ^**5**^**TS1**_**III,phenol**_ and ^**5**^**TS2**_**III,phenol**_ are also shown in Figure 3. The first proton-transfer transition state (^**5**^**TS1**_**III,phenol**_) has a small imaginary frequency of i183 cm^–1^ for proton transfer from phenol to CO_2_. The structure is relatively central, with the transferring proton midway between the donor (by 1.190 Å) and acceptor (by 1.140 Å) oxygen atoms, respectively. The angle O–H–O is close to linearity (173°), as is expected of a proton-transfer transition state. In the second proton-transfer transition state, the structure is more upright and the C–O bond has elongated significantly to 2.097 Å. The imaginary frequency of i660 cm^–1^ indicates dominant proton transfer from phenol to oxygen. The transferring proton is at a distance of 1.258 Å from phenolate and 1.162 Å from the accepting oxygen atom. Nevertheless, the calculations presented in Figures 2 and 3 show that both bicarbonate and phenol are strong enough acids to donate a proton to an Fe^II^-CO_2_ complex efficiently and initiate the CO_2_ reduction process. We observe a lowering of the first proton-transfer barrier for *o*-urea versus *o*-2-amide by 0.4 kcal mol^–1^, which would correspond to a rate enhancement of a factor of 2.4. Our work, therefore, is in good quantitative agreement with the experimental work of Chang et al., which showed enhanced reactivity by 2-fold between the two complexes with either *o*-urea or *o*-2-amide in the second coordination sphere.^36^

**Figure 4:**
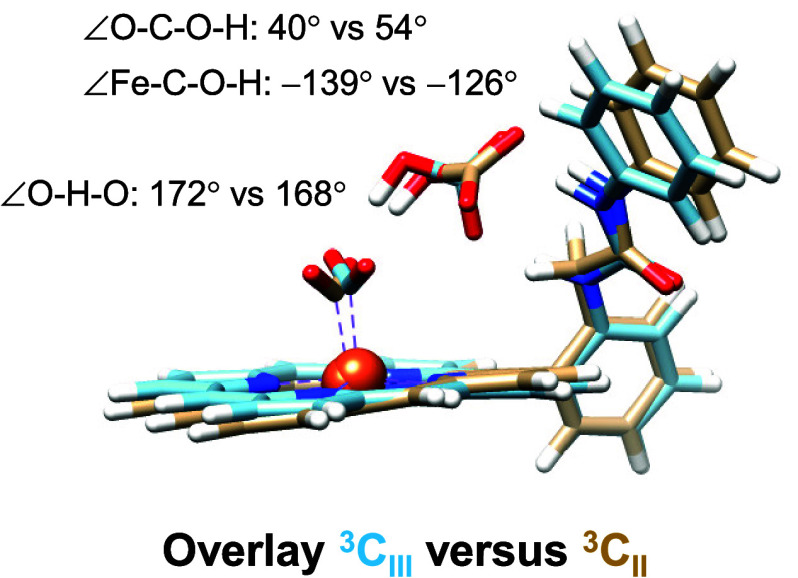
Overlay of the UB3LYP-GD3/BS1-optimized geometries. Angles and dihedral angles are in degrees.

Overall, the calculations presented in this work indicate that an *o*-urea substituent to a TPP scaffold enables strong hydrogen-bonding interactions that hold and position distal ligands to the iron center better than *o*-2-amide. To fully understand the positional differences, we created an overlay of the ^**3**^**C**_**II**_ and ^**3**^**C**_**III**_ optimized geometries and present the results in Figure 4. As can be seen, the two structures are seemingly similar and have most groups in a similar position and orientation. However, there are differences, as highlighted in the figure. Thus, the O–H–O angle is close to linearity in ^**3**^**C**_**III**_ at 172°, while the angle is 168° in ^**3**^**C**_**II**_. The closer to linearity the O–H–O angle is, the easier it is for proton transfer to take place. Indeed the smallest proton-transfer barrier is found for ^**3**^**C**_**II**_. Further differences between ^**3**^**C**_**II**_ and ^**3**^**C**_**III**_ relate to the positioning of the bicarbonate ion in the complex, as seen from the dihedral angles Fe–C–O–H and O–C–O–H in Figure 4. Both of these differ by more than 12° and show that bicarbonate is better positioned for proton transfer in ^**3**^**C**_**III**_ than in ^**3**^**C**_**II**_.

**Figure 5:**
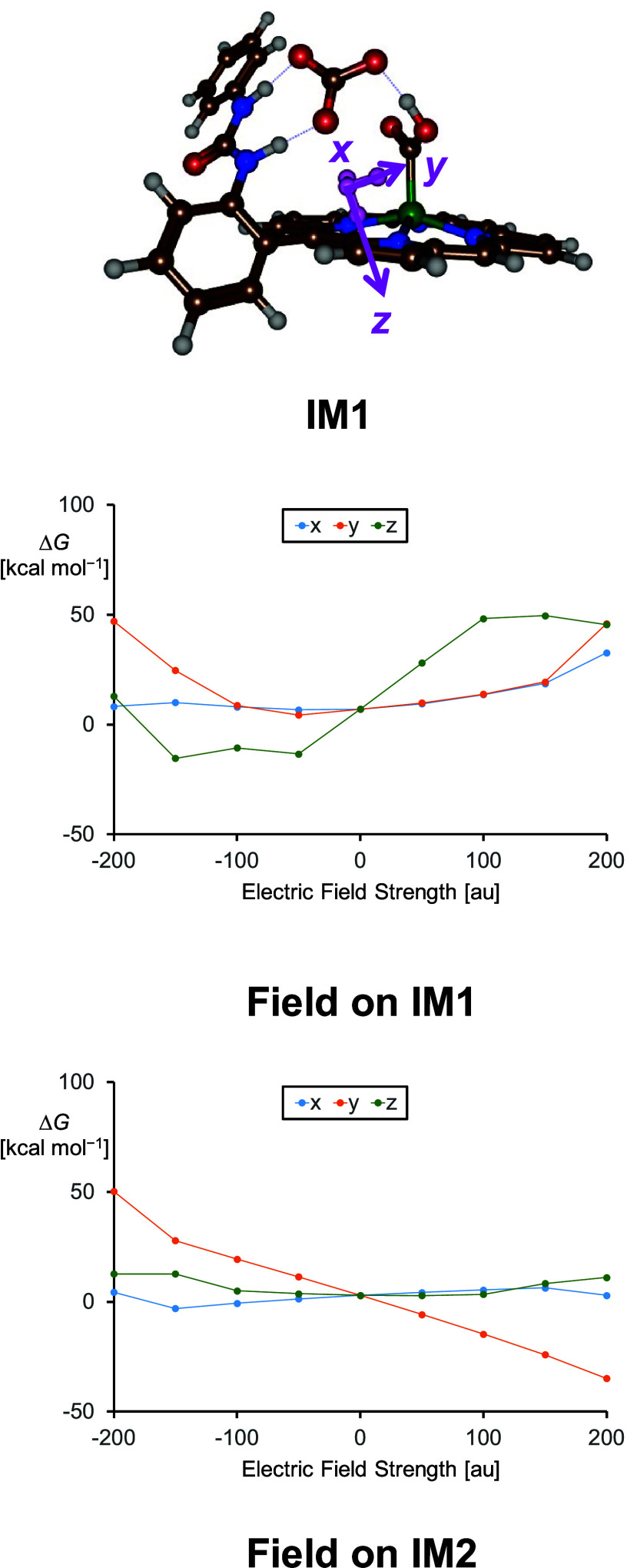
Electric-field effects on the stabilization free energies of ^**5**^**IM1** and ^**5**^**IM2** for model **II**. Electric fields are as defined in Gaussian.

To find out how these iron porphyrin systems can be improved for more efficient proton transfer, we decided to apply electric-field-effect perturbations to the optimized geometries of ^**5**^**C**_**III**_, ^**5**^**IM1**_**III**_, ^**5**^**RC2**_**III**_, and ^**5**^**IM2**_**III**_. In particular, single-point calculations at the UB3LYP-GD3/BS2 level of theory were run in Gaussian with an electric field located along the molecular *x*, *y*, or *z* axis, with magnitudes ranging from −200 to +200 au, and the results are shown in Figure 5. These electric-field perturbations were used previously in our group and shown to influence charge distributions in complexes and bifurcation patterns in chemical catalysis.^59,60^ In particular, recent work showed that charged groups in proteins can influence the strength of the C–H bonds in substrates and direct a reaction selectivity to a specific bond in a substrate.^61,62^ Thus, an electric-field perturbation has a major effect on the thermodynamics for proton transfer and the reaction energy. To be specific, an electric-field effect along the negative *z* axis is along the proton-transfer axis and makes the first proton-transfer step more exergonic, while a field in the opposite direction makes the reaction more endergonic, i.e., less likely. Interestingly, electric-field effects in the *x* and *y* directions do not appear to have a major effect on the first proton-transfer step unless very large electric fields are used. For the second proton-transfer step, a field along the positive *y* axis is favorable, while in the negative *y* direction, the reaction becomes more endothermic. The vector in the imaginary frequency of **TS2** for this reaction step is along the *y* axis and shows motions for the C–O stretch vibration, i.e., cleavage of the C–O bond as well as proton transfer. Also, for the second proton-transfer step, fields orthogonal to the proton transfer show few effects on the reaction thermochemistry. Overall, these calculations show that the first proton-transfer step is improved with an electric-field effect along the negative *z* axis, while the second proton-transfer step is probably faster with a field along the positive *y* axis. Therefore, engineering of the iron porphyrin complex by, for instance, latching the porphyrin to a metal surface through its axial ligand, may help with the first proton-transfer reaction in the CO_2_ activation reaction, while perturbations with charged groups along the *y* axis will enhance the second proton-transfer step.

## Conclusions

In this work, a computational study is presented on CO_2_ reduction to CO and water on several iron porphyrinate complexes. The calculations show that an iron porphyrinate system is an efficient catalyst for CO_2_ reduction reactions and particularly systems with a hydrogen-bonding donor in the second coordination sphere because that helps to position and tighten the substrate. The tight binding of the CO_2_ substrate enables two low-energy proton-transfer steps to form CO and water efficiently. Furthermore, bicarbonate can be locked in a position with an *o*-urea group attached to the ortho *meso* position of the ligand to provide a second-coordination-sphere environment for locking the proton donor (bicarbonate) and substrate (CO_2_) in a tight orientation for efficient proton shuttle and ultimately CO_2_ reduction purposes. Finally, electric-field calculations were performed on the complexes for the successive proton-transfer steps in the catalytic cycle. These calculations predict that the proton-transfer steps will be sensitive to local perturbations, and an electric-field-effect perturbation can influence driving forces for these steps and make the CO_2_ reduction reaction more exothermic and efficient.

## Supporting Information Available

Raw data including absolute and relative energies, group spin densities and charges, and Cartesian coordinates of optimized geometries discussed in this work (PDF)

## References

[ref1] Liu Z.; Qian J.; Zhang G.; Zhang B.; He Y. Electrochemical CO2-to-CO conversion: comprehensive review of recent developments and emerging trends. Separ. Purif. Technol. 2024, 330, 125177 10.1016/j.seppur.2023.125177.

[ref2] Wang C.; Lv Z.; Feng X.; Yang W.; Wang B. Recent advances in electrochemical CO2-to-multicarbon conversion: from fundamentals to industrialization. Adv. Energy Mater. 2023, 13, 2302382 10.1002/aenm.202302382.

[ref3] Darji H. R.; Kale H. B.; Shaikh F. F.; Gawande M. B. Advancement and state-of-art o heterogeneous catalysis of selective CO2 hydrogenation to methanol. Coord. Chem. Rev. 2023, 497, 215409 10.1016/j.ccr.2023.215409.

[ref4] Younus H. A.; Ahmad N.; Ni W.; Wang X.; Al-Abri M.; Zhang Y.; Verpoort F.; Zhang S. Molecular catalysts for CO_2_ Electroreduction: Progress and prospects with pincer type complexes. Coord. Chem. Rev. 2023, 493, 215318 10.1016/j.ccr.2023.215318.

[ref5] Chatterjee S.; Sengupta K.; Mondal B.; Dey S.; Dey A. Factors Determining the Rate and Selectivity of 4-/4H+ Electrocatalytic Reduction of Dioxygen by Iron Porphyrin Complexes. Acc. Chem. Res. 2017, 50, 1744–1753. 10.1021/acs.accounts.7b00192. 28686419

[ref6] Rao H.; Bonin J.; Robert M. Toward Visible-Light Photochemical CO_2_-to-CH_4_ Conversion in Aqueous Solutions Using Sensitized Molecular Catalysis. J. Phys. Chem. C 2018, 122, 13834–13839. 10.1021/acs.jpcc.8b00950.

[ref7] Franco F.; Pinto M. F.; Royo B.; Lloret-Fillol J. A Highly Active N-Heterocyclic Carbene Manganese(I) Complex for Selective Electrocatalytic CO_2_ Reduction to CO. Angew. Chem., Int. Ed. 2018, 57, 4603–4606. 10.1002/anie.201800705. PMC5947128 29481726

[ref8] Fernández S.; Franco F.; Martínez Belmonte M.; Friães S.; Royo B.; Luis J. M.; Lloret-Fillol J. Decoding the CO2 Reduction Mechanism of a Highly Active Organometallic Manganese Electrocatalyst: Direct Observation of a Hydride Intermediate and Its Implications. ACS Catal. 2023, 13, 10375–10385. 10.1021/acscatal.3c01430.

[ref9] Zhu C.; D’Agostino C.; de Visser S. P. Mechanism of CO_2_ Reduction to Methanol with H_2_ on an Iron(II)-scorpionate Catalyst. Chem. Eur. J. 2023, 29, e202302832 10.1002/chem.202302832. 37694535

[ref10] He M.; Sun Y.; Han B. Green Carbon Science: Scientific Basis for Integrating Carbon Resource Processing, Utilization, and Recycling. Angew. Chem., Int. Ed. 2013, 52, 9620–9633. 10.1002/anie.201209384. 23893613

[ref11] Aresta M.; Dibenedetto A.; Quaranta E. State of the art and perspectives in catalytic processes for CO2 conversion into chemicals and fuels: The distinctive contribution of chemical catalysis and biotechnology. J. Catal. 2016, 343, 2–45. 10.1016/j.jcat.2016.04.003.

[ref12] Franco F.; Fernández S.; Lloret-Fillol J. Advances in the Electrochemical Catalytic Reduction of CO_2_ with Metal Complexes. Curr. Opin. Electrochem. 2019, 15, 109–117. 10.1016/j.coelec.2019.04.002.

[ref13] Modak A.; Bhanja P.; Dutta S.; Chowdhury B.; Bhaumik A. Catalytic Reduction of CO_2_ into Fuels and Fine Chemicals. Green Chem. 2020, 22, 4002–4033. 10.1039/D0GC01092H.

[ref14] Ding P.; Zhao H.; Li T.; Luo Y.; Fan G.; Chen G.; Gao S.; Shi X.; Lu S.; Sun X. Metal-Based Electrocatalytic Conversion of CO_2_ to Formic Acid/Formate. J. Mater. Chem. A 2020, 8, 21947–21960. 10.1039/D0TA08393C.

[ref15] Zhang R.; Warren J. J. Recent Developments in Metalloporphyrin Electrocatalysts for Reduction of Small Molecules: Strategies for Managing Electron and Proton Transfer Reactions. ChemSusChem. 2021, 14, 293–302. 10.1002/cssc.202001914. 33064354

[ref16] Ghosh A. C.; Duboc C.; Gennari M. Synergy Between Metals for Small Molecule Activation: Enzymes and Bio-inspired Complexes. Coord. Chem. Rev. 2021, 428, 213606 10.1016/j.ccr.2020.213606.

[ref17] Kinzel N. W.; Werlé C.; Leitner W. Transition Metal Complexes as Catalysts for the Electroconversion of CO_2_: An Organometallic Perspective. Angew. Chem., Int. Ed. 2021, 60, 11628–11686. 10.1002/anie.202006988. PMC8248444 33464678

[ref18] Lei K.; Yu Xia B. Electrocatalytic CO_2_ Reduction: from Discrete Molecular Catalysts to Their Integrated Catalytic Materials. Chem. Eur. J. 2022, 28, e202200141 10.1002/chem.202200141. 35266602

[ref19] Francke R.; Schille B.; Roemelt M. Homogeneously Catalyzed Electroreduction of Carbon Dioxide-Methods, Mechanisms, and Catalysts. Chem. Rev. 2018, 118, 4631–4701. 10.1021/acs.chemrev.7b00459. 29319300

[ref20] Abdinejad M.; Hossain M. N.; Kraatz H.-B. Homogeneous and heterogeneous molecule arcatalysts for electrochemical reduction of carbon dioxide. RSC Adv. 2020, 10, 38013–38023. 10.1039/D0RA07973A. 35515175 PMC9057206

[ref21] Fors S. A.; Malapit C. A. Homogeneous Catalysis for the Conversion of CO_2_, CO, CH_3_OH, and CH_4_ to C_2+_ Chemicals via C–C Bond Formation. ACS Catal. 2023, 13, 4231–4249. 10.1021/acscatal.2c05517.

[ref22] Sadique A. R.; Brennessel W. W.; Holland P. L. Reduction of CO_2_ to CO using low-coordinate iron: Formation of a four-coordinate iron dicarbonyl complex and a bridging carbonate complex. Inorg. Chem. 2008, 47, 784–786. 10.1021/ic701914m. 18171059 PMC2474856

[ref23] Wei D.; Sang R.; Moazezbarabadi A.; Junge H.; Beller M. Homogeneous Carbon Capture and Catalytic Hydrogenation: Toward a Chemical Hydrogen Battery System. JACS Au 2022, 2, 1020–1031. 10.1021/jacsau.1c00489. 35647600 PMC9131476

[ref24] Zhao H.-Z.; Chang Y.-Y.; Liu C. Electrodes modified with iron porphyrin and carbon nanotubes: application to CO_2_ reduction and mechanism of synergistic electrocatalysis. J. Solid State Electrochem. 2013, 17, 1657–1664. 10.1007/s10008-013-2027-1.

[ref25] Mohamed E. A.; Zahran Z. N.; Naruta Y. Efficient electrocatalytic CO_2_ reduction with a molecular cofacial iron porphyrin dimer. Chem. Commun. 2015, 51, 16900–16903. 10.1039/C5CC04273A. 26359693

[ref26] Okabe Y.; Lee S. K.; Kondo M.; Masaoka S. Syntheses and CO_2_ reduction activities of π-expanded/extended iron porphyrin complexes. J. Biol. Inorg. Chem. 2017, 22, 713–725. 10.1007/s00775-017-1438-3. 28083656

[ref27] Sinha S.; Warren J. J. Unexpected solvent effect in electrocatalytic CO_2_ to CO conversion revealed using asymmetric metalloporphyrins. Inorg. Chem. 2018, 57, 12650–12656. 10.1021/acs.inorgchem.8b01814. 30212195

[ref28] Fukuzumi S.; Lee Y.-M.; Ahn H. S.; Nam W. Mechanisms of catalytic reduction of CO_2_ with heme and nonheme metal complexes. Chem. Sci. 2018, 9, 6017–6034. 10.1039/C8SC02220H. 30090295 PMC6053956

[ref29] Ogawa A.; Oohora K.; Gu W.; Hayashi T. Electrochemical CO_2_ reduction by a cobalt bipyricorrole complex: decrease of an overpotential value derived from monoanionic ligand character of the porphyrinoid species. Chem. Commun. 2019, 55, 493–496. 10.1039/C8CC08876D. 30548040

[ref30] Zhang R.; Warren J. J. Recent Developments in Metalloporphyrin Electrocatalysts for Reduction of Small Molecules: Strategies for Managing Electron and Proton Transfer Reactions. ChemSusChem. 2021, 14, 293–302. 10.1002/cssc.202001914. 33064354

[ref31] Domingo-Tafalla B.; Chatterjee T.; Palomares E. Recent advances in the rational designing of metalloporphyrinoid-based CO2 reduction catalysts: From molecular structural tuning to the application in catalysis. J. Porphyrins Phthalocyanines 2023, 27, 23–46. 10.1142/S1088424623300033.

[ref32] Bhugun I.; Lexa D.; Savéant J.-M. Catalysis of the Electrochemical Reduction of Carbon Dioxide by Iron(0) Porphyrins: Synergystic Effect of Weak Brönsted Acids. J. Am. Chem. Soc. 1996, 118, 1769–1776. 10.1021/ja9534462.

[ref33] Costentin C.; Passard G.; Robert M.; Savéant J.-M. Ultraefficient homogeneous catalyst for the CO2-to-CO electrochemical conversion. Proc. Natl. Acad. Sci. U.S.A. 2014, 111, 14990–14994. 10.1073/pnas.1416697111. 25288744 PMC4210317

[ref34] Nichols E. M.; Derrick J. S.; Nistanaki S. K.; Smith P. T.; Chang C. J. Positional effects of second-sphere amide pendants on electrochemical CO_2_ reduction catalyzed by iron porphyrins. Chem. Sci. 2018, 9, 2952–2960. 10.1039/C7SC04682K. 29732079 PMC5915798

[ref35] Davethu P. A.; de Visser S. P. CO_2_ reduction on an iron-porphyrin center: A computational study. J. Phys. Chem. A 2019, 123, 6527–6535. 10.1021/acs.jpca.9b05102. 31283234

[ref36] Derrick J. S.; Loipersberger M.; Nistanaki S. K.; Rothweiler A. V.; Head-Gordon M.; Nichols E. M.; Chang C. J. Templating Bicarbonate in the Second Coordination Sphere Enhances Electrochemical CO2 Reduction Catalyzed by Iron Porphyrins. J. Am. Chem. Soc. 2022, 144, 11656–11663. 10.1021/jacs.2c02972. 35749266

[ref37] Frisch M. J.; Trucks G. W.; Schlegel H. B.; Scuseria G. E.; Robb M. A.; Cheeseman J. R.; Scalmani G.; Barone V.; Mennucci B.; Petersson G. A.; Nakatsuji H.; Caricato M.; Li X.; Hratchian H. P.; Izmaylov A. F.; Bloino J.; Zheng G.; Sonnenberg J. L.; Hada M.; Ehara M.; Toyota K.; Fukuda R.; Hasegawa J.; Ishida M.; Nakajima T.; Honda Y.; Kitao O.; Nakai H.; Vreven T.; Montgomery J. A. Jr.; Peralta J. E.; Ogliaro F.; Bearpark M.; Heyd J. J.; Brothers E.; Kudin K. N.; Staroverov V. N.; Kobayashi R.; Normand R.; Raghavachari K.; Rendell A.; Burant J. C.; Iyengar S. S.; Tomasi J.; Cossi M.; Rega N.; Millam J. M.; Klene M.; Knox J. E.; Cross J. B.; Bakken V.; Adamo C.; Jaramillo J.; Gomperts R.; Stratmann R. E.; Yazyev O.; Austin A. J.; Cammi R.; Pomelli C.; Ochterski J. W.; Martin R. L.; Morokuma K.; Zakrzewski V. G.; Voth G. A.; Salvador P.; Dannenberg J. J.; Dapprich S.; Daniels A. D.; Farkas Ö.; Foresman J. B.; Ortiz J. V.; Cioslowski J.; Fox D. J. Gaussian 09, revision D.01; Gaussian, Inc.: Wallingford, CT, 2009.

[ref38] Becke A. D. Density-Functional thermochemistry. III. The Role of Exact Exchange. J. Chem. Phys. 1993, 98, 5648–5652. 10.1063/1.464913.

[ref39] Lee C.; Yang W.; Parr R. G. Development of the Colle-Salvetti Correlation-energy Formula Into a Functional of the Electron Density. Phys. Rev. B 1988, 37, 785–789. 10.1103/PhysRevB.37.785. 9944570

[ref40] Grimme S.; Antony J.; Ehrlich S.; Krieg H. A Consistent and Accurate Ab Initio Parametrization of Density Functional Dispersion Correction (DFT-D) for the 94 Elements H-Pu. J. Chem. Phys. 2010, 132, 154104 10.1063/1.3382344. 20423165

[ref41] Weigend F. Accurate Coulomb-fitting basis sets for H to Rn. Phys. Chem. Chem. Phys. 2006, 8, 1057–1065. 10.1039/b515623h. 16633586

[ref42] Tomasi J.; Mennucci B.; Cammi R. Quantum Mechanical Continuum Solvation Models. Chem. Rev. 2005, 105, 2999–3093. 10.1021/cr9904009. 16092826

[ref43] Mokkawes T.; de Visser S. P. Caffeine Biodegradation by Cytochrome P450 1A2. What Determines the Product Distributions?. Chem. Eur. J. 2023, 29, e202203875 10.1002/chem.202203875. 36929809

[ref44] Bagha U. K.; Yadav R.; Mokkawes T.; Satpathy J. K.; Kumar D.; Sastri C. V.; de Visser S. P. Defluorination of Fluorophenols by a Nonheme Iron(IV)-Oxo Species: Observation of a New Intermediate Along the Reaction. Chem. Eur. J. 2023, 29, e202300478 10.1002/chem.202300478. 37066848

[ref45] Faponle A. S.; Seebeck F. P.; de Visser S. P. Sulfoxide Synthase Versus Cysteine Dioxygenase Reactivity in a Nonheme Iron Enzyme. J. Am. Chem. Soc. 2017, 139, 9259–9270. 10.1021/jacs.7b04251. 28602090

[ref46] Colomban C.; Tobing A. H.; Mukherjee G.; Sastri C. V.; Sorokin A. B.; de Visser S. P. Mechanism of oxidative activation of fluorinated aromatic compounds by N-bridged diiron-phthalocyanine. What determines the reactivity?. Chem. Eur. J. 2019, 25, 14320–14331. 10.1002/chem.201902934. 31339185

[ref47] Ali H. S.; Henchman R. H.; de Visser S. P. Lignin biodegradation by a cytochrome P450 enzyme: A computational study into syringol activation by GcoA. Chem. Eur. J. 2020, 26, 13093–13102. 10.1002/chem.202002203. 32613677 PMC7590115

[ref48] Louka S.; Barry S. M.; Heyes D. J.; Mubarak M. Q. E.; Ali H. S.; Alkhalaf L. M.; Munro A. W.; Scrutton N. S.; Challis G. L.; de Visser S. P. The catalytic mechanism of aromatic nitration by cytochrome P450 TxtE: Involvement of a ferric-peroxynitrite intermediate. J. Am. Chem. Soc. 2020, 142, 15764–15779. 10.1021/jacs.0c05070. 32811149 PMC7586343

[ref49] Hermano Sampaio Dias A.; Yadav R.; Mokkawes T.; Kumar A.; Skaf M. S.; Sastri C. V.; Kumar D.; de Visser S. P. Biotransformation of Bisphenol by Human Cytochrome P450 2C9 Enzymes: a Density Functional Theory Study. Inorg. Chem. 2023, 62, 2244–2256. 10.1021/acs.inorgchem.2c03984. 36651185 PMC9923688

[ref50] Zhang Y.; Mokkawes T.; de Visser S. P. Insights into Cytochrome P450 Enzyme Catalyzed Defluorination of Aromatic Fluorides. Angew. Chem., Int. Ed. 2023, 62, e202310785 10.1002/anie.202310785. 37641517

[ref51] Berman H. M.; Westbrook J.; Feng Z.; Gilliland G.; Bhat T. N.; Weissig H.; Shindyalov I. N.; Bourne P. E. The Protein Data Bank. Nucleic Acids Res. 2000, 28, 235–242. 10.1093/nar/28.1.235. 10592235 PMC102472

[ref52] Cantú Reinhard F. G.; Lin Y.-T.; Stańczak A.; de Visser S. P. Bioengineering of cytochrome P450 OleTJE: How does substrate positioning affect the product distributions?. Molecules 2020, 25, 2675–2697. 10.3390/molecules25112675. 32526971 PMC7321372

[ref53] Pickl M.; Kurakin S.; Cantú Reinhard F. G.; Schmid P.; Pöcheim A.; Winkler C. K.; Kroutil W.; de Visser S. P.; Faber K. Mechanistic studies of fatty acid activation by CYP152 peroxygenases reveal unexpected desaturase activity. ACS Catal. 2019, 9, 565–577. 10.1021/acscatal.8b03733. 30637174 PMC6323616

[ref54] Li X.-X.; Postils V.; Sun W.; Faponle A. S.; Solà M.; Wang Y.; Nam W.; de Visser S. P. Reactivity patterns of (protonated) Compound II and Compound I of Cytochrome P450: Which is the better oxidant?. Chem. Eur. J. 2017, 23, 6406–6418. 10.1002/chem.201700363. 28295741

[ref55] Ghafoor S.; Mansha A.; de Visser S. P. Selective Hydrogen Atom Abstraction from Dihydroflavonol by a Non-Heme Iron Center Is the Key Step in the Enzymatic Flavonol Synthesis and Avoids Byproducts. J. Am. Chem. Soc. 2019, 141, 20278–20292. 10.1021/jacs.9b10526. 31749356

[ref56] Ali H. S.; Henchman R. H.; de Visser S. P. What Determines the Selectivity of Arginine Dihydroxylation by the Nonheme Iron Enzyme OrfP?. Chem. Eur. J. 2021, 27, 1795–1809. 10.1002/chem.202004019. 32965733

[ref57] Cantú Reinhard F. G.; Faponle A. S.; de Visser S. P. Substrate sulfoxidation by an iron(IV)-oxo complex: benchmarking computationally calculated barrier heights to experiment. J. Phys. Chem. A 2016, 120, 9805–9814. 10.1021/acs.jpca.6b09765. 27973805

[ref58] Lide D. R., Ed. Handbook of Chemistry and Physics, 76th ed.; CRC Press: Boca Raton, FL, 1996.

[ref59] de Visser S. P.; Mukherjee G.; Ali H. S.; Sastri C. V. Local charge distributions, electric dipole moments and local electric fields influence reactivity patterns and guide regioselectivities in α-ketoglutarate-dependent nonheme iron dioxygenases. Acc. Chem. Res. 2022, 55, 65–74. 10.1021/acs.accounts.1c00538. 34915695

[ref60] Gérard E. F.; Mokkawes T.; Johannissen L. O.; Warwicker J.; Spiess R. R.; Blanford C. F.; Hay S.; Heyes D. J.; de Visser S. P. How Is Substrate Halogenation Triggered by the Vanadium Haloperoxidase from *Curvularia inaequalis*?. ACS Catal. 2023, 13, 8247–8261. 10.1021/acscatal.3c00761. 37342830 PMC10278073

[ref61] Lin Y.-T.; Ali H. S.; de Visser S. P. Electrostatic perturbations from the protein affect C–H bond strengths of the substrate and enable negative catalysis in the TmpA biosynthesis enzyme. Chem. Eur. J. 2021, 27, 8851–8864. 10.1002/chem.202100791. 33978257

[ref62] Ali H. S.; Warwicker J.; de Visser S. P. How Does the Nonheme Iron Enzyme NapI React Through L-Arginine Desaturation Rather Than Hydroxylation? A QM/MM Study. ACS Catal. 2023, 13, 10705–10721. 10.1021/acscatal.3c02262.

