# Supplementary material for: CO2 Reduction by an Iron(I) Porphyrinate System: Effect of Hydrogen Bonding on the Second Coordination Sphere
Source: Inorg Chem. 2024 Feb 26;63(10):4474–81. doi: 10.1021/acs.inorgchem.3c04246 (PMC10934816; doi:10.1021/acs.inorgchem.3c04246)
Supplement: Supplementary file 1 — ic3c04246_si_001.pdf [file ic3c04246_si_001.pdf]

Supporting Information

CO_2 Reduction by an Iron(I)-Porphyrinate System. Effect of Hydrogen-Bonding in the Second-Coordination Sphere.

Chengxu Zhu,[§,⊥] Carmine D'Agostino,[⊥,†] and Sam P. de Visser*[§,⊥]

[§] Manchester Institute of Biotechnology, The University of Manchester, 131 Princess Street, Manchester M1 7DN, United Kingdom

[⊥] Department of Chemical Engineering, The University of Manchester, Oxford Road, Manchester M13 9PL, United Kingdom

[†] Dipartimento di Ingegneria Civile, Chimica, Ambientale e dei Materiali (DICAM), Alma Mater Studiorum – Università di Bologna, Via Terracini, 28, 40131 Bologna, Italy

Email: sam.devisser@manchester.ac.uk

Table S1: Absolute energies and free energies (in au) for UB3LYP/BS1 optimized geometries for CO_2 reduction on $[Fe^{II}$(*para*-urea-TPP)$(HCO_3)]^{3-}$ as calculated with a solvent model for water.

	E [au, BS1]	ZPE	G	E [au,BS2]
^{3}**RC1$_I$**	-3390.248081	0.517177	-3389.808482	-3392.771430
^{3}**TS1$_I$**	-3390.210858	0.512424	-3389.770416	-3392.728273
^{3}**IM1$_I$**	-3390.213738	0.516531	-3389.774541	-3392.741311
^{3}**RC2$_I$**	-3390.722105	0.529834	-3390.271874	-3393.237675
^{3}**TS2$_I$**	-3390.650343	0.521483	-3390.203464	-3393.171578
^{3}**IM2$_I$**	-3390.725327	0.526964	-3390.277999	-3393.257382
^{5}**RC1$_I$**	-3390.246143	0.514367	-3389.812304	-3392.767788
^{5}**IM1$_I$**	-3390.232596	0.516031	-3389.795801	-3392.763412
^{5}**RC2$_I$**	-3390.740666	0.529404	-3390.292551	-3393.259731
^{5}**TS2$_I$**	-3390.696508	0.522796	-3390.248325	-3393.218203
^{5}**IM2$_I$**	-3390.720615	0.524884	-3390.277177	-3393.250734

Table S2: Relative (free) energies (in kcal mol^{-1}) of the CO_2 reduction on $[Fe^{II}$(*para*-urea-TPP)$(HCO_3)]^{3-}$ as obtained with UB3LYP in Gaussian-09 as calculated with a solvent model for water.

	ΔE	ΔE+ZPE	ΔG	ΔE	ΔE+ZPE	ΔG
^{3}**RC1$_I$**	-1.22	0.55	2.40	-2.29	-0.52	1.33
^{3}**TS1$_I$**	22.14	20.92	26.29	24.80	23.58	28.94
^{3}**IM1$_I$**	20.33	21.69	23.70	16.61	17.97	19.98
^{3}**RC2$_I$**	20.33	21.69	23.70	16.61	17.97	19.98
^{3}**TS2$_I$**	77.01	73.40	79.60	71.93	68.32	74.52
^{3}**IM2$_I$**	29.96	29.79	32.83	18.09	17.92	20.96
^{5}**RC1$_I$**	0.00	0.00	0.00	0.00	0.00	0.00
^{5}**IM1$_I$**	8.50	9.54	10.36	2.75	3.79	4.60
^{5}**RC2$_I$**	8.50	9.54	10.36	2.75	3.79	4.60
^{5}**TS2$_I$**	36.21	33.11	38.11	28.81	25.70	30.70
^{5}**IM2$_I$**	21.08	19.29	20.00	8.39	6.60	7.31

Table S3: Group spin densities (a) and charges (b) of UB3LYP/BS1 optimized geometries for the CO_2 reduction on $[Fe^{II}$(*para*-urea-TPP)$(HCO_3)]^{3-}$ as obtained in Gaussian-09 as calculated with a solvent model for water.

a Spin densities

	Fe	CO_2	HCO_3(+H) or phenol (H)	Ligand	Total
^{3}**RC1$_{I}$**	1.47	-0.30	0.00	0.82	2.00
^{3}**TS1$_{I}$**	1.27	-0.18	-0.01	0.92	2.00
^{3}**IM1$_{I}$**	2.40	-0.25	-0.01	-0.14	2.00
^{3}**RC2$_{I}$**	2.41	-0.26	-0.01	-0.14	2.00
^{3}**TS2$_{I}$**	3.79	-0.02	0.00	-1.77	2.00
^{3}**IM2$_{I}$**	2.10	0.02	0.00	-0.12	2.00
^{5}**RC1$_{I}$**	3.48	-0.56	0.00	1.08	4.00
^{5}**IM1$_{I}$**	3.86	0.04	0.02	0.09	4.00
^{5}**RC2$_{I}$**	3.86	0.04	0.02	0.09	4.00
^{5}**TS2$_{I}$**	3.82	0.01	0.00	0.17	4.00
^{5}**IM2$_{I}$**	3.83	-0.03	0.00	0.20	4.00

b Charges

	Fe	CO_2	HCO_3(+H) or phenol (H)	Ligand	Total
^{3}**RC1$_{I}$**	0.43	-0.66	-0.70	-2.07	-3.00
^{3}**TS1$_{I}$**	0.45	-0.53	-0.65	-2.26	-3.00
^{3}**IM1$_{I}$**	0.53	-0.46	-0.99	-2.07	-3.00
^{3}**RC2$_{I}$**	0.53	-0.46	-0.56	-1.51	-2.00
^{3}**TS2$_{I}$**	0.67	-0.45	-0.88	-1.34	-2.00
^{3}**IM2$_{I}$**	0.66	-0.37	-1.11	-1.18	-2.00
^{5}**RC1$_{I}$**	0.61	-0.66	-0.70	-2.25	-3.00
^{5}**IM1$_{I}$**	0.66	-0.68	-1.02	-1.96	-3.00
^{5}**RC2$_{I}$**	0.67	-0.68	-0.59	-1.39	-2.00
^{5}**TS2$_{I}$**	0.69	-0.51	-0.85	-1.33	-2.00
^{5}**IM2$_{I}$**	0.70	-0.27	-1.11	-1.32	-2.00

Table S4: Absolute energies and free energies (in au) for UB3LYP/BS1 optimized geometries for CO_2 reduction on [Fe^{II}(*ortho*-amide-TPP)(HCO_3)]$^{3-}$and [Fe^{II}(*ortho*-amide-TPP)(phenol)]$^{2-}$ as calculated with a solvent model for water.

	E [au, BS1]	ZPE	G	E [au,BS2]
^{3}**RC1**$_{II}$	-3374.212254	0.529360	-3373.756903	-3376.714346
^{3}**IM1**$_{II}$	-3374.187553	0.527722	-3373.730808	-3376.690975
^{3}**RC2**$_{II}$	-3374.700142	0.543269	-3374.229872	-3377.195832
^{3}**TS2**$_{II}$	-3374.680789	0.536322	-3374.216755	-3377.186678
^{3}**IM2**$_{II}$	-3374.684841	0.539059	-3374.219660	-3377.194630
^{5}**RC1**$_{II}$	-3374.208810	0.527468	-3373.757039	-3376.708950
^{5}**IM1**$_{II}$	-3374.175759	0.525505	-3373.720855	-3376.676349
^{5}**RC2**$_{II}$	-3374.709692	0.541859	-3374.241734	-3377.202984
^{5}**TS2**$_{II}$	-3374.681829	0.535687	-3374.218624	-3377.184324
^{5}**IM2**$_{II}$	-3374.697956	0.540889	-3374.230227	-3377.195276
**Phenol structures:**				
^{5}**RC1**$_{II,phenol}$	-3417.155217	0.605707	-3416.628778	-3419.664064
^{5}**IM1**$_{II,phenol}$	-3417.150281	0.605957	-3416.621433	-3419.664669
^{5}**RC2**$_{II,phenol}$	-3417.643580	0.620294	-3417.100459	-3420.149708
^{5}**TS2**$_{II,phenol}$	-3417.635045	0.612161	-3417.100521	-3420.145753
^{5}**IM2**$_{II,phenol}$	-3417.636451	0.615621	-3417.099321	-3420.148614

Table S5: Relative (free) energies (in kcal mol^{-1}) of the CO_2 reduction on [Fe^{II}(*ortho*-amide-TPP)(HCO_3)]$^{3-}$ and [Fe^{II}(*ortho*-amide-TPP)(phenol)]$^{2-}$as obtained with UB3LYP in Gaussian-09 as calculated with a solvent model for water.

	ΔE	ΔE+ZPE	ΔG	ΔE	ΔE+ZPE	ΔG
^{3}**RC1**$_{II}$	-2.16	-0.97	0.09	-3.39	-2.20	-1.14
^{3}**IM1**$_{II}$	13.34	13.50	16.46	11.28	11.44	14.40
^{3}**RC2**$_{II}$	19.33	20.38	23.90	15.77	16.81	20.34
^{3}**TS2**$_{II}$	31.48	28.16	32.13	21.51	18.20	22.17
^{3}**IM2**$_{II}$	28.93	27.34	30.31	16.52	14.92	17.90
^{5}**RC1**$_{II}$	0.00	0.00	0.00	0.00	0.00	0.00
^{5}**IM1**$_{II}$	20.74	19.51	22.71	20.46	19.23	22.42
^{5}**RC2**$_{II}$	20.74	19.51	22.71	20.46	19.23	22.42
^{5}**TS2**$_{II}$	38.22	33.12	37.21	32.17	27.06	31.15
^{5}**IM2**$_{II}$	28.10	26.26	29.93	25.29	23.45	27.12
**Phenol structures**						
^{5}**RC1**$_{II,phenol}$	0.00	0.00	0.00	0.00	0.00	0.00
^{5}**IM1**$_{II,phenol}$	3.10	3.25	4.61	-0.38	-0.22	1.13
^{5}**RC2**$_{II,phenol}$	3.10	3.25	4.61	-0.38	-0.22	1.13
^{5}**TS2**$_{II,phenol}$	8.45	3.51	4.57	2.10	-2.84	-1.78
^{5}**IM2**$_{II,phenol}$	7.57	4.80	5.32	0.31	-2.47	-1.94

Table S6: Group spin densities (a) and charges (b) of UB3LYP/BS1 optimized geometries for the CO_2 reduction on $[Fe^{II}($*ortho*-amide-TPP$)(HCO_3)]^{3-}$ and $[Fe^{II}($*ortho*-amide-TPP$)(phenol)]^{2-}$ as obtained in Gaussian-09 as calculated with a solvent model for water.

a Spin densities

	Fe	CO_2	HCO_3 (+H) or phenol (+H)	Ligand	Total
^{3}**RC1$_{II}$**	1.44	-0.28	-0.01	0.85	2.00
^{3}**IM1$_{II}$**	1.25	-0.12	-0.05	0.92	2.00
^{3}**RC2$_{II}$**	2.09	0.09	0.02	-0.20	2.00
^{3}**TS2$_{II}$**	2.06	0.06	0.00	-0.12	2.00
^{3}**IM2$_{II}$**	2.08	0.04	0.01	-0.12	2.00
^{5}**RC1$_{II}$**	3.51	-0.57	-0.01	1.07	4.00
^{5}**IM1$_{II}$**	3.39	-0.46	-0.03	1.11	4.00
^{5}**RC2$_{II}$**	3.83	0.03	0.01	0.13	4.00
^{5}**TS2$_{II}$**	3.81	0.02	0.00	0.17	4.00
^{5}**IM2$_{II}$**	3.84	0.03	0.01	0.12	4.00
**Phenol structures:**					
^{5}**RC1$_{II,phenol}$**	3.42	-0.53	-0.01	1.12	4.00
^{5}**IM1$_{II,phenol}$**	3.78	0.07	0.01	0.15	4.00
^{5}**RC2$_{II,phenol}$**	3.84	0.01	0.01	0.14	4.00
^{5}**TS2$_{II,phenol}$**	3.82	-0.01	0.00	0.19	4.00
^{5}**IM2$_{phenol}$**	3.81	-0.02	0.00	0.20	4.00

b Charges

	Fe	CO_2	HCO_3 (+H) or phenol (+H)	Ligand	Total
^{3}**RC1$_{II}$**	0.43	-0.63	-0.88	-1.93	-3.00
^{3}**IM1$_{II}$**	0.43	-0.06	-1.06	-2.32	-3.01
^{3}**RC2$_{II}$**	0.56	-0.62	-0.72	-1.22	-2.00
^{3}**TS2$_{II}$**	0.59	-0.55	-0.93	-1.11	-2.00
^{3}**IM2$_{II}$**	0.62	-0.43	-1.08	-1.11	-2.00
^{5}**RC1$_{II}$**	0.62	-0.63	-0.86	-2.13	-3.00
^{5}**IM1$_{II}$**	0.60	-0.33	-1.23	-2.04	-3.00
^{5}**RC2$_{II}$**	0.64	-0.61	-0.69	-1.34	-2.00
^{5}**TS2$_{II}$**	0.67	-0.45	-0.96	-1.27	-2.00
^{5}**IM2$_{II}$**	0.65	-0.69	-0.69	-1.27	-2.00
**Phenol structures:**					
^{5}**RC1$_{II,phenol}$**	0.60	-0.54	-0.13	-1.93	-2.00
^{5}**IM1$_{II,phenol}$**	0.63	-0.74	-0.63	-1.26	-2.00
^{5}**RC2$_{II,phenol}$**	0.69	-0.60	0.07	-1.16	-1.00
^{5}**TS2$_{II,phenol}$**	0.69	-0.42	-0.17	-1.10	-1.00
^{5}**IM2$_{II,phenol}$**	0.69	-0.29	-0.34	-1.06	-1.00

Table S7: Absolute energies and free energies (in au) for UB3LYP/BS1 optimized geometries for CO_2 reduction on $[Fe^{II}$(*ortho*-urea-TPP)(HCO_3)$]^{3-}$ and $[Fe^{II}$(*ortho*-urea-TPP)(phenol)$]^{2-}$ as calculated with a solvent model for water.

	E [au, BS1]	ZPE	G	E [au,BS2]
^{3}**RC1**$_{III}$	-3390.262532	0.518645	-3389.815735	-3392.778835
^{3}**IM1**$_{III}$	-3390.231118	0.516905	-3389.785950	-3392.755585
^{3}**RC2**$_{III}$	-3390.744857	0.531822	-3390.287315	-3393.256873
^{3}**TS2**$_{III}$	-3390.728707	0.524477	-3390.277077	-3393.248885
^{3}**IM2**$_{III}$	-3390.737510	0.527691	-3390.283771	-3393.261655
^{5}**RC1**$_{III}$	-3390.259025	0.516552	-3389.816112	-3392.774277
^{5}**TS1**$_{III}$	-3390.235254	0.512092	-3389.796897	-3392.762239
^{5}**IM1**$_{III}$	-3390.237703	0.515055	-3389.796556	-3392.764000
^{5}**RC2**$_{III}$	-3390.751930	0.530144	-3390.295507	-3393.262716
^{5}**TS2**$_{III}$	-3390.730003	0.522734	-3390.280775	-3393.246485
^{5}**IM2**$_{III}$	-3390.744880	0.528856	-3390.289713	-3393.257989
**Phenol structures:**				
^{5}**RC1**$_{III,phenol}$	-3433.193609	0.594922	-3432.676730	-3435.721012
^{5}**TS1**$_{III,phenol}$	-3433.175740	0.590722	-3432.664994	-3435.702160
^{5}**IM1**$_{III,phenol}$	-3433.191416	0.594920	-3432.672901	-3435.723355
^{5}**RC2**$_{III,phenol}$	-3433.689313	0.609488	-3433.156911	-3436.212395
^{5}**TS2**$_{III,phenol}$	-3433.664690	0.602635	-3433.138839	-3436.194665
^{5}**IM2**$_{III,phenol}$	-3433.668609	0.605413	-3433.143552	-3436.202741

Table S8: Relative (free) energies (in kcal mol^{-1}) of the CO_2 reduction on $[Fe^{II}$(*ortho*-urea-TPP)(HCO_3)$]^{3-}$ and $[Fe^{II}$(*ortho*-urea-TPP)(phenol)$]^{2-}$as obtained with UB3LYP in Gaussian-09 as calculated with a solvent model for water.

	ΔE	ΔE+ZPE	ΔG	ΔE	ΔE+ZPE	ΔG
^{3}**RC1**$_{III}$	-2.20	-0.89	0.24	-2.86	-1.55	-0.42
^{3}**IM1**$_{III}$	17.51	17.73	18.93	11.73	11.95	13.14
^{3}**RC2**$_{III}$	17.51	17.73	18.93	11.73	11.95	13.14
^{3}**TS2**$_{III}$	32.08	28.75	30.49	20.41	17.07	18.82
^{3}**IM2**$_{III}$	26.56	25.24	26.29	12.39	11.08	12.13
^{5}**RC1**$_{III}$	0.00	0.00	0.00	0.00	0.00	0.00
^{5}**TS1**$_{III}$	14.92	12.12	12.06	7.55	4.75	4.69
^{5}**IM1**$_{III}$	13.38	12.44	12.27	6.45	5.51	5.34
^{5}**RC2**$_{III}$	13.38	12.44	12.27	6.45	5.51	5.34
^{5}**TS2**$_{III}$	27.14	21.55	21.52	16.63	11.04	11.01
^{5}**IM2**$_{III}$	17.80	16.06	15.91	9.41	7.67	7.52
**Phenol structures**						
^{5}**RC1**$_{III,phenol}$	0.00	0.00	0.00	0.00	0.00	0.00
^{5}**TS1**$_{III,phenol}$	11.21	8.58	7.36	11.83	9.19	7.98
^{5}**IM1**$_{III,phenol}$	1.38	1.37	2.40	-1.47	-1.47	-0.44
^{5}**RC2**$_{III,phenol}$	1.38	1.37	2.40	-1.47	-1.47	-0.44
^{5}**TS2**$_{III,phenol}$	16.83	12.53	13.74	9.66	5.35	6.57
^{5}**IM2**$_{III,phenol}$	14.37	11.81	10.79	4.59	2.03	1.00

Table S9: Group spin densities (a) and charges (b) of UB3LYP/BS1 optimized geometries for the CO_2 reduction on [Fe^{II}(*ortho*-urea-TPP)(HCO_3)]$^{3-}$ and [Fe^{II}(*ortho*-urea-TPP)(phenol)]$^{2-}$ as obtained in Gaussian-09 as calculated with a solvent model for water.

a Spin densities

	Fe	CO_2	HCO_3 (+H) or phenol (+H)	Ligand	Total
^{3}**RC1**$_{III}$	1.40	-0.26	-0.01	0.87	2.00
^{3}**IM1**$_{III}$	2.48	0.20	0.01	-0.68	2.00
^{3}**RC2**$_{III}$	2.09	0.08	0.02	-0.19	2.00
^{3}**TS2**$_{III}$	2.06	0.06	0.00	-0.12	2.00
^{3}**IM2**$_{III}$	2.09	0.03	0.01	-0.13	2.00
^{5}**RC1**$_{III}$	3.48	-0.56	-0.02	1.09	4.00
^{5}**TS1**$_{III}$	3.78	0.11	0.01	0.11	4.00
^{5}**IM1**$_{III}$	3.78	0.08	0.01	0.13	4.00
^{5}**RC2**$_{III}$	3.84	0.03	0.01	0.11	4.00
^{5}**TS2**$_{III}$	3.81	0.02	0.00	0.17	4.00
^{5}**IM2**$_{III}$	3.83	0.03	0.01	0.13	4.00
^{5}**RC1**$_{III,phenol}$	3.44	-0.55	0.00	1.11	4.00
^{5}**TS1**$_{III,phenol}$	3.39	-0.50	0.00	1.11	4.00
^{5}**IM1**$_{III,phenol}$	3.77	0.07	0.00	0.16	4.00
^{5}**RC2**$_{III,phenol}$	3.84	0.04	0.00	0.11	4.00
^{5}**TS2**$_{III,phenol}$	3.82	0.00	0.00	0.18	4.00
^{5}**IM2**$_{III,phenol}$	3.82	-0.02	0.00	0.20	4.00

b Charges

	Fe	CO_2	HCO_3 (+H) or phenol (+H)	Ligand	Total
^{3}**RC1**$_{III}$	0.44	-0.63	-0.80	-2.00	-3.00
^{3}**IM1**$_{III}$	0.55	-0.60	-1.23	-1.73	-3.00
^{3}**RC2**$_{III}$	0.54	-0.62	-0.64	-1.28	-2.00
^{3}**TS2**$_{III}$	0.59	-0.59	-0.81	-1.19	-2.00
^{3}**IM2**$_{III}$	0.65	-0.47	-0.98	-1.20	-2.00
^{5}**RC1**$_{III}$	0.62	-0.63	-0.79	-2.20	-3.00
^{5}**TS1**$_{III}$	0.59	-1.02	-0.99	-1.58	-3.00
^{5}**IM1**$_{III}$	0.62	-0.82	-1.19	-1.60	-3.00
^{5}**RC2**$_{III}$	0.65	-0.64	-0.64	-1.38	-2.00
^{5}**TS2**$_{III}$	0.68	-0.50	-0.84	-1.34	-2.00
^{5}**IM2**$_{III}$	0.65	-0.68	-0.64	-1.33	-2.00
^{5}**RC1**$_{III,phenol}$	0.60	-0.56	-0.12	-1.93	-2.00
^{5}**TS1**$_{III,phenol}$	0.59	-0.42	-0.36	-1.81	-2.00
^{5}**IM1**$_{III,phenol}$	0.61	-0.75	-0.56	-1.30	-2.00
^{5}**RC2**$_{III,phenol}$	0.65	-0.58	0.08	-1.14	-1.00
^{5}**TS2**$_{III,phenol}$	0.68	-0.46	-0.24	-0.98	-1.00
^{5}**IM2**$_{III,phenol}$	0.68	-0.29	-0.43	-0.96	-1.00

Table S10: Absolute energies and free energies (in au) for UB3LYP/BS1 optimized geometries for CO_2 reduction on $[Fe^{II}($*ortho*-urea-TPP$)(HCO_3)]^{3-}$ and $[Fe^{II}($*ortho*-urea-TPP$)($phenol$)]^{2-}$ as calculated with a solvent model for n,n-dimethylformamide.

	E [au, BS1]	ZPE	G	E [au,BS2]
^{3}**RC1**$_{III,\ solvent=DMF}$	-3390.255362	0.518591	-3389.808708	-3392.771589
^{3}**IM1**$_{III,\ solvent=DMF}$	-3390.223412	0.516713	-3389.779074	-3392.747434
^{3}**RC2**$_{III,\ solvent=DMF}$	-3390.741194	0.531932	-3390.281524	-3393.253102
^{3}**TS2**$_{III,\ solvent=DMF}$	-3390.724867	0.524373	-3390.273605	-3393.244814
^{3}**IM2**$_{III,\ solvent=DMF}$	-3390.733581	0.527599	-3390.280012	-3393.257509
^{5}**RC1**$_{III,\ solvent=DMF}$	-3390.251977	0.516526	-3389.808919	-3392.767188
^{5}**TS1**$_{III,\ solvent=DMF}$	-3390.227285	0.511907	-3389.789177	-3392.754086
^{5}**IM1**$_{III,\ solvent=DMF}$	-3390.229761	0.514884	-3389.788676	-3392.755866
^{5}**RC2**$_{III,\ solvent=DMF}$	-3390.748247	0.530114	-3390.291878	-3393.258964
^{5}**TS2**$_{III,\ solvent=DMF}$	-3390.726247	0.522719	-3390.276957	-3393.242596
^{5}**IM2**$_{III,\ solvent=DMF}$	-3390.741253	0.528838	-3390.286045	-3393.254297

Table S11: Relative (free) energies (in kcal mol^{-1}) of the CO_2 reduction on $[Fe^{II}($*ortho*-urea-TPP$)(HCO_3)]^{3-}$ and $[Fe^{II}($*ortho*-urea-TPP$)($phenol$)]^{2-}$as obtained with UB3LYP in Gaussian-09 as calculated with a solvent model for n,n-dimethylformamide (DMF).

	ΔE	ΔE+ZPE	ΔG	ΔE	ΔE+ZPE	ΔG
^{3}**RC1**$_{III,\ solvent=DMF}$	-2.12	-0.83	0.13	-2.76	-1.47	-0.50
^{3}**IM1**$_{III,\ solvent=DMF}$	17.93	18.04	18.73	12.40	12.51	13.20
^{3}**RC2**$_{III,\ solvent=DMF}$	17.93	18.04	18.73	12.40	12.51	13.20
^{3}**TS2**$_{III,\ solvent=DMF}$	28.17	23.54	23.70	17.60	12.97	13.12
^{3}**IM2**$_{III,\ solvent=DMF}$	22.70	20.10	19.68	9.63	7.03	6.60
^{5}**RC1**$_{III,\ solvent=DMF}$	0.00	0.00	0.00	0.00	0.00	0.00
^{5}**TS1**$_{III,\ solvent=DMF}$	15.49	12.60	12.39	8.22	5.32	5.12
^{5}**IM1**$_{III,\ solvent=DMF}$	13.94	12.91	12.70	7.10	6.07	5.87
^{5}**RC2**$_{III,\ solvent=DMF}$	13.94	12.91	12.70	7.10	6.07	5.87
^{5}**TS2**$_{III,\ solvent=DMF}$	27.75	22.08	22.07	17.38	11.70	11.69
^{5}**IM2**$_{III,\ solvent=DMF}$	18.33	16.50	16.36	10.03	8.20	8.07

Table S12: Group spin densities (a) and charges (b) of UB3LYP/BS1 optimized geometries for the CO_2 reduction on $[Fe^{II}($*ortho*-urea-TPP)$(HCO_3)]^{3-}$ and $[Fe^{II}($*ortho*-urea-TPP)(phenol)$]^{2-}$ as obtained in Gaussian-09 as calculated with a solvent model for n,n-dimethylformamide (DMF).

a Spin densities

	Fe	CO_2	HCO_3 (+H) or phenol (+H)	Ligand	Total
^{3}**RC1**$_{III, solvent=DMF}$	1.40	-0.26	-0.01	0.87	2.00
^{3}**IM1**$_{III, solvent=DMF}$	2.49	0.20	0.01	-0.70	2.00
^{3}**RC2**$_{III, solvent=DMF}$	2.10	0.08	0.02	-0.19	2.00
^{3}**TS2**$_{III, solvent=DMF}$	2.06	0.06	0.00	-0.12	2.00
^{3}**IM2**$_{III, solvent=DMF}$	2.09	0.03	0.01	-0.13	2.00
^{5}**RC1**$_{III, solvent=DMF}$	3.48	-0.56	-0.02	1.09	4.00
^{5}**TS1**$_{III, solvent=DMF}$	3.78	0.11	0.01	0.10	4.00
^{5}**IM1**$_{III, solvent=DMF}$	3.78	0.08	0.01	0.13	4.00
^{5}**RC2**$_{III, solvent=DMF}$	3.84	0.03	0.01	0.11	4.00
^{5}**TS2**$_{III, solvent=DMF}$	3.81	0.02	0.00	0.17	4.00
^{5}**IM2**$_{III, solvent=DMF}$	3.83	0.03	0.01	0.13	4.00

b Charges

	Fe	CO_2	HCO_3 (+H) or phenol (+H)	Ligand	Total
^{3}**RC1**$_{III, solvent=DMF}$	0.44	-0.63	-0.80	-2.01	-3.00
^{3}**IM1**$_{III, solvent=DMF}$	0.55	-0.59	-1.22	-1.74	-3.00
^{3}**RC2**$_{III, solvent=DMF}$	0.54	-0.62	-0.64	-1.28	-2.00
^{3}**TS2**$_{III, solvent=DMF}$	0.59	-0.58	-0.81	-1.19	-2.00
^{3}**IM2**$_{III, solvent=DMF}$	0.65	-0.47	-0.98	-1.20	-2.00
^{5}**RC1**$_{III, solvent=DMF}$	0.62	-0.63	-0.78	-2.21	-3.00
^{5}**TS1**$_{III, solvent=DMF}$	0.59	-1.01	-0.99	-1.60	-3.00
^{5}**IM1**$_{III, solvent=DMF}$	0.62	-0.82	-1.19	-1.61	-3.00
^{5}**RC2**$_{III, solvent=DMF}$	0.65	-0.63	-0.64	-1.38	-2.00
^{5}**TS2**$_{III, solvent=DMF}$	0.68	-0.49	-0.84	-1.35	-2.00
^{5}**IM2**$_{III, solvent=DMF}$	0.65	-0.68	-0.64	-1.33	-2.00

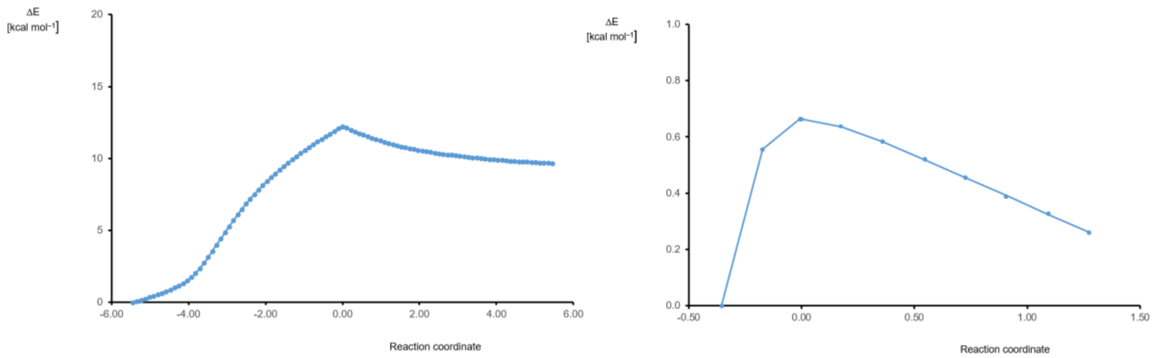

Triplet spin IRC **Quintet spin IRC**

Figure S1: Intrinsic reaction coordinate scans starting from $^{3,5}TS2_{II}$ in the direction of reactants and products. Data obtained at UB3LYP/BS1 in Gaussian.

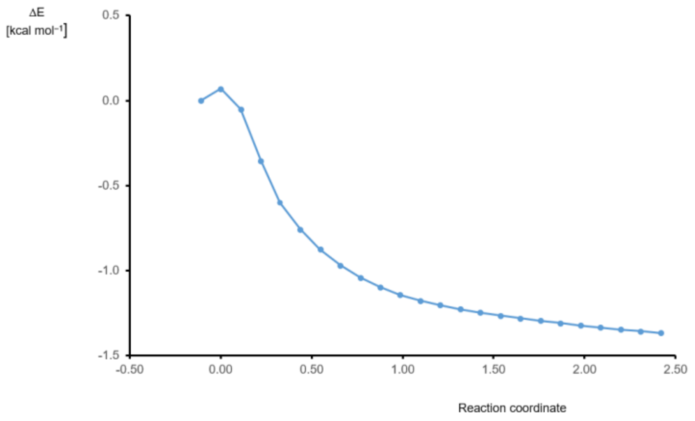

Figure S2: Intrinsic reaction coordinate scans starting from $^{5}TS1_{III}$ in the direction of reactants and products. Data obtained at UB3LYP/BS1 in Gaussian.

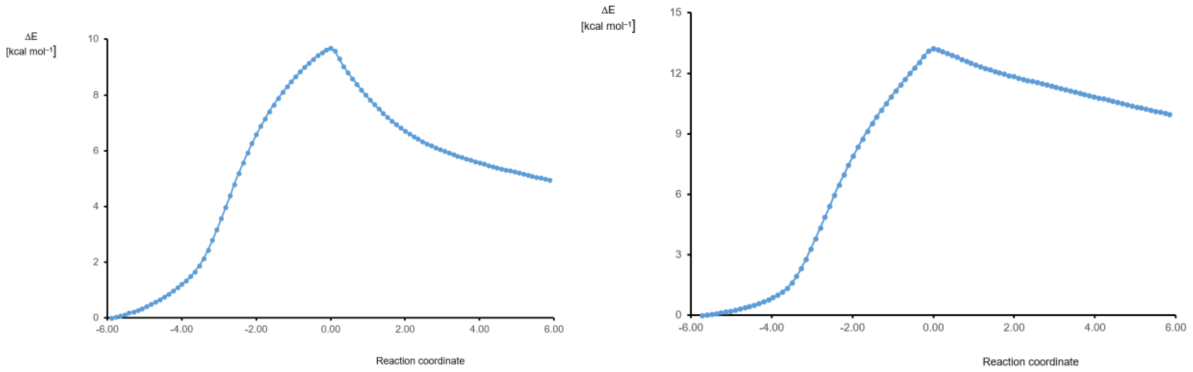

Triplet spin IRC **Quintet spin IRC**

Figure S3: Intrinsic reaction coordinate scans starting from $^{3,5}TS2_{III}$ in the direction of reactants and products. Data obtained at UB3LYP/BS1 in Gaussian.

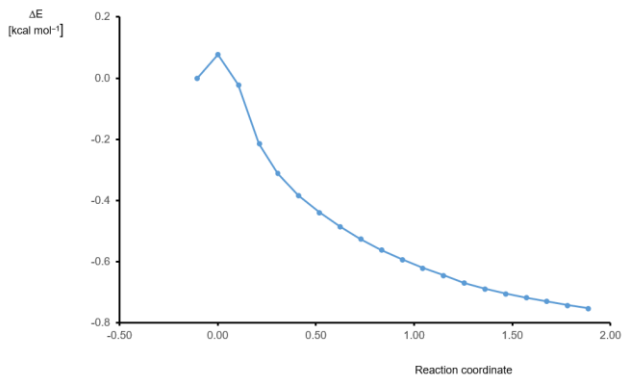

Figure S4: Intrinsic reaction coordinate scans starting from ^{5}TS2$_{II,\ phenol}$ in the direction of reactants and products. Data obtained at UB3LYP/BS1 in Gaussian.

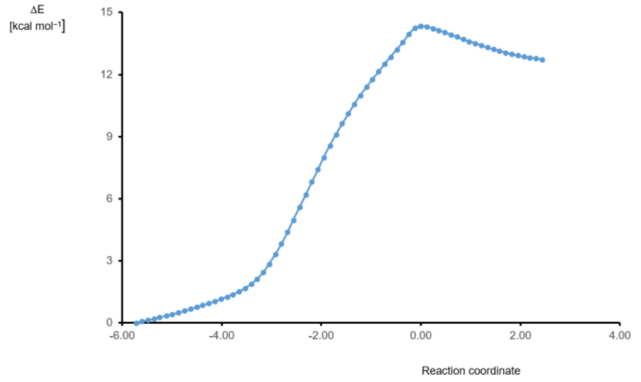

Figure S5: Intrinsic reaction coordinate scans starting from ^{5}TS2$_{III,\ phenol}$ in the direction of reactants and products. Data obtained at UB3LYP/BS1 in Gaussian.

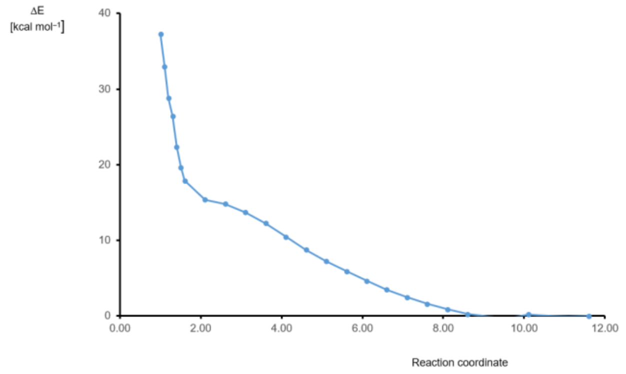

Figure S6: UB3LYP/BS1 calculated geometry scan for the C–H distance starting from ^{5}IM1$_{I}$ on the left-hand-side to ^{5}RC1$_{I}$. As can be seen no proton transfer barrier is encountered for the pathway from ^{5}IM1$_{I}$ to ^{5}RC1$_{I}$.

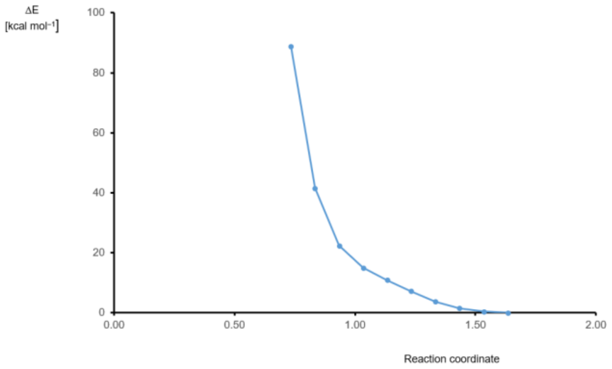

Figure S7: UB3LYP/BS1 calculated geometry scan for the C–H distance starting from ^{5}IM1$_{II,phenol}$ on the left-hand-side to ^{5}RC1$_{II,phenol}$. As can be seen no proton transfer barrier is encountered for the pathway from ^{5}IM1$_{II,phenol}$ to ^{5}RC1$_{II,phenol}$.

Cartesian coordinates of optimized geometries:

[Fe^{II}(*para*-urea-TPP)(HCO_3)]$^{3-}$

Solvent = water:

^{3}RC1$_{I}$:

26	-1.021828000	-0.722150000	20.406669000
8	-6.012625000	-8.411374000	16.135755000
7	-0.405750000	-2.610509000	19.995942000
7	0.562472000	-0.443033000	21.629714000
7	-4.097585000	-7.628023000	15.115422000
7	-5.353744000	-9.230359000	14.080950000
7	-1.849364000	0.975517000	21.200202000
7	-2.730758000	-1.129435000	19.431445000
6	-1.260898000	1.889966000	22.058686000
6	-1.021272000	-3.559880000	19.190057000
6	-3.789133000	-0.255540000	19.278034000
6	1.643878000	-1.287061000	21.759834000
6	-3.030681000	-2.236116000	18.640765000
6	-2.113266000	3.014413000	22.256547000
1	2.672499000	-3.108551000	21.409041000
6	-3.052905000	1.531247000	20.834754000
6	-3.245597000	2.789914000	21.484802000
6	-3.958958000	0.957582000	19.918267000
6	-0.219522000	-4.737827000	19.118165000
6	0.787373000	-3.170695000	20.394200000
1	-4.871311000	1.517388000	19.700042000
1	0.373897000	2.539164000	23.282489000
6	0.919405000	-4.491302000	19.871738000
6	-2.262640000	-3.385483000	18.519785000
6	1.763074000	-2.541754000	21.196739000
6	-4.292882000	-2.019351000	17.950959000
6	0.848407000	0.662632000	22.420900000
6	-2.057629000	-4.888411000	16.495959000
6	-2.754118000	-4.496608000	17.654138000
6	0.017631000	1.740230000	22.627788000
6	-4.760612000	-0.804310000	18.348476000
6	2.645176000	-0.692070000	22.632420000
6	2.153253000	0.511388000	23.039085000
6	-3.706455000	-6.614867000	15.994497000
6	-5.220954000	-8.421994000	15.191440000
6	-3.930932000	-5.199233000	17.957360000
6	-2.517709000	-5.918054000	15.680765000
6	-4.410272000	-6.237626000	17.154767000
1	-3.420671000	-7.813528000	14.345819000
1	-4.719094000	-9.055502000	13.272707000
1	-1.883494000	3.869275000	22.892851000
1	-4.122750000	3.426542000	21.366753000
1	-0.478408000	-5.642007000	18.573014000
1	1.766290000	-5.153632000	20.052150000
1	-4.751120000	-2.719871000	17.257427000
1	-5.682397000	-0.304141000	18.050984000
1	3.592204000	-1.162967000	22.896795000
1	2.607965000	1.241795000	23.708487000
1	-4.490440000	-4.926870000	18.855830000
1	-1.969391000	-6.200884000	14.779745000
1	-5.320501000	-6.769184000	17.417760000
6	0.050617000	0.036218000	18.875014000
8	-0.603141000	1.003607000	18.465839000
8	1.115265000	-0.478352000	18.538458000
1	-0.918404000	-8.135305000	11.322563000
8	-1.758996000	-8.492563000	10.998191000
8	-3.777924000	-8.885154000	11.828671000
6	-2.626824000	-8.462839000	12.066505000
8	-2.173642000	-8.021193000	13.150359000
6	-6.308196000	-10.223347000	13.856518000
6	-6.253840000	-10.889476000	12.610325000
6	-7.301015000	-10.607005000	14.782135000
6	-7.161745000	-11.900857000	12.303683000
6	-8.201831000	-11.625570000	14.457538000
6	-8.146291000	-12.280898000	13.224256000
1	-5.483758000	-10.592666000	11.895393000
1	-7.354669000	-10.096757000	15.739612000
1	-7.097755000	-12.399017000	11.332466000
1	-8.963808000	-11.907671000	15.189569000
1	-8.857536000	-13.073990000	12.982557000
1	-1.137455000	-4.364670000	16.226505000

^{3}TS1$_{I}$:

26	-1.520658000	-1.706719000	19.581684000
8	-5.188307000	-9.685990000	16.206445000
7	-0.778298000	-3.225374000	20.650957000
7	0.161824000	-0.651396000	19.937937000
7	-3.014793000	-8.908558000	16.332246000
7	-4.407165000	-8.055374000	14.715690000
7	-2.326449000	-0.069670000	18.784393000
7	-3.319243000	-2.552739000	19.774755000
6	-1.654396000	1.021098000	18.288095000
6	-1.312273000	-4.480201000	20.720385000
6	-4.539495000	-1.948761000	19.484289000
6	1.258503000	-1.039323000	20.699403000
6	-3.581418000	-3.890627000	19.967341000
6	-2.588515000	1.944665000	17.675213000
1	2.283631000	-2.425221000	21.945712000
6	-3.677518000	0.172123000	18.553703000
6	-3.842164000	1.426712000	17.863879000
6	-4.702122000	-0.672272000	18.933254000
6	-0.440579000	-5.347605000	21.476220000
6	0.428971000	-3.271567000	21.345536000
1	-5.723929000	-0.356795000	18.709058000
1	0.119396000	2.186088000	18.035156000
6	0.628264000	-4.585045000	21.886457000
6	-2.566014000	-4.878242000	20.186459000
6	1.360123000	-2.241749000	21.391218000
6	-4.993491000	-4.120234000	19.866011000
6	0.527487000	0.514870000	19.312400000
6	-1.538023000	-6.646350000	18.811975000
6	-2.657118000	-6.213588000	19.566226000
6	-0.295699000	1.274469000	18.468734000
6	-5.589149000	-2.901821000	19.589910000
6	2.311776000	-0.075763000	20.573276000
6	1.868015000	0.884167000	19.691658000
6	-2.878455000	-8.228096000	17.551858000
6	-4.325022000	-8.932449000	15.729274000
6	-3.825138000	-6.995261000	19.424331000
6	-1.632341000	-7.644223000	17.856053000
6	-3.944803000	-7.976139000	18.436068000
1	-2.337515000	-8.517299000	15.668099000
1	-3.521127000	-6.691426000	15.026517000
1	-2.308185000	2.884763000	17.200769000
1	-4.801802000	1.849859000	17.567371000
1	-0.644671000	-6.394949000	21.690655000
1	1.483264000	-4.886427000	22.491476000
1	-5.499777000	-5.068501000	20.004790000
1	-6.646517000	-2.690569000	19.431428000
1	3.275746000	-0.138176000	21.078073000
1	2.391845000	1.771489000	19.336953000
1	-4.687380000	-6.808134000	20.063316000
1	-0.778528000	-7.857508000	17.211568000
1	-4.891605000	-8.496201000	18.288717000
6	-1.079400000	-2.639188000	17.919170000
8	-1.946710000	-3.448879000	17.411330000

8	0.022058000	-2.367886000	17.455161000
1	-1.541258000	-4.165001000	16.554233000
8	-1.048630000	-4.969322000	15.748220000
8	-3.063344000	-5.822344000	15.335999000
6	-1.728783000	-5.964436000	15.292545000
8	-1.217136000	-6.997089000	14.830511000
6	-5.509806000	-7.950692000	13.883363000
6	-5.507387000	-6.874293000	12.956899000
6	-6.629963000	-8.823142000	13.849425000
6	-6.549527000	-6.680306000	12.053251000
6	-7.665877000	-8.623065000	12.933101000
6	-7.643752000	-7.555944000	12.028343000
1	-4.654539000	-6.191021000	12.972413000
1	-6.667765000	-9.644044000	14.561546000
1	-6.507702000	-5.836842000	11.357617000
1	-8.512777000	-9.316590000	12.931232000
1	-8.461735000	-7.407196000	11.319197000
1	-0.600223000	-6.097338000	18.898521000

^{3}IM1$_I$:

26	-1.049722000	-0.829348000	20.317509000
8	-5.983805000	-8.602456000	16.094679000
7	-0.436533000	-2.768118000	19.968776000
7	0.557280000	-0.563799000	21.625969000
7	-4.061342000	-7.793394000	15.108078000
7	-5.235341000	-9.389445000	14.014857000
7	-1.860829000	0.946457000	21.005976000
7	-2.867557000	-1.291673000	19.401522000
6	-1.223869000	1.876189000	21.787576000
6	-1.066793000	-3.705112000	19.184889000
6	-3.900314000	-0.407470000	19.216066000
6	1.593528000	-1.438738000	21.823161000
6	-3.140703000	-2.406231000	18.648131000
6	-2.051612000	3.065362000	21.900202000
1	2.555096000	-3.309003000	21.512566000
6	-3.054301000	1.496549000	20.610711000
6	-3.186302000	2.829857000	21.172043000
6	-3.991742000	0.870727000	19.783375000
6	-0.262396000	-4.917406000	19.151238000
6	0.733623000	-3.330297000	20.419953000
1	-4.890215000	1.444636000	19.543340000
1	0.391436000	2.542729000	22.998599000
6	0.851501000	-4.681445000	19.909499000
6	-2.314827000	-3.548212000	18.538817000
6	1.672202000	-2.716380000	21.260591000
6	-4.406175000	-2.215816000	17.953469000
6	0.844719000	0.573920000	22.332260000
6	-2.063298000	-5.054768000	16.527910000
6	-2.787516000	-4.665486000	17.671383000
6	0.025121000	1.707120000	22.397082000
6	-4.878342000	-0.983098000	18.313887000
6	2.583523000	-0.833727000	22.697896000
6	2.120253000	0.416174000	23.011128000
6	-3.697081000	-6.793039000	15.985967000
6	-5.188097000	-8.628843000	15.132941000
6	-3.971363000	-5.370019000	17.946694000
6	-2.501046000	-6.084160000	15.702949000
6	-4.425644000	-6.411609000	17.137180000
1	-3.399926000	-7.958491000	14.318109000
1	-4.393382000	-8.864562000	12.619907000
1	-1.789994000	3.957335000	22.469198000
1	-4.040885000	3.489629000	21.023470000
1	-0.528561000	-5.832207000	18.628063000
1	1.676504000	-5.361034000	20.121828000
1	-4.865969000	-2.930770000	17.276175000
1	-5.799125000	-0.496463000	17.992886000
1	3.507810000	-1.310493000	23.023711000
1	2.587564000	1.166484000	23.648738000
1	-4.552747000	-5.099272000	18.832202000
1	-1.937738000	-6.365638000	14.809984000
1	-5.337573000	-6.953337000	17.373931000
6	-0.246112000	-0.070338000	18.791180000
8	-0.390456000	-0.795695000	17.629198000
8	0.375063000	0.981764000	18.729913000
1	0.069140000	-0.277952000	16.940722000
8	-2.083077000	-7.916069000	10.821091000
8	-3.993852000	-8.608372000	11.724566000
6	-2.695119000	-8.184078000	11.868072000
8	-2.251658000	-8.102763000	13.056645000
6	-6.174231000	-10.374770000	13.806930000
6	-6.108723000	-11.067939000	12.562403000
6	-7.198604000	-10.792452000	14.706006000
6	-6.993203000	-12.093031000	12.239392000
6	-8.075985000	-11.826463000	14.369364000
6	-7.992199000	-12.490451000	13.140303000
1	-5.333056000	-10.763498000	11.856051000
1	-7.282381000	-10.280326000	15.660949000
1	-6.901850000	-12.592987000	11.269898000
1	-8.848046000	-12.118053000	15.089268000
1	-8.686610000	-13.295889000	12.888522000
1	-1.141625000	-4.523100000	16.277538000

^{3}RC2$_I$:

26	-1.054130000	-0.819180000	20.331725000
8	-5.925832000	-8.604690000	16.070034000
7	-0.444284000	-2.759157000	19.973517000
7	0.548177000	-0.568211000	21.645702000
7	-4.050926000	-7.731731000	15.046764000
7	-5.268782000	-9.344887000	13.985020000
7	-1.861794000	0.956865000	21.026063000
7	-2.863752000	-1.264318000	19.398494000
6	-1.225041000	1.878796000	21.816828000
6	-1.070387000	-3.684879000	19.174161000
6	-3.896111000	-0.378147000	19.218378000
6	1.581917000	-1.446690000	21.840190000
6	-3.138270000	-2.374146000	18.638993000
6	-2.049940000	3.069365000	21.935883000
1	2.541959000	-3.316393000	21.520300000
6	-3.052282000	1.512975000	20.631183000
6	-3.182884000	2.842281000	21.202383000
6	-3.987959000	0.895344000	19.795295000
6	-0.266027000	-4.897174000	19.126710000
6	0.723815000	-3.327410000	20.422097000
1	-4.885196000	1.472029000	19.557452000
1	0.389602000	2.530858000	23.036259000
6	0.843567000	-4.672038000	19.894311000
6	-2.314111000	-3.515691000	18.525196000
6	1.660103000	-2.721446000	21.270297000
6	-4.404201000	-2.179437000	17.946012000
6	0.837476000	0.565210000	22.358220000
6	-2.071133000	-4.982179000	16.486693000
6	-2.785754000	-4.624409000	17.644254000
6	0.021587000	1.700711000	22.428272000
6	-4.874687000	-0.948185000	18.312977000
6	2.571803000	-0.848813000	22.719905000
6	2.111281000	0.400565000	23.038708000
6	-3.683950000	-6.729165000	15.944896000
6	-5.148191000	-8.564040000	15.115361000
6	-3.954833000	-5.347605000	17.926829000
6	-2.505489000	-6.006539000	15.651730000
6	-4.405252000	-6.382808000	17.105406000
1	-3.375283000	-7.877613000	14.265975000
1	-4.651945000	-9.124960000	13.174265000
1	-1.787940000	3.956257000	22.512545000

1	-4.035151000	3.505479000	21.055922000
1	-0.528328000	-5.804483000	18.588485000
1	1.667794000	-5.354420000	20.100468000
1	-4.866293000	-2.890728000	17.266227000
1	-5.795543000	-0.459398000	17.995612000
1	3.494171000	-1.329797000	23.044871000
1	2.579308000	1.146462000	23.680905000
1	-4.527901000	-5.099097000	18.823537000
1	-1.945084000	-6.265023000	14.751050000
1	-5.306825000	-6.936196000	17.352347000
6	-0.227995000	-0.053495000	18.821231000
8	-0.380848000	-0.759096000	17.648602000
8	0.414836000	0.986082000	18.780388000
1	0.093901000	-0.242344000	16.969749000
8	-1.719039000	-8.323581000	10.906036000
8	-3.733820000	-8.865505000	11.725718000
6	-2.592687000	-8.401258000	11.966596000
8	-2.142233000	-7.994889000	13.058321000
6	-6.190188000	-10.365576000	13.745454000
6	-6.123343000	-11.000400000	12.483892000
6	-7.160621000	-10.804547000	14.669947000
6	-6.997970000	-12.035749000	12.160966000
6	-8.028054000	-11.846515000	14.329043000
6	-7.960337000	-12.471014000	13.080481000
1	-5.370315000	-10.660741000	11.769888000
1	-7.223627000	-10.318723000	15.639515000
1	-6.925111000	-12.509043000	11.178045000
1	-8.773205000	-12.172010000	15.060333000
1	-8.645382000	-13.282998000	12.826237000
1	-1.160151000	-4.434603000	16.233585000
1	-2.194297000	-8.673545000	10.137229000

^{3}TS2$_{I}$:

26	-1.598985000	-1.816660000	19.538320000
8	-5.366266000	-9.304613000	15.808684000
7	-0.516170000	-3.437946000	20.344588000
7	0.157854000	-0.652027000	19.632085000
7	-3.173442000	-8.596784000	16.014456000
7	-4.347686000	-8.057534000	14.137512000
7	-2.645715000	0.001878000	19.525121000
7	-3.331932000	-2.790537000	20.242149000
6	-2.159998000	1.207218000	19.097711000
6	-0.998730000	-4.669011000	20.591801000
6	-4.550444000	-2.172906000	20.520552000
6	1.422719000	-1.110126000	19.924190000
6	-3.456086000	-4.095364000	20.558401000
6	-3.245028000	2.160801000	18.998271000
1	2.764915000	-2.568499000	20.681963000
6	-3.996691000	0.157579000	19.737400000
6	-4.382388000	1.510299000	19.410417000
6	-4.848267000	-0.836426000	20.240386000
6	0.038658000	-5.507191000	21.135583000
6	0.839339000	-3.444422000	20.669530000
1	-5.878832000	-0.527366000	20.434553000
1	-0.548182000	2.496177000	18.548825000
6	1.186561000	-4.738839000	21.181351000
6	-2.378901000	-5.055690000	20.277507000
6	1.714747000	-2.381515000	20.442109000
6	-4.762171000	-4.348208000	21.103067000
6	0.284944000	0.635185000	19.188708000
6	-1.545164000	-6.886840000	18.803614000
6	-2.624438000	-6.207230000	19.454703000
6	-0.796761000	1.488793000	18.889161000
6	-5.446812000	-3.145210000	21.069540000
6	2.386763000	-0.075311000	19.641168000
6	1.682611000	1.007259000	19.169164000
6	-3.059734000	-7.997692000	17.256438000
6	-4.402333000	-8.703559000	15.338937000
6	-3.936458000	-6.608629000	19.045977000
6	-1.755305000	-7.740363000	17.743941000
6	-4.151224000	-7.484994000	17.996792000
1	-2.352782000	-8.330358000	15.398659000
1	-3.634278000	-7.246525000	14.126353000
1	-3.140845000	3.197873000	18.681895000
1	-5.392236000	1.910639000	19.493356000
1	-0.085545000	-6.541750000	21.449443000
1	2.175751000	-5.032955000	21.530699000
1	-5.118219000	-5.302400000	21.485194000
1	-6.467027000	-2.947719000	21.396827000
1	3.462603000	-0.164063000	19.788368000
1	2.068897000	1.977485000	18.859497000
1	-4.805950000	-6.164500000	19.525749000
1	-0.907841000	-8.131859000	17.181615000
1	-5.168536000	-7.724930000	17.687821000
6	-1.471230000	-2.137608000	17.475086000
8	-2.217834000	-3.951926000	17.050597000
8	-1.157719000	-1.688322000	16.459493000
1	-2.240315000	-4.480074000	17.862203000
8	-0.925291000	-5.364651000	15.533483000
8	-2.781238000	-5.963982000	14.433405000
6	-1.612146000	-6.261361000	14.880888000
8	-1.124701000	-7.438343000	14.722807000
6	-5.316406000	-8.047552000	13.135416000
6	-5.138883000	-7.116580000	12.088567000
6	-6.428239000	-8.912950000	13.090709000
6	-6.043453000	-7.054468000	11.030476000
6	-7.323940000	-8.841666000	12.020259000
6	-7.144827000	-7.918418000	10.985271000
1	-4.277803000	-6.445680000	12.132477000
1	-6.581509000	-9.621273000	13.901486000
1	-5.886055000	-6.324307000	10.232148000
1	-8.180252000	-9.521478000	12.000133000
1	-7.853577000	-7.870529000	10.155420000
1	-0.519294000	-6.645043000	19.075875000
1	-1.643664000	-4.573867000	16.299608000

^{3}IM2$_{I}$:

26	-0.991859000	-0.841452000	20.345080000
8	-4.519213000	-9.491373000	16.420040000
7	-0.803050000	-2.833437000	20.574439000
7	-0.080624000	-0.542337000	22.121733000
7	-3.694031000	-7.811068000	15.067946000
7	-5.155855000	-9.309158000	14.196164000
7	-1.281653000	1.145193000	20.173948000
7	-1.999454000	-1.151674000	18.632718000
6	-0.836685000	2.127381000	21.027900000
6	-1.185596000	-3.818564000	19.688152000
6	-2.565154000	-0.181957000	17.833447000
6	0.443041000	-1.504963000	22.957620000
6	-2.271987000	-2.356728000	18.018342000
6	-1.202595000	3.433684000	20.526986000
1	0.888464000	-3.521485000	23.457213000
6	-1.921158000	1.803290000	19.146981000
6	-1.879200000	3.232215000	19.358078000
6	-2.525318000	1.189883000	18.059031000
6	-0.751263000	-5.110934000	20.173129000
6	-0.154477000	-3.479519000	21.605780000
1	-3.008387000	1.832357000	17.321129000
1	0.143662000	2.791154000	22.796321000
6	-0.122432000	-4.899514000	21.367157000
6	-1.882503000	-3.624979000	18.484704000
6	0.418357000	-2.871264000	22.717850000
6	-3.048630000	-2.133816000	16.811691000
6	0.194129000	0.665333000	22.721859000

6	-1.807726000	-4.933909000	16.338104000
6	-2.255262000	-4.808782000	17.664951000
6	-0.144991000	1.914248000	22.214790000
6	-3.219507000	-0.783851000	16.699792000
6	1.050126000	-0.890917000	24.115422000
6	0.895885000	0.458370000	23.968310000
6	-3.181046000	-6.921200000	15.996720000
6	-4.469220000	-8.922188000	15.324076000
6	-3.118749000	-5.803485000	18.155602000
6	-2.254750000	-5.963639000	15.521981000
6	-3.570802000	-6.849290000	17.351034000
1	-3.615186000	-7.458671000	14.048910000
1	-5.284547000	-8.549266000	13.436730000
1	-0.965956000	4.374160000	21.022379000
1	-2.316391000	3.970567000	18.687804000
1	-0.905711000	-6.053541000	19.656065000
1	0.337319000	-5.630211000	22.030505000
1	-3.417017000	-2.895440000	16.127592000
1	-3.747513000	-0.233371000	15.922700000
1	1.529468000	-1.437179000	24.926224000
1	1.221870000	1.256393000	24.633747000
1	-3.477629000	-5.735367000	19.185523000
1	-1.937350000	-6.029755000	14.480438000
1	-4.255491000	-7.596204000	17.747224000
6	1.275850000	-0.997153000	19.512704000
8	-3.977832000	-3.719480000	13.854975000
8	2.256229000	-1.497582000	19.784084000
1	-3.241359000	-4.322872000	14.020732000
8	-5.225730000	-5.241856000	12.106664000
8	-5.678147000	-7.399746000	12.468101000
6	-4.834065000	-6.419003000	12.428904000
8	-3.591620000	-6.636741000	12.747436000
6	-5.924248000	-10.453264000	14.025476000
6	-6.648786000	-10.556587000	12.813504000
6	-6.013770000	-11.515539000	14.951770000
6	-7.429850000	-11.677379000	12.541728000
6	-6.799914000	-12.634070000	14.660693000
6	-7.513556000	-12.730614000	13.462020000
1	-6.579964000	-9.729262000	12.103583000
1	-5.472190000	-11.439979000	15.891343000
1	-7.980282000	-11.729948000	11.598149000
1	-6.854688000	-13.445616000	15.392143000
1	-8.126132000	-13.609449000	13.247790000
1	-1.113232000	-4.192679000	15.936171000
1	-4.482480000	-4.242359000	13.155925000

^{5}RC1$_I$:

26	-0.954480000	-0.686456000	20.339910000
8	-5.959113000	-8.485515000	16.171535000
7	-0.394394000	-2.686197000	20.013838000
7	0.584604000	-0.512178000	21.752144000
7	-4.125272000	-7.595884000	15.090594000
7	-5.360230000	-9.220325000	14.066676000
7	-1.805139000	1.069538000	21.141692000
7	-2.801148000	-1.138500000	19.451736000
6	-1.175066000	1.970140000	21.980044000
6	-1.032409000	-3.603677000	19.196196000
6	-3.824430000	-0.238612000	19.285780000
6	1.614475000	-1.400129000	21.923363000
6	-3.084660000	-2.248744000	18.671300000
6	-1.990128000	3.137666000	22.127480000
1	2.584022000	-3.260905000	21.591125000
6	-2.990273000	1.633613000	20.753423000
6	-3.130373000	2.928417000	21.361804000
6	-3.925827000	1.021355000	19.878331000
6	-0.254907000	-4.807321000	19.142754000
6	0.759804000	-3.271125000	20.466865000
1	-4.823347000	1.599584000	19.643581000
1	0.451720000	2.554106000	23.237964000
6	0.866933000	-4.598575000	19.933237000
6	-2.287465000	-3.393729000	18.544523000
6	1.703263000	-2.663977000	21.340535000
6	-4.348625000	-2.024856000	17.982816000
6	0.876188000	0.616297000	22.498010000
6	-2.088823000	-4.869185000	16.500851000
6	-2.782859000	-4.494886000	17.666193000
6	0.077225000	1.747771000	22.601178000
6	-4.806461000	-0.795768000	18.367952000
6	2.604467000	-0.818035000	22.821728000
6	2.149331000	0.423893000	23.171655000
6	-3.732669000	-6.595098000	15.982755000
6	-5.211355000	-8.437333000	15.193289000
6	-3.956638000	-5.204928000	17.963998000
6	-2.548249000	-5.889314000	15.673392000
6	-4.433894000	-6.235607000	17.150293000
1	-3.470089000	-7.743400000	14.294320000
1	-4.774503000	-8.992063000	13.235063000
1	-1.742213000	4.006017000	22.738783000
1	-3.981358000	3.597650000	21.230280000
1	-0.519760000	-5.707560000	18.592936000
1	1.680997000	-5.295463000	20.135377000
1	-4.820415000	-2.721862000	17.294180000
1	-5.723631000	-0.295841000	18.055023000
1	3.526208000	-1.308064000	23.137029000
1	2.621683000	1.150910000	23.833039000
1	-4.514164000	-4.946108000	18.867659000
1	-2.002987000	-6.156959000	14.765900000
1	-5.339760000	-6.775689000	17.411198000
6	0.029652000	0.164238000	18.710713000
8	-0.722556000	0.924070000	18.102601000
8	1.194206000	-0.213115000	18.599357000
1	-1.094119000	-7.889601000	11.161014000
8	-1.939186000	-8.255161000	10.858264000
8	-3.910475000	-8.732074000	11.756243000
6	-2.760145000	-8.291294000	11.962810000
8	-2.270735000	-7.883383000	13.044078000
6	-6.269244000	-10.258996000	13.862087000
6	-6.233168000	-10.898531000	12.601430000
6	-7.198740000	-10.713089000	14.820972000
6	-7.097237000	-11.952805000	12.313183000
6	-8.055829000	-11.774118000	14.514561000
6	-8.018372000	-12.403287000	13.267038000
1	-5.511705000	-10.547265000	11.860891000
1	-7.238256000	-10.223629000	15.789950000
1	-7.048526000	-12.429287000	11.330275000
1	-8.768532000	-12.111124000	15.272540000
1	-8.694878000	-13.230356000	13.039953000
1	-1.171052000	-4.338937000	16.235791000

^{5}IM1$_I$:

26	-0.944897000	-0.726370000	20.138086000
8	-6.088992000	-8.459490000	15.999923000
7	-0.371233000	-2.737449000	19.815786000
7	0.555967000	-0.575687000	21.621929000
7	-4.075418000	-7.801517000	15.084691000
7	-5.243377000	-9.434979000	14.040869000
7	-1.876460000	0.925481000	21.093722000
7	-2.840657000	-1.289290000	19.382227000
6	-1.296667000	1.792759000	21.978109000
6	-1.018622000	-3.681615000	19.061639000
6	-3.878832000	-0.405582000	19.238532000
6	1.609669000	-1.440079000	21.756890000
6	-3.103295000	-2.372878000	18.582651000
6	-2.188199000	2.920525000	22.208831000

1	2.605387000	-3.277977000	21.377248000
6	-3.098702000	1.440646000	20.755535000
6	-3.304625000	2.703493000	21.450306000
6	-4.007039000	0.838236000	19.881816000
6	-0.226155000	-4.904738000	19.046407000
6	0.788306000	-3.301506000	20.279342000
1	-4.927705000	1.390229000	19.678615000
1	0.293278000	2.397917000	23.257703000
6	0.895856000	-4.663480000	19.789036000
6	-2.276286000	-3.517575000	18.433260000
6	1.715789000	-2.691820000	21.135112000
6	-4.364154000	-2.158938000	17.895165000
6	0.807944000	0.509118000	22.417422000
6	-2.022645000	-5.088880000	16.471645000
6	-2.769769000	-4.628213000	17.574741000
6	-0.044652000	1.612865000	22.577447000
6	-4.847327000	-0.945432000	18.310463000
6	2.579579000	-0.879258000	22.677712000
6	2.081074000	0.330265000	23.087772000
6	-3.707537000	-6.778668000	15.931883000
6	-5.236458000	-8.591010000	15.097471000
6	-3.993544000	-5.273541000	17.828354000
6	-2.473628000	-6.128445000	15.668323000
6	-4.462038000	-6.322924000	17.039344000
1	-3.375283000	-8.047032000	14.350220000
1	-4.271776000	-9.081661000	12.671054000
1	-1.978116000	3.758933000	22.872465000
1	-4.195565000	3.325415000	21.368991000
1	-0.506515000	-5.829550000	18.549192000
1	1.712303000	-5.348600000	20.015002000
1	-4.816474000	-2.841810000	17.181216000
1	-5.772253000	-0.453627000	18.011087000
1	3.512018000	-1.358398000	22.974491000
1	2.526523000	1.038931000	23.785430000
1	-4.591432000	-4.953528000	18.685570000
1	-1.889361000	-6.467651000	14.809657000
1	-5.403337000	-6.818480000	17.261577000
6	-0.062746000	0.286361000	18.491745000
8	-0.515489000	-0.072684000	17.229095000
8	0.787331000	1.174937000	18.510755000
1	-0.022195000	0.500771000	16.603885000
8	-1.803726000	-8.372428000	10.976550000
8	-3.797981000	-8.927991000	11.789513000
6	-2.501446000	-8.522017000	11.992840000
8	-2.146798000	-8.334997000	13.199408000
6	-6.217034000	-10.388131000	13.839606000
6	-6.081000000	-11.206036000	12.679607000
6	-7.343647000	-10.657047000	14.670381000
6	-6.995101000	-12.209461000	12.371214000
6	-8.250974000	-11.669907000	14.349150000
6	-8.096486000	-12.458366000	13.203388000
1	-5.226629000	-11.016538000	12.025895000
1	-7.479283000	-10.048987000	15.560877000
1	-6.846647000	-12.809463000	11.467815000
1	-9.103333000	-11.844601000	15.014210000
1	-8.814695000	-13.246141000	12.962712000
1	-1.071873000	-4.605062000	16.234480000

^{5}RC2$_{I}$:

26	-0.933437000	-0.720086000	20.156415000
8	-5.933872000	-8.572469000	16.072179000
7	-0.408229000	-2.747489000	19.860609000
7	0.505694000	-0.617588000	21.701747000
7	-4.079970000	-7.712263000	14.999849000
7	-5.296684000	-9.364597000	14.000159000
7	-1.850868000	0.964174000	21.065460000
7	-2.812555000	-1.223140000	19.322504000
6	-1.283697000	1.811773000	21.977070000
6	-1.045103000	-3.666151000	19.068029000
6	-3.829077000	-0.319683000	19.151795000
6	1.532695000	-1.507359000	21.871023000
6	-3.084734000	-2.309399000	18.531099000
6	-2.153203000	2.962116000	22.178417000
1	2.502456000	-3.364384000	21.513164000
6	-3.046485000	1.510879000	20.685828000
6	-3.245249000	2.776832000	21.377401000
6	-3.943115000	0.930219000	19.784718000
6	-0.271459000	-4.902016000	19.052015000
6	0.724902000	-3.340219000	20.352281000
1	-4.844341000	1.503663000	19.555678000
1	0.269919000	2.367274000	23.321391000
6	0.826619000	-4.695133000	19.838639000
6	-2.278098000	-3.468965000	18.406445000
6	1.633321000	-2.758855000	21.245948000
6	-4.331593000	-2.080177000	17.822927000
6	0.760065000	0.462871000	22.502936000
6	-2.046027000	-4.982302000	16.399434000
6	-2.772825000	-4.570045000	17.531358000
6	-0.064223000	1.592549000	22.627561000
6	-4.794199000	-0.850859000	18.213979000
6	2.484552000	-0.970966000	22.824295000
6	2.003781000	0.251560000	23.216947000
6	-3.701865000	-6.693809000	15.873325000
6	-5.169718000	-8.551631000	15.106353000
6	-3.972565000	-5.245599000	17.806890000
6	-2.496513000	-6.014990000	15.583869000
6	-4.437456000	-6.289645000	17.006235000
1	-3.405267000	-7.886168000	14.222972000
1	-4.694802000	-9.158608000	13.174293000
1	-1.947310000	3.791661000	22.854326000
1	-4.116596000	3.421400000	21.266069000
1	-0.547709000	-5.810111000	18.522227000
1	1.624716000	-5.397515000	20.076674000
1	-4.789547000	-2.766177000	17.115136000
1	-5.704775000	-0.344413000	17.895748000
1	3.394236000	-1.473171000	23.151832000
1	2.443987000	0.949886000	23.928169000
1	-4.555005000	-4.955320000	18.684649000
1	-1.926752000	-6.315609000	14.702354000
1	-5.359642000	-6.808722000	17.251551000
6	0.061611000	0.237422000	18.542488000
8	-0.369421000	-0.102265000	17.267035000
8	0.967603000	1.067747000	18.586511000
1	0.182446000	0.431530000	16.655773000
8	-1.782907000	-8.412001000	10.867368000
8	-3.796456000	-8.920203000	11.711414000
6	-2.648060000	-8.465844000	11.935844000
8	-2.183783000	-8.049887000	13.018301000
6	-6.199145000	-10.411977000	13.808413000
6	-6.137559000	-11.086371000	12.567429000
6	-7.144158000	-10.841791000	14.762905000
6	-6.992750000	-12.151623000	12.293360000
6	-7.992015000	-11.914348000	14.471232000
6	-7.929588000	-12.578181000	13.242845000
1	-5.403325000	-10.754311000	11.830534000
1	-7.202955000	-10.325475000	15.716871000
1	-6.924423000	-12.655681000	11.325541000
1	-8.717237000	-12.232610000	15.225373000
1	-8.599088000	-13.413920000	13.027097000
1	-1.112180000	-4.472705000	16.150740000
1	-2.268403000	-8.766168000	10.106916000

^{5}TS2$_{I}$:

26	-1.430324000	-1.684886000	19.495317000

8	-4.669183000	-10.244396000	15.704131000
7	-0.569847000	-3.379255000	20.397333000
7	0.067428000	-0.521704000	20.404065000
7	-2.681573000	-9.042717000	15.734979000
7	-4.519400000	-8.203097000	14.629661000
7	-2.525107000	0.097714000	19.238985000
7	-3.200495000	-2.784927000	19.308667000
6	-2.049963000	1.374953000	19.350436000
6	-1.012582000	-4.659876000	20.225322000
6	-4.334024000	-2.275743000	18.725853000
6	1.189308000	-0.997987000	21.038128000
6	-3.209818000	-4.150141000	19.120379000
6	-3.086236000	2.316136000	18.952909000
1	2.379110000	-2.548518000	21.869527000
6	-3.821471000	0.179501000	18.804557000
6	-4.184631000	1.575524000	18.617377000
6	-4.648433000	-0.916162000	18.555831000
6	-0.071286000	-5.580324000	20.842392000
6	0.622391000	-3.439029000	21.066623000
1	-5.632869000	-0.700593000	18.134648000
1	-0.529454000	2.783645000	19.834459000
6	0.935651000	-4.821584000	21.374736000
6	-2.180103000	-5.043857000	19.524550000
6	1.440309000	-2.338009000	21.352606000
6	-4.412209000	-4.509470000	18.390531000
6	0.192359000	0.841402000	20.320737000
6	-1.124658000	-6.725947000	18.077136000
6	-2.197399000	-6.405906000	18.935875000
6	-0.775980000	1.720080000	19.814033000
6	-5.122503000	-3.354990000	18.185762000
6	2.069053000	0.108145000	21.354906000
6	1.454521000	1.246933000	20.905345000
6	-2.458639000	-8.401696000	16.957176000
6	-4.044457000	-9.245122000	15.363556000
6	-3.306258000	-7.269861000	18.930106000
6	-1.233197000	-7.729092000	17.129666000
6	-3.439787000	-8.265146000	17.961833000
1	-2.174870000	-8.477688000	15.003535000
1	-3.930636000	-7.300475000	14.667071000
1	-2.976687000	3.399959000	18.947109000
1	-5.158909000	1.928429000	18.281391000
1	-0.185837000	-6.661338000	20.876452000
1	1.813229000	-5.158457000	21.925139000
1	-4.671346000	-5.503223000	18.043249000
1	-6.073549000	-3.240198000	17.667569000
1	3.028121000	0.017051000	21.863366000
1	1.808895000	2.274731000	20.973794000
1	-4.118589000	-7.109502000	19.642479000
1	-0.451340000	-7.862037000	16.383063000
1	-4.350464000	-8.864486000	17.916127000
6	-0.543666000	-2.070425000	17.561324000
8	-1.781693000	-3.262898000	16.413334000
8	0.400141000	-1.919227000	16.918899000
1	-2.478266000	-3.599256000	16.992068000
8	-1.078308000	-5.112937000	15.000606000
8	-3.163090000	-5.941599000	14.921289000
6	-1.903538000	-6.082018000	14.692986000
8	-1.430201000	-7.153349000	14.182575000
6	-5.778098000	-8.076555000	14.036905000
6	-6.068342000	-6.834581000	13.430880000
6	-6.749886000	-9.096037000	13.996968000
6	-7.293082000	-6.621047000	12.801719000
6	-7.971886000	-8.867177000	13.357430000
6	-8.256159000	-7.637086000	12.756916000
1	-5.310228000	-6.049534000	13.475660000
1	-6.536325000	-10.048775000	14.475216000
1	-7.497564000	-5.651174000	12.340407000
1	-8.715270000	-9.668786000	13.333305000
1	-9.215674000	-7.469930000	12.262167000
1	-0.239108000	-6.088945000	18.073293000
1	-1.485441000	-4.172869000	15.721539000

[5]IM2$_I$:

26	-0.768009000	-0.824150000	20.253658000
8	-4.540059000	-9.503621000	16.422830000
7	-0.795522000	-2.888333000	20.576016000
7	-0.036206000	-0.524874000	22.200645000
7	-3.733760000	-7.817451000	15.065996000
7	-5.185326000	-9.327799000	14.200626000
7	-1.191939000	1.212115000	20.186852000
7	-1.927187000	-1.162171000	18.553807000
6	-0.804497000	2.173021000	21.082998000
6	-1.191184000	-3.858205000	19.688466000
6	-2.464837000	-0.165526000	17.779259000
6	0.446048000	-1.512964000	23.020424000
6	-2.239258000	-2.362247000	17.967527000
6	-1.191892000	3.478032000	20.583257000
1	0.851257000	-3.542402000	23.488334000
6	-1.817294000	1.846159000	19.142718000
6	-1.818024000	3.275865000	19.382102000
6	-2.393275000	1.210851000	18.037276000
6	-0.775625000	-5.154078000	20.193127000
6	-0.158288000	-3.512255000	21.621536000
1	-2.867159000	1.861716000	17.299362000
1	0.092913000	2.799221000	22.909207000
6	-0.147299000	-4.938677000	21.391128000
6	-1.876725000	-3.637402000	18.468757000
6	0.402809000	-2.882430000	22.742725000
6	-3.025427000	-2.113421000	16.765146000
6	0.191035000	0.674160000	22.820227000
6	-1.846292000	-4.933224000	16.315080000
6	-2.266969000	-4.817075000	17.652513000
6	-0.154344000	1.927752000	22.299677000
6	-3.151489000	-0.755610000	16.650269000
6	1.002894000	-0.913732000	24.219260000
6	0.843379000	0.439919000	24.096176000
6	-3.213563000	-6.926801000	15.989522000
6	-4.498246000	-8.934780000	15.326377000
6	-3.116638000	-5.819737000	18.152952000
6	-2.303273000	-5.960245000	15.501920000
6	-3.578589000	-6.863460000	17.351245000
1	-3.659068000	-7.469622000	14.045436000
1	-5.325375000	-8.568026000	13.443017000
1	-1.010236000	4.421680000	21.096117000
1	-2.252148000	4.020198000	18.715926000
1	-0.936556000	-6.104413000	19.691688000
1	0.293452000	-5.677163000	22.059319000
1	-3.433255000	-2.859856000	16.086045000
1	-3.677018000	-0.197985000	15.876130000
1	1.448384000	-1.467025000	25.044928000
1	1.135183000	1.218403000	24.799979000
1	-3.458302000	-5.758758000	19.189045000
1	-2.005588000	-6.020206000	14.454176000
1	-4.251926000	-7.616052000	17.756055000
6	1.194491000	-0.873555000	19.427276000
8	-4.021794000	-3.712892000	13.821987000
8	2.285808000	-1.026248000	19.168371000
1	-3.276895000	-4.304391000	13.991797000
8	-5.280068000	-5.262486000	12.106811000
8	-5.730443000	-7.419062000	12.478136000
6	-4.886105000	-6.438896000	12.428917000
8	-3.641160000	-6.656704000	12.737155000
6	-5.936108000	-10.483119000	14.028932000
6	-6.669030000	-10.591208000	12.822449000

6	-5.998088000	-11.553327000	14.948304000
6	-7.431765000	-11.724276000	12.549373000
6	-6.766084000	-12.684055000	14.656027000
6	-7.488155000	-12.785295000	13.462771000
1	-6.620450000	-9.758339000	12.117328000
1	-5.449435000	-11.474950000	15.883560000
1	-7.989058000	-11.780497000	11.610042000
1	-6.799401000	-13.501697000	15.381950000
1	-8.086282000	-13.673754000	13.247433000
1	-1.161138000	-4.188166000	15.904588000
1	-4.527941000	-4.251830000	13.135887000

[Fe^{II}(*ortho*-amide-TPP)(HCO_3)]$^{3-}$

Solvent = water::

^{3}RC1$_{II}$:

26	-0.966289000	-0.735891000	19.984731000
8	-0.653506000	-6.521881000	15.740515000
7	-0.347750000	-2.559194000	19.420159000
7	0.838891000	-0.391005000	20.880681000
6	-2.032157000	-4.526525000	15.794709000
7	-0.194159000	-4.826901000	14.261712000
7	-1.643470000	0.990898000	20.774477000
7	-2.880479000	-1.310086000	19.589349000
6	-0.913165000	1.958812000	21.436551000
6	-1.093556000	-3.585119000	18.861644000
6	-3.991621000	-0.486665000	19.638384000
6	1.977077000	-1.160132000	20.737677000
6	-3.298891000	-2.472217000	18.973280000
6	-1.754771000	3.111604000	21.728553000
1	2.993724000	-2.908743000	20.035464000
6	-2.930310000	1.496467000	20.669125000
6	-2.996485000	2.827300000	21.251208000
6	-4.011478000	0.828909000	20.145488000
6	-0.227367000	-4.716174000	18.546285000
6	0.957620000	-3.032617000	19.481517000
1	-4.970133000	1.352672000	20.127066000
1	0.856832000	2.706675000	22.336463000
6	1.033847000	-4.365230000	18.911848000
6	-2.465014000	-3.584001000	18.648042000
6	2.027834000	-2.399253000	20.068826000
6	-4.688275000	-2.388540000	18.651841000
6	1.240412000	0.750563000	21.540047000
6	-2.959259000	-5.229451000	16.750046000
6	-3.137343000	-4.794100000	18.082738000
6	0.410695000	1.860421000	21.809539000
6	-5.121320000	-1.137906000	19.075205000
6	3.095252000	-0.520050000	21.343349000
6	2.629933000	0.685220000	21.855723000
6	-4.553873000	-7.045023000	17.125648000
6	-0.896542000	-5.411053000	15.269272000
6	-4.027413000	-5.509220000	18.909823000
6	-3.676847000	-6.349649000	16.295806000
6	-4.727256000	-6.622438000	18.449056000
1	-1.559697000	-3.655954000	16.270545000
1	-0.481574000	-3.848751000	13.988687000
1	-1.413694000	4.008389000	22.245858000
1	-3.897916000	3.438777000	21.292754000
1	-0.556971000	-5.642805000	18.083811000
1	1.948821000	-4.952788000	18.835741000
1	-5.267438000	-3.164853000	18.158300000
1	-6.119013000	-0.705817000	18.996832000
1	4.106407000	-0.926028000	21.376218000
1	3.188300000	1.458786000	22.383010000
1	-5.098663000	-7.912895000	16.745334000
1	-4.165707000	-5.168146000	19.938356000
1	-3.536432000	-6.679739000	15.263323000
1	-5.405545000	-7.159118000	19.117452000
6	-0.587053000	0.212055000	18.264756000
8	-1.559083000	0.207312000	17.481702000
8	0.544628000	0.681140000	18.167050000
1	-1.484369000	-0.340089000	15.893943000
8	-1.646730000	-0.691321000	14.977370000
8	-0.946965000	-2.306729000	13.632369000
6	-0.748200000	-1.689414000	14.724091000
8	0.145211000	-1.919641000	15.560817000
6	0.971346000	-5.286114000	13.641846000
6	1.594232000	-4.405401000	12.730431000
6	1.550920000	-6.551051000	13.866900000
6	2.759536000	-4.779540000	12.064741000
6	2.721232000	-6.910193000	13.191402000
6	3.334966000	-6.036633000	12.289175000
1	1.140937000	-3.425192000	12.569368000
1	1.076480000	-7.229975000	14.570007000
1	3.224943000	-4.080769000	11.364458000
1	3.158111000	-7.895207000	13.377735000
1	4.248999000	-6.329441000	11.767413000
1	-2.585524000	-4.130212000	14.927931000

^{3}IM1$_{II}$:

26	-1.016630000	-0.790243000	20.033203000
8	-0.609459000	-6.462769000	15.750004000
7	-0.407748000	-2.647146000	19.590812000
7	0.807514000	-0.381036000	20.825446000
6	-1.954517000	-4.451942000	15.865212000
7	-0.166707000	-4.743223000	14.281099000
7	-1.678359000	0.996771000	20.720905000
7	-2.919844000	-1.349610000	19.675773000
6	-0.909556000	2.029792000	21.215401000
6	-1.150496000	-3.658253000	19.006371000
6	-4.045375000	-0.539722000	19.748557000
6	1.929515000	-1.194094000	20.791994000
6	-3.336695000	-2.514223000	19.058376000
6	-1.739402000	3.198184000	21.464745000
1	2.912274000	-3.028209000	20.295079000
6	-2.974563000	1.485611000	20.662825000
6	-3.014082000	2.859080000	21.128847000
6	-4.071901000	0.781798000	20.229615000
6	-0.300764000	-4.810875000	18.740500000
6	0.883402000	-3.148627000	19.710422000
1	-5.037395000	1.292040000	20.229953000
1	0.918046000	2.870495000	21.890686000
6	0.948053000	-4.493058000	19.175440000
6	-2.509526000	-3.625625000	18.731495000
6	1.957563000	-2.498681000	20.269815000
6	-4.732457000	-2.442480000	18.762029000
6	1.243521000	0.829156000	21.323753000
6	-2.924931000	-5.186122000	16.753722000
6	-3.163684000	-4.809877000	18.095031000
6	0.440250000	1.973742000	21.491484000
6	-5.175485000	-1.205615000	19.207063000
6	3.063621000	-0.505749000	21.304866000
6	2.633181000	0.770674000	21.639335000
6	-4.538325000	-7.011690000	16.987268000
6	-0.843402000	-5.339105000	15.291617000
6	-4.082893000	-5.558864000	18.856675000
6	-3.627145000	-6.283701000	16.224760000
6	-4.764272000	-6.649579000	18.320381000
1	-1.444453000	-3.635119000	16.394358000
1	-0.397945000	-3.672502000	14.074777000
1	-1.368842000	4.143079000	21.861501000
1	-3.917975000	3.465059000	21.186348000
1	-0.631155000	-5.725289000	18.254401000
1	1.850872000	-5.102796000	19.149182000

1	-5.310244000	-3.222488000	18.273834000
1	-6.179616000	-0.786155000	19.151359000
1	4.063616000	-0.929986000	21.391349000
1	3.211734000	1.593899000	22.057782000
1	-5.067938000	-7.860533000	16.547073000
1	-4.260127000	-5.263579000	19.893421000
1	-3.444203000	-6.571941000	15.186576000
1	-5.467865000	-7.214715000	18.937083000
6	-0.660692000	0.013931000	18.311909000
8	-1.561465000	-0.311141000	17.414589000
8	0.314441000	0.709624000	18.102847000
1	-1.231574000	-0.262209000	16.471839000
8	-0.969158000	-0.430852000	15.042067000
8	-0.686675000	-2.267733000	13.803200000
6	-0.396556000	-1.590568000	14.889091000
8	0.397775000	-2.062610000	15.755433000
6	0.957318000	-5.230820000	13.617362000
6	1.584105000	-4.349038000	12.704961000
6	1.501238000	-6.523715000	13.781127000
6	2.708797000	-4.746312000	11.985761000
6	2.630731000	-6.906477000	13.051003000
6	3.244765000	-6.030546000	12.150922000
1	1.157526000	-3.349491000	12.595253000
1	1.028829000	-7.204653000	14.483905000
1	3.175037000	-4.044838000	11.288075000
1	3.036221000	-7.912573000	13.192482000
1	4.127044000	-6.341565000	11.586395000
1	-2.477006000	-3.969449000	15.023753000

^{3}RC2$_{II}$:

26	0.228890000	1.193134000	1.415693000
8	-0.140093000	-4.061241000	-2.684923000
7	0.617424000	-0.737822000	0.990910000
7	2.095493000	1.385660000	2.247380000
6	-1.914679000	-2.455618000	-2.479136000
7	0.416430000	-1.828434000	-2.715056000
7	-0.264509000	2.974340000	2.209908000
7	-1.747006000	0.798458000	1.065572000
6	0.574622000	3.884584000	2.812406000
6	-0.260789000	-1.725099000	0.584612000
6	-2.763640000	1.722247000	1.124128000
6	3.134597000	0.511042000	2.080427000
6	-2.310340000	-0.362193000	0.590049000
6	-0.139905000	5.113403000	3.107418000
1	3.930971000	-1.377669000	1.495321000
6	-1.488070000	3.595196000	2.117854000
6	-1.419563000	4.934252000	2.672822000
6	-2.644189000	3.029155000	1.596781000
6	0.447165000	-2.980044000	0.396430000
6	1.852880000	-1.348485000	1.071497000
1	-3.544672000	3.646463000	1.587308000
1	2.466168000	4.505867000	3.557962000
6	1.756291000	-2.739356000	0.675932000
6	-1.634208000	-1.576527000	0.382728000
6	3.026146000	-0.767750000	1.529960000
6	-3.718711000	-0.163971000	0.338491000
6	2.630053000	2.514946000	2.813642000
6	-2.581280000	-3.197669000	-1.347314000
6	-2.439493000	-2.781846000	-0.005794000
6	1.919845000	3.682929000	3.092929000
6	-3.998026000	1.136847000	0.666238000
6	4.360763000	1.088277000	2.577859000
6	4.045276000	2.339346000	3.041061000
6	-3.951041000	-5.079682000	-0.604231000
6	-0.448547000	-2.870035000	-2.636091000
6	-3.068482000	-3.519395000	1.010333000
6	-3.334642000	-4.349414000	-1.621819000
6	-3.819084000	-4.661295000	0.722468000
1	-2.014790000	-1.368480000	-2.379378000
1	0.009065000	-0.857029000	-2.686998000
1	0.300363000	5.985430000	3.589382000
1	-2.258079000	5.628030000	2.721913000
1	-0.009806000	-3.904581000	0.056525000
1	2.597034000	-3.429623000	0.633357000
1	-4.396412000	-0.925332000	-0.040241000
1	-4.952496000	1.658935000	0.610971000
1	5.331159000	0.592981000	2.569755000
1	4.702597000	3.083643000	3.488931000
1	-4.534605000	-5.971168000	-0.847083000
1	-2.958047000	-3.185243000	2.044819000
1	-3.438218000	-4.673509000	-2.660358000
1	-4.297557000	-5.220165000	1.530372000
6	1.003629000	2.233496000	-0.379071000
8	0.275468000	3.334168000	-0.920702000
8	2.120742000	2.122123000	-0.864631000
1	0.881399000	3.723414000	-1.586938000
8	-1.814941000	2.447615000	-2.430811000
8	-0.391220000	0.731936000	-2.637408000
6	-1.555090000	1.173472000	-2.874037000
8	-2.486173000	0.589687000	-3.450541000
6	1.816440000	-1.870255000	-2.740011000
6	2.491465000	-0.632196000	-2.694720000
6	2.570702000	-3.058949000	-2.775049000
6	3.883982000	-0.590373000	-2.675238000
6	3.966833000	-2.998016000	-2.754006000
6	4.635951000	-1.771429000	-2.703764000
1	1.908358000	0.287208000	-2.633378000
1	2.048451000	-4.010915000	-2.804035000
1	4.384304000	0.380049000	-2.620565000
1	4.538515000	-3.930094000	-2.774419000
1	5.727840000	-1.735581000	-2.683333000
1	-2.412255000	-2.727808000	-3.424093000
1	-1.044214000	2.743398000	-1.892468000

^{3}TS2$_{II}$:

26	-0.742956000	-1.221828000	19.310000000
8	-1.092924000	-6.428234000	15.149953000
7	-0.340849000	-3.152346000	18.868571000
7	1.138844000	-0.990856000	20.052286000
6	-2.872240000	-4.828116000	15.351744000
7	-0.543413000	-4.192025000	15.115546000
7	-1.219757000	0.590355000	20.063473000
7	-2.683040000	-1.586299000	18.900937000
6	-0.375846000	1.504286000	20.650523000
6	-1.213998000	-4.129008000	18.439340000
6	-3.702398000	-0.664489000	18.963654000
6	2.179385000	-1.871936000	19.897204000
6	-3.251922000	-2.751324000	18.432987000
6	-1.084816000	2.733088000	20.934301000
1	2.988092000	-3.746776000	19.300596000
6	-2.438807000	1.211907000	19.960069000
6	-2.367820000	2.551907000	20.500671000
6	-3.594053000	0.638817000	19.437670000
6	-0.503245000	-5.371683000	18.221462000
6	0.899877000	-3.746169000	18.913818000
1	-4.497136000	1.251680000	19.425027000
1	1.523798000	2.118337000	21.388723000
6	0.809882000	-5.126922000	18.495994000
6	-2.587843000	-3.966429000	18.228453000
6	2.077189000	-3.147612000	19.350244000
6	-4.664219000	-2.551070000	18.184338000
6	1.674490000	0.132822000	20.635941000
6	-3.535180000	-5.572826000	16.484490000
6	-3.394815000	-5.165492000	17.828749000

6	0.972669000	1.298550000	20.924661000
6	-4.940503000	-1.253451000	18.507315000
6	3.404125000	-1.297869000	20.410633000
6	3.089280000	-0.051862000	20.875978000
6	-4.900273000	-7.461621000	17.225333000
6	-1.403858000	-5.235455000	15.196247000
6	-4.019755000	-5.907006000	18.844236000
6	-4.286563000	-6.726399000	16.209682000
6	-4.769165000	-7.048466000	18.553678000
1	-2.980633000	-3.737135000	15.429095000
1	-0.946929000	-3.196908000	15.154132000
1	-0.642487000	3.610201000	21.404455000
1	-3.203098000	3.249858000	20.540573000
1	-0.955312000	-6.290848000	17.861859000
1	1.654584000	-5.809812000	18.427222000
1	-5.341788000	-3.314201000	17.809821000
1	-5.894057000	-0.729837000	18.455092000
1	4.371276000	-1.798960000	20.411944000
1	3.742426000	0.686990000	21.338094000
1	-5.481395000	-8.353852000	16.979362000
1	-3.907319000	-5.576378000	19.879631000
1	-4.390329000	-7.048434000	15.170545000
1	-5.246107000	-7.610914000	19.359909000
6	0.193678000	-0.406765000	17.447276000
8	-0.957266000	1.224458000	16.655371000
8	1.217146000	-0.300460000	16.928117000
1	-0.305928000	1.457081000	15.977966000
8	-2.679560000	0.082574000	15.392005000
8	-1.267220000	-1.650452000	15.245938000
6	-2.439593000	-1.159360000	15.009812000
8	-3.355456000	-1.812907000	14.441794000
6	0.855475000	-4.246278000	15.086987000
6	1.535717000	-3.009863000	15.105990000
6	1.610243000	-5.436344000	15.071712000
6	2.927647000	-2.967385000	15.137299000
6	3.006805000	-5.375955000	15.095907000
6	3.678201000	-4.150187000	15.134625000
1	0.939303000	-2.097537000	15.120364000
1	1.087785000	-6.388369000	15.054980000
1	3.430123000	-1.996948000	15.172033000
1	3.577434000	-6.308986000	15.089518000
1	4.770031000	-4.115867000	15.160945000
1	-3.365901000	-5.115543000	14.408752000
1	-1.787022000	0.610502000	16.023872000

^{3}IM2$_{II}$:

26	0.192971000	1.122224000	1.526823000
8	-0.136940000	-4.019209000	-2.671659000
7	0.599691000	-0.799778000	1.077657000
7	2.087705000	1.378202000	2.206952000
6	-1.926858000	-2.436949000	-2.464413000
7	0.401608000	-1.779439000	-2.693678000
7	-0.265354000	2.962569000	2.214043000
7	-1.739776000	0.775234000	1.098071000
6	0.582061000	3.881875000	2.789450000
6	-0.273814000	-1.772072000	0.639042000
6	-2.752680000	1.705460000	1.133873000
6	3.127420000	0.491748000	2.062719000
6	-2.309274000	-0.389855000	0.629873000
6	-0.122343000	5.115619000	3.058831000
1	3.933555000	-1.387786000	1.478473000
6	-1.482844000	3.588729000	2.103817000
6	-1.405837000	4.934506000	2.627073000
6	-2.639500000	3.015513000	1.587095000
6	0.437680000	-3.011055000	0.405635000
6	1.842148000	-1.390874000	1.107165000
1	-3.538021000	3.634244000	1.558229000
1	2.484452000	4.498096000	3.518474000
6	1.751642000	-2.767425000	0.677102000
6	-1.646197000	-1.605244000	0.424873000
6	3.022528000	-0.789244000	1.530387000
6	-3.716530000	-0.182831000	0.361268000
6	2.627553000	2.506738000	2.780899000
6	-2.589173000	-3.192103000	-1.338369000
6	-2.452160000	-2.799392000	0.010795000
6	1.930093000	3.675927000	3.062833000
6	-3.988211000	1.119944000	0.666822000
6	4.352847000	1.069086000	2.568352000
6	4.040992000	2.320322000	3.021344000
6	-3.960452000	-5.085347000	-0.615861000
6	-0.454866000	-2.827371000	-2.617221000
6	-3.079545000	-3.548645000	1.018725000
6	-3.343150000	-4.341959000	-1.623472000
6	-3.830827000	-4.685775000	0.716716000
1	-2.046092000	-1.344131000	-2.401515000
1	0.010882000	-0.768308000	-2.632958000
1	0.323864000	5.996784000	3.517433000
1	-2.238167000	5.636316000	2.657345000
1	-0.013834000	-3.925473000	0.033641000
1	2.597533000	-3.447130000	0.595035000
1	-4.393354000	-0.944699000	-0.016973000
1	-4.936729000	1.650156000	0.594673000
1	5.318649000	0.565616000	2.574005000
1	4.696022000	3.062494000	3.475209000
1	-4.543216000	-5.973808000	-0.871581000
1	-2.968016000	-3.227274000	2.057140000
1	-3.445966000	-4.654587000	-2.665616000
1	-4.310216000	-5.254847000	1.516810000
6	1.141503000	1.811458000	-0.462182000
8	-0.124462000	4.050918000	-1.298498000
8	2.121670000	2.056611000	-0.988756000
1	0.679840000	4.017511000	-1.832726000
8	-1.692999000	2.492546000	-2.562207000
8	-0.244103000	0.777657000	-2.502344000
6	-1.427602000	1.252908000	-2.817435000
8	-2.296691000	0.498904000	-3.358877000
6	1.800170000	-1.843752000	-2.739653000
6	2.487667000	-0.611597000	-2.786970000
6	2.552054000	-3.036160000	-2.708386000
6	3.879874000	-0.573418000	-2.765143000
6	3.948906000	-2.980936000	-2.697196000
6	4.626121000	-1.757975000	-2.716459000
1	1.890471000	0.299590000	-2.821535000
1	2.026330000	-3.985621000	-2.680561000
1	4.387398000	0.395019000	-2.782744000
1	4.515349000	-3.916095000	-2.665857000
1	5.718232000	-1.727151000	-2.697503000
1	-2.410244000	-2.733503000	-3.410251000
1	-0.730999000	3.376005000	-1.787744000

^{5}RC1$_{II}$:

26	-0.945586000	-0.686018000	19.887094000
8	-0.647481000	-6.536863000	15.749561000
7	-0.341630000	-2.619844000	19.390303000
7	0.907974000	-0.378016000	20.862295000
6	-2.031913000	-4.543899000	15.786846000
7	-0.167608000	-4.832805000	14.287318000
7	-1.650204000	1.033225000	20.825608000
7	-2.939409000	-1.336852000	19.611412000
6	-0.897081000	1.982490000	21.478529000
6	-1.110330000	-3.630715000	18.851800000
6	-4.030838000	-0.497307000	19.682769000
6	2.025757000	-1.164900000	20.701661000
6	-3.339956000	-2.476637000	18.955394000

6	-1.755038000	3.110034000	21.836575000
1	2.989013000	-2.958524000	20.040555000
6	-2.950070000	1.495767000	20.775703000
6	-3.013165000	2.811408000	21.403419000
6	-4.029305000	0.807437000	20.248544000
6	-0.253439000	-4.783997000	18.573443000
6	0.954770000	-3.093456000	19.489852000
1	-4.993769000	1.322288000	20.265515000
1	0.912997000	2.728082000	22.292780000
6	1.010685000	-4.446491000	18.953141000
6	-2.490034000	-3.591751000	18.632420000
6	2.029618000	-2.434652000	20.064542000
6	-4.728210000	-2.367598000	18.618085000
6	1.309712000	0.781282000	21.480134000
6	-2.971058000	-5.241733000	16.733653000
6	-3.169385000	-4.795776000	18.060218000
6	0.454797000	1.879902000	21.777717000
6	-5.159280000	-1.126635000	19.079703000
6	3.163135000	-0.503576000	21.257608000
6	2.713787000	0.720115000	21.748690000
6	-4.580939000	-7.046789000	17.094002000
6	-0.885641000	-5.424691000	15.279835000
6	-4.080740000	-5.498366000	18.875038000
6	-3.685775000	-6.361905000	16.274821000
6	-4.777871000	-6.611503000	18.410011000
1	-1.569851000	-3.667386000	16.261944000
1	-0.465808000	-3.862547000	14.001863000
1	-1.419715000	4.003943000	22.363137000
1	-3.918882000	3.409955000	21.504836000
1	-0.585706000	-5.717479000	18.126189000
1	1.911590000	-5.058431000	18.900721000
1	-5.313247000	-3.119526000	18.093361000
1	-6.156402000	-0.692687000	18.999310000
1	4.177978000	-0.902048000	21.276775000
1	3.296488000	1.500536000	22.238981000
1	-5.122570000	-7.915392000	16.710832000
1	-4.235704000	-5.149100000	19.898402000
1	-3.529064000	-6.699783000	15.247208000
1	-5.471728000	-7.138940000	19.069761000
6	-0.583735000	0.300532000	18.089719000
8	-1.605968000	0.305636000	17.383017000
8	0.550975000	0.746174000	17.968148000
1	-1.575450000	-0.310286000	15.784569000
8	-1.726001000	-0.694827000	14.884650000
8	-0.964212000	-2.336585000	13.606434000
6	-0.804389000	-1.687560000	14.684275000
8	0.067062000	-1.877371000	15.553311000
6	1.008409000	-5.283941000	13.681889000
6	1.630178000	-4.403538000	12.769425000
6	1.600448000	-6.539935000	13.923712000
6	2.806579000	-4.769428000	12.118831000
6	2.781837000	-6.890761000	13.263321000
6	3.394445000	-6.017628000	12.359898000
1	1.166846000	-3.430516000	12.593885000
1	1.126196000	-7.218714000	14.627120000
1	3.270645000	-4.071212000	11.417120000
1	3.228396000	-7.868882000	13.462510000
1	4.317114000	-6.303964000	11.849898000
1	-2.575365000	-4.155922000	14.909926000

^{5}IIM1$_{II}$:

26	-0.981049000	-0.738391000	19.964569000
8	-0.599014000	-6.472371000	15.748808000
7	-0.386497000	-2.685402000	19.577649000
7	0.857615000	-0.380858000	20.935664000
6	-1.974780000	-4.481849000	15.857888000
7	-0.187466000	-4.756951000	14.269241000
7	-1.672535000	1.052577000	20.737124000
7	-2.965455000	-1.364701000	19.646337000
6	-0.913897000	2.042891000	21.323182000
6	-1.138551000	-3.688858000	19.000024000
6	-4.052839000	-0.514638000	19.654235000
6	1.967026000	-1.193695000	20.859422000
6	-3.358687000	-2.523893000	19.016632000
6	-1.758737000	3.206231000	21.571682000
1	2.928319000	-3.022589000	20.302311000
6	-2.964095000	1.530496000	20.626345000
6	-3.014840000	2.891412000	21.144591000
6	-4.046705000	0.821006000	20.137083000
6	-0.276876000	-4.840170000	18.741632000
6	0.906234000	-3.160110000	19.708653000
1	-5.002878000	1.349163000	20.100477000
1	0.886078000	2.824984000	22.121625000
6	0.973253000	-4.511758000	19.172220000
6	-2.505875000	-3.641368000	18.727712000
6	1.972716000	-2.492342000	20.287465000
6	-4.738179000	-2.419243000	18.646597000
6	1.266030000	0.812979000	21.481825000
6	-2.928542000	-5.218999000	16.761143000
6	-3.161284000	-4.831049000	18.099902000
6	0.426721000	1.944843000	21.665291000
6	-5.171699000	-1.161126000	19.052601000
6	3.099301000	-0.514773000	21.403494000
6	2.659466000	0.746578000	21.795664000
6	-4.537276000	-7.045460000	17.019104000
6	-0.853928000	-5.355455000	15.287478000
6	-4.082006000	-5.569155000	18.870202000
6	-3.629576000	-6.324090000	16.246173000
6	-4.763358000	-6.666627000	18.347573000
1	-1.472762000	-3.646316000	16.367604000
1	-0.441024000	-3.711658000	14.051445000
1	-1.416673000	4.134337000	22.029768000
1	-3.912846000	3.508185000	21.182132000
1	-0.596765000	-5.762523000	18.262769000
1	1.874683000	-5.124117000	19.144622000
1	-5.314622000	-3.185904000	18.134404000
1	-6.162362000	-0.722831000	18.930769000
1	4.105784000	-0.927203000	21.476466000
1	3.242339000	1.550407000	22.245705000
1	-5.066198000	-7.900326000	16.589830000
1	-4.259026000	-5.260651000	19.903202000
1	-3.450359000	-6.621867000	15.209956000
1	-5.467198000	-7.223921000	18.971119000
6	-0.580120000	0.001240000	18.146166000
8	-1.549356000	-0.176994000	17.306071000
8	0.497285000	0.523681000	17.961367000
1	-1.272217000	-0.206946000	16.345709000
8	-1.076472000	-0.425924000	14.965010000
8	-0.756128000	-2.269973000	13.745787000
6	-0.521422000	-1.603992000	14.843505000
8	0.200119000	-2.087144000	15.765235000
6	0.948890000	-5.224141000	13.610592000
6	1.564277000	-4.332589000	12.700629000
6	1.513899000	-6.506611000	13.780752000
6	2.699634000	-4.711617000	11.988173000
6	2.654125000	-6.870593000	13.057805000
6	3.257249000	-5.985691000	12.159163000
1	1.120861000	-3.341059000	12.585430000
1	1.049722000	-7.194278000	14.482425000
1	3.157072000	-4.003391000	11.291562000
1	3.076601000	-7.868972000	13.203691000
1	4.147919000	-6.282158000	11.600062000
1	-2.513162000	-4.022584000	15.013034000

^{5}RC2$_{II}$:

26	0.355142000	1.264603000	1.128490000
8	-0.123898000	-4.094733000	-2.764530000
7	0.699169000	-0.814448000	0.895183000
7	2.194595000	1.343157000	2.203712000
6	-1.969696000	-2.563121000	-2.587206000
7	0.332209000	-1.839200000	-2.752756000
7	-0.209592000	3.013688000	2.219322000
7	-1.709079000	0.834757000	0.949743000
6	0.655207000	3.894168000	2.808201000
6	-0.207587000	-1.756238000	0.478683000
6	-2.724913000	1.741011000	1.107542000
6	3.204005000	0.429501000	2.093919000
6	-2.262366000	-0.325047000	0.470996000
6	-0.053962000	5.117102000	3.151667000
1	3.956865000	-1.499409000	1.597156000
6	-1.434653000	3.614243000	2.167656000
6	-1.351979000	4.942522000	2.754533000
6	-2.595656000	3.029122000	1.643763000
6	0.458931000	-3.045053000	0.360590000
6	1.900702000	-1.451234000	1.068489000
1	-3.507995000	3.628463000	1.689437000
1	2.579780000	4.459507000	3.516368000
6	1.764338000	-2.851271000	0.711325000
6	-1.582772000	-1.543253000	0.252585000
6	3.067052000	-0.866275000	1.572088000
6	-3.691735000	-0.135348000	0.294289000
6	2.721716000	2.472798000	2.769273000
6	-2.596830000	-3.238099000	-1.390338000
6	-2.416113000	-2.750880000	-0.077514000
6	2.013126000	3.650284000	3.049730000
6	-3.976535000	1.143674000	0.688074000
6	4.431154000	0.993845000	2.625408000
6	4.132053000	2.263760000	3.044284000
6	-3.951832000	-5.072890000	-0.507266000
6	-0.486416000	-2.919086000	-2.714819000
6	-3.021915000	-3.430632000	0.992588000
6	-3.360657000	-4.401280000	-1.578517000
6	-3.784768000	-4.581877000	0.790020000
1	-2.125983000	-1.476059000	-2.569032000
1	-0.131523000	-0.897640000	-2.710507000
1	0.389906000	5.983100000	3.641903000
1	-2.184312000	5.639456000	2.849501000
1	-0.012718000	-3.964399000	0.025574000
1	2.568629000	-3.585132000	0.738450000
1	-4.380104000	-0.890353000	-0.078001000
1	-4.944889000	1.642882000	0.702241000
1	5.390353000	0.478731000	2.670407000
1	4.795737000	2.994560000	3.505501000
1	-4.542848000	-5.974559000	-0.685955000
1	-2.880459000	-3.042072000	2.004025000
1	-3.493352000	-4.781764000	-2.594428000
1	-4.243158000	-5.092278000	1.640528000
6	1.122119000	2.368448000	-0.513804000
8	0.280389000	3.363504000	-1.060645000
8	2.254554000	2.368508000	-0.982175000
1	0.821144000	3.818445000	-1.740582000
8	-2.039491000	2.378872000	-2.214985000
8	-0.673976000	0.662877000	-2.664760000
6	-1.853699000	1.117357000	-2.729085000
8	-2.859836000	0.567082000	-3.206477000
6	1.731446000	-1.814557000	-2.690599000
6	2.330701000	-0.563646000	-2.444974000
6	2.550984000	-2.950621000	-2.830724000
6	3.713406000	-0.454207000	-2.323133000
6	3.938406000	-2.823272000	-2.713642000
6	4.531473000	-1.583049000	-2.456265000
1	1.697117000	0.313751000	-2.320119000
1	2.086867000	-3.916806000	-3.009540000
1	4.140410000	0.526166000	-2.099822000
1	4.563584000	-3.714259000	-2.820301000
1	5.616466000	-1.498178000	-2.357222000
1	-2.457598000	-2.943778000	-3.498840000
1	-1.193723000	2.669273000	-1.807618000

^{5}TS2$_{II}$:

26	-0.688638000	-1.199558000	19.015759000
8	-0.988180000	-6.401310000	15.178876000
7	-0.325272000	-3.257784000	18.780732000
7	1.219999000	-1.060485000	19.937851000
6	-2.825641000	-4.862315000	15.311891000
7	-0.509059000	-4.149127000	15.190408000
7	-1.185507000	0.607303000	20.000649000
7	-2.735258000	-1.609454000	18.879530000
6	-0.296015000	1.509446000	20.515883000
6	-1.242418000	-4.203172000	18.395559000
6	-3.743613000	-0.691987000	19.026786000
6	2.227295000	-1.978071000	19.815715000
6	-3.296992000	-2.772679000	18.414922000
6	-0.991301000	2.742619000	20.846628000
1	2.964692000	-3.917389000	19.340466000
6	-2.413169000	1.205627000	19.982419000
6	-2.305490000	2.553172000	20.516872000
6	-3.594616000	0.609075000	19.524463000
6	-0.578495000	-5.491598000	18.271372000
6	0.884690000	-3.888589000	18.909856000
1	-4.501541000	1.215452000	19.577403000
1	1.656494000	2.103672000	21.114177000
6	0.737137000	-5.292923000	18.576588000
6	-2.619338000	-3.989352000	18.187055000
6	2.071909000	-3.288446000	19.341687000
6	-4.724362000	-2.574201000	18.240002000
6	1.770142000	0.091264000	20.438412000
6	-3.525981000	-5.598879000	16.428550000
6	-3.439237000	-5.176397000	17.773688000
6	1.071933000	1.276653000	20.705007000
6	-4.999099000	-1.287378000	18.619545000
6	3.473399000	-1.392342000	20.271049000
6	3.189977000	-0.108521000	20.656906000
6	-4.946312000	-7.461927000	17.133354000
6	-1.340273000	-5.220168000	15.225763000
6	-4.119569000	-5.895724000	18.769081000
6	-4.279228000	-6.747303000	16.136505000
6	-4.870667000	-7.031524000	18.460399000
1	-2.977281000	-3.772281000	15.353663000
1	-0.937378000	-3.165465000	15.226586000
1	-0.526261000	3.626083000	21.282626000
1	-3.134262000	3.252163000	20.624475000
1	-1.060270000	-6.414234000	17.962081000
1	1.543304000	-6.024442000	18.583995000
1	-5.420204000	-3.328799000	17.881171000
1	-5.964468000	-0.782799000	18.632272000
1	4.434747000	-1.904497000	20.291596000
1	3.871958000	0.639193000	21.060201000
1	-5.526574000	-8.350702000	16.873110000
1	-4.047781000	-5.553238000	19.804247000
1	-4.343164000	-7.081156000	15.097758000
1	-5.390491000	-7.576868000	19.251774000
6	0.214042000	-0.299280000	17.312277000
8	-1.121989000	1.271346000	16.730978000
8	1.184224000	-0.070666000	16.736203000
1	-0.538599000	1.648609000	16.056557000
8	-2.769973000	0.069737000	15.408429000
8	-1.280123000	-1.602515000	15.310468000

6	-2.477334000	-1.164416000	15.067754000
8	-3.361186000	-1.884546000	14.521818000
6	0.891083000	-4.163395000	15.209125000
6	1.535274000	-2.908235000	15.186454000
6	1.679663000	-5.329434000	15.279395000
6	2.923050000	-2.821220000	15.267431000
6	3.071826000	-5.225358000	15.351278000
6	3.706337000	-3.979325000	15.353798000
1	0.911636000	-2.016422000	15.129887000
1	1.185284000	-6.296138000	15.289702000
1	3.395272000	-1.835136000	15.270749000
1	3.668646000	-6.139818000	15.413324000
1	4.794927000	-3.910842000	15.419532000
1	-3.263876000	-5.192087000	14.355183000
1	-1.871739000	0.673858000	16.109825000

^{5}IM2$_{II}$:

26	0.142634000	1.205646000	1.141544000
8	-0.083829000	-3.994283000	-2.690314000
7	0.537524000	-0.883805000	1.024871000
7	2.064003000	1.422228000	2.027781000
6	-1.827984000	-2.375073000	-2.387416000
7	0.538078000	-1.792342000	-2.512356000
7	-0.351447000	3.041350000	2.079019000
7	-1.895422000	0.694176000	1.165100000
6	0.521465000	3.984098000	2.546431000
6	-0.352758000	-1.858770000	0.664059000
6	-2.915495000	1.595377000	1.322550000
6	3.086246000	0.521493000	1.898158000
6	-2.446352000	-0.486526000	0.739923000
6	-0.208113000	5.195127000	2.889891000
1	3.866317000	-1.409340000	1.464348000
6	-1.606008000	3.585077000	2.121908000
6	-1.527403000	4.947691000	2.627248000
6	-2.783515000	2.922557000	1.759340000
6	0.353281000	-3.125968000	0.515977000
6	1.772344000	-1.467338000	1.114544000
1	-3.709630000	3.496299000	1.840969000
1	2.481494000	4.643656000	3.051284000
6	1.668198000	-2.880665000	0.787853000
6	-1.738545000	-1.681782000	0.486503000
6	2.951600000	-0.812500000	1.484128000
6	-3.883035000	-0.326797000	0.618846000
6	2.610090000	2.605514000	2.447302000
6	-2.561501000	-3.203390000	-1.359466000
6	-2.522835000	-2.866498000	0.011022000
6	1.899734000	3.791495000	2.692503000
6	-4.172776000	0.962044000	0.984399000
6	4.338564000	1.152803000	2.267336000
6	4.042654000	2.446925000	2.608505000
6	-3.960379000	-5.131628000	-0.825550000
6	-0.371195000	-2.810412000	-2.536099000
6	-3.216073000	-3.660369000	0.937099000
6	-3.279410000	-4.341867000	-1.754558000
6	-3.932514000	-4.787703000	0.528741000
1	-1.908850000	-1.296799000	-2.189730000
1	0.179334000	-0.843441000	-2.354514000
1	0.239339000	6.104340000	3.290427000
1	-2.378347000	5.613375000	2.768508000
1	-0.103969000	-4.067147000	0.222876000
1	2.499755000	-3.583478000	0.774814000
1	-4.575231000	-1.102716000	0.300056000
1	-5.148631000	1.444767000	1.025576000
1	5.311325000	0.662004000	2.267011000
1	4.725578000	3.227912000	2.941626000
1	-4.514998000	-6.012047000	-1.159465000
1	-3.183441000	-3.385116000	1.993977000
1	-3.305137000	-4.607206000	-2.814344000
1	-4.464478000	-5.394865000	1.265165000
6	0.727674000	1.642198000	-0.822751000
8	0.261280000	4.616600000	-1.359721000
8	1.828032000	2.032307000	-1.183359000
1	1.037420000	4.039092000	-1.321016000
8	-1.654555000	2.722304000	-1.683638000
8	-0.061592000	1.131681000	-1.933862000
6	-1.402220000	1.556516000	-2.062088000
8	-2.159629000	0.728696000	-2.589478000
6	1.939323000	-1.891201000	-2.583513000
6	2.674142000	-0.690552000	-2.613482000
6	2.633370000	-3.116200000	-2.602715000
6	4.067805000	-0.716589000	-2.636194000
6	4.030511000	-3.124199000	-2.633395000
6	4.760610000	-1.932449000	-2.645726000
1	2.152859000	0.266078000	-2.581566000
1	2.068132000	-4.042576000	-2.585683000
1	4.615974000	0.229321000	-2.641800000
1	4.553369000	-4.084429000	-2.642404000
1	5.852840000	-1.950123000	-2.663360000
1	-2.286831000	-2.549910000	-3.374989000
1	-0.459690000	3.947401000	-1.471268000

[Fe^{II}(*ortho*-amide-TPP)(phenol)]$^{2-}$

Solvent = water:

^{5}RC1$_{II,phenol}$:

26	-0.403372000	-0.948092000	19.755041000
8	-1.222446000	-5.075044000	14.970489000
7	-0.290306000	-2.975784000	19.298083000
7	1.616114000	-0.989491000	20.343135000
6	-2.712309000	-3.363302000	15.770002000
7	-0.293399000	-3.145206000	15.803900000
7	-0.598230000	0.863813000	20.773936000
7	-2.498064000	-1.132660000	19.738010000
6	0.435141000	1.672450000	21.178072000
6	-1.308752000	-3.754474000	18.806137000
6	-3.391758000	-0.118421000	20.031451000
6	2.508434000	-2.010892000	20.079092000
6	-3.209519000	-2.146795000	19.149760000
6	-0.105083000	2.956023000	21.612761000
1	2.961496000	-3.993133000	19.423947000
6	-1.773490000	1.579067000	20.927702000
6	-1.462061000	2.894732000	21.460469000
6	-3.042228000	1.128299000	20.603064000
6	-0.768550000	-5.055743000	18.421571000
6	0.878027000	-3.718296000	19.222847000
1	-3.864645000	1.829157000	20.765713000
1	2.486200000	2.089544000	21.538187000
6	0.571045000	-5.029057000	18.680126000
6	-2.652801000	-3.381736000	18.691355000
6	2.144583000	-3.283345000	19.576854000
6	-4.595085000	-1.776612000	19.070929000
6	2.341914000	0.079379000	20.801866000
6	-3.593944000	-4.311886000	16.548548000
6	-3.566753000	-4.313901000	17.961795000
6	1.784875000	1.328777000	21.186530000
6	-4.705647000	-0.509911000	19.633377000
6	3.835420000	-1.577607000	20.386376000
6	3.734012000	-0.266616000	20.839440000
6	-5.243665000	-6.106196000	16.579158000
6	-1.332103000	-3.962891000	15.479450000
6	-4.402455000	-5.202026000	18.655782000
6	-4.426713000	-5.217230000	15.875951000
6	-5.235927000	-6.095492000	17.977076000
1	-2.615020000	-2.408251000	16.298755000

1	-0.540988000	-2.265495000	16.278198000
1	0.491677000	3.788078000	21.987292000
1	-2.199566000	3.665924000	21.683438000
1	-1.349189000	-5.868799000	17.991988000
1	1.302788000	-5.817924000	18.509054000
1	-5.388796000	-2.391710000	18.651574000
1	-5.603947000	0.097334000	19.745323000
1	4.735766000	-2.183145000	20.279514000
1	4.537796000	0.392581000	21.168450000
1	-5.888131000	-6.802442000	16.036502000
1	-4.385345000	-5.191358000	19.748469000
1	-4.435567000	-5.219457000	14.783059000
1	-5.874302000	-6.783198000	18.537601000
6	-0.163343000	-0.186414000	17.910969000
8	-0.920736000	-0.717078000	17.055078000
8	0.668799000	0.706636000	17.815371000
6	1.082059000	-3.401532000	15.722464000
6	1.957631000	-2.365423000	16.104249000
6	1.623357000	-4.631095000	15.303608000
6	3.336530000	-2.562157000	16.086946000
6	3.008940000	-4.812555000	15.289922000
6	3.876009000	-3.788736000	15.682745000
1	1.550338000	-1.408848000	16.432685000
1	0.947637000	-5.427943000	15.006601000
1	3.995206000	-1.749384000	16.403259000
1	3.413703000	-5.776156000	14.968717000
1	4.957470000	-3.942934000	15.672996000
1	-3.169504000	-3.164957000	14.786736000
6	-3.401735000	1.532298000	16.590569000
6	-4.719606000	2.001275000	16.436028000
6	-5.110139000	3.211875000	17.009889000
6	-4.198724000	3.978911000	17.746802000
6	-2.886827000	3.516124000	17.892542000
6	-2.481378000	2.310042000	17.317660000
1	-5.426524000	1.389820000	15.870342000
1	-6.139888000	3.557422000	16.883678000
1	-4.508473000	4.922768000	18.201144000
1	-2.163241000	4.094329000	18.472858000
1	-1.453875000	1.965413000	17.441218000
8	-3.073959000	0.356671000	16.019831000
1	-2.199482000	0.028013000	16.362044000

^{5}IM1$_{\mathrm{II,phenol}}$:

26	-0.072795000	-0.654611000	19.993024000
8	-1.732370000	-4.191133000	15.072712000
7	-0.241178000	-2.714639000	19.402696000
7	1.972724000	-1.115025000	20.432492000
6	-2.861658000	-2.370552000	16.170418000
7	-0.452103000	-2.485500000	15.929410000
7	0.050500000	0.766218000	21.574239000
7	-2.159292000	-0.828186000	20.456156000
6	1.200562000	1.373379000	21.992538000
6	-1.388545000	-3.311679000	18.955603000
6	-2.880743000	0.167685000	21.055314000
6	2.700364000	-2.166251000	19.950073000
6	-3.043742000	-1.633696000	19.793773000
6	0.872324000	2.549691000	22.781629000
1	2.858934000	-4.059355000	18.991987000
6	-0.997468000	1.501944000	22.048711000
6	-0.494691000	2.627707000	22.819063000
6	-2.350991000	1.227389000	21.807052000
6	-1.066516000	-4.597585000	18.356302000
6	0.798059000	-3.552515000	19.095161000
1	-3.073759000	1.926565000	22.234464000
1	3.321426000	1.512181000	22.123240000
6	0.289121000	-4.747899000	18.450022000
6	-2.695014000	-2.786043000	19.060681000
6	2.154831000	-3.297235000	19.332119000
6	-4.396130000	-1.125496000	19.976174000
6	2.851343000	-0.245053000	21.017059000
6	-3.844966000	-3.268713000	16.880231000
6	-3.770009000	-3.470261000	18.277044000
6	2.498860000	0.919945000	21.715260000
6	-4.294411000	-0.015945000	20.771183000
6	4.113050000	-1.946026000	20.210737000
6	4.207785000	-0.748373000	20.868135000
6	-5.773714000	-4.758672000	16.787515000
6	-1.627420000	-3.122734000	15.670827000
6	-4.707680000	-4.302161000	18.906299000
6	-4.850022000	-3.923412000	16.154763000
6	-5.705126000	-4.947388000	18.170941000
1	-2.565824000	-1.529532000	16.805803000
1	-0.505603000	-1.587424000	16.438956000
1	1.597205000	3.220087000	23.242822000
1	-1.112055000	3.374621000	23.317675000
1	-1.786420000	-5.282179000	17.915251000
1	0.899018000	-5.580243000	18.102337000
1	-5.296916000	-1.565346000	19.554781000
1	-5.096912000	0.628972000	21.128208000
1	4.916785000	-2.628047000	19.934532000
1	5.105203000	-0.253671000	21.238894000
1	-6.550151000	-5.257111000	16.201858000
1	-4.644450000	-4.444692000	19.987683000
1	-4.909319000	-3.764739000	15.075420000
1	-6.425529000	-5.594819000	18.676769000
6	-0.292064000	0.645846000	18.288096000
8	-0.389944000	0.075085000	17.046432000
8	-0.528062000	1.854350000	18.363219000
6	0.853612000	-2.931751000	15.685390000
6	1.905190000	-2.106916000	16.133309000
6	1.163290000	-4.150644000	15.053715000
6	3.230975000	-2.497257000	15.964629000
6	2.499586000	-4.528640000	14.892272000
6	3.541522000	-3.713644000	15.344254000
1	1.655064000	-1.171544000	16.635540000
1	0.352043000	-4.787048000	14.712030000
1	4.030078000	-1.849646000	16.334425000
1	2.725228000	-5.481314000	14.405328000
1	4.581928000	-4.021269000	15.216012000
1	-3.344363000	-1.931470000	15.283368000
6	-2.818395000	0.980212000	15.283267000
6	-3.630157000	0.574212000	14.176109000
6	-4.972108000	0.227616000	14.331891000
6	-5.590448000	0.272191000	15.590267000
6	-4.828256000	0.696578000	16.689673000
6	-3.488933000	1.053063000	16.548416000
1	-3.148853000	0.516261000	13.194549000
1	-5.546993000	-0.093917000	13.456586000
1	-6.639056000	-0.011315000	15.709848000
1	-5.286079000	0.741111000	17.683105000
1	-2.906520000	1.381002000	17.412884000
8	-1.556998000	1.241646000	15.145743000
1	-0.820477000	0.702682000	16.317756000

^{5}RC2$_{\mathrm{II,phenol}}$:

26	0.566365000	0.534302000	1.295776000
8	-0.107677000	-3.508469000	-3.550635000
7	0.598108000	-1.523211000	0.808807000
7	2.475163000	0.288457000	2.179983000
6	-1.523038000	-1.683547000	-2.888233000
7	0.902441000	-1.650953000	-2.654935000
7	0.340894000	2.232692000	2.541344000
7	-1.535481000	0.399546000	1.191842000
6	1.350131000	2.929005000	3.149244000

6	-0.416711000	-2.234709000	0.229736000
6	-2.405183000	1.400867000	1.540243000
6	3.321701000	-0.761377000	1.944296000
6	-2.252681000	-0.557245000	0.524487000
6	0.821729000	4.169025000	3.693815000
1	3.755616000	-2.715586000	1.228654000
6	-0.808903000	2.959938000	2.687831000
6	-0.517141000	4.187136000	3.409109000
6	-2.072278000	2.575894000	2.224721000
6	0.068576000	-3.558381000	-0.131260000
6	1.707884000	-2.327750000	0.829764000
1	-2.888783000	3.278598000	2.400399000
1	3.375819000	3.164887000	3.759083000
6	1.382250000	-3.614538000	0.241994000
6	-1.733217000	-1.771312000	0.029245000
6	2.964664000	-1.969625000	1.329472000
6	-3.642811000	-0.142018000	0.439704000
6	3.193404000	1.264141000	2.816310000
6	-2.535954000	-2.579931000	-2.211959000
6	-2.635817000	-2.626372000	-0.803905000
6	2.677404000	2.493164000	3.254836000
6	-3.737315000	1.062701000	1.080741000
6	4.642909000	-0.439199000	2.451162000
6	4.563158000	0.817903000	2.991015000
6	-4.299120000	-4.255196000	-2.376182000
6	-0.171090000	-2.385279000	-3.062915000
6	-3.576470000	-3.477472000	-0.206658000
6	-3.370265000	-3.404047000	-2.979564000
6	-4.405818000	-4.290555000	-0.983118000
1	-1.409799000	-0.738891000	-2.343293000
1	0.696923000	-0.731772000	-2.252623000
1	1.405425000	4.915771000	4.231325000
1	-1.250145000	4.951568000	3.663945000
1	-0.522068000	-4.331356000	-0.616403000
1	2.078038000	-4.444194000	0.128965000
1	-4.437648000	-0.707342000	-0.041254000
1	-4.621518000	1.681247000	1.223307000
1	5.509420000	-1.097861000	2.403745000
1	5.353175000	1.394216000	3.471634000
1	-4.941453000	-4.887497000	-2.993890000
1	-3.649037000	-3.500755000	0.883141000
1	-3.289118000	-3.372547000	-4.068723000
1	-5.131480000	-4.950114000	-0.501247000
6	1.194638000	1.439334000	-0.497643000
8	0.473335000	1.100072000	-1.725814000
8	2.121054000	2.200300000	-0.701224000
6	2.252628000	-2.034361000	-2.599680000
6	3.173532000	-1.099878000	-2.088666000
6	2.717220000	-3.300715000	-2.999619000
6	4.520712000	-1.430424000	-1.961448000
6	4.072095000	-3.616861000	-2.868177000
6	4.982944000	-2.693446000	-2.347341000
1	2.828031000	-0.118195000	-1.767419000
1	2.007867000	-4.021973000	-3.394302000
1	5.212925000	-0.693671000	-1.546559000
1	4.416277000	-4.607451000	-3.177053000
1	6.038951000	-2.953002000	-2.244196000
1	-1.874245000	-1.441019000	-3.904005000
6	-2.330623000	3.110131000	-1.290974000
6	-1.301615000	3.916082000	-0.772898000
6	-1.607852000	5.133750000	-0.162218000
6	-2.930239000	5.577633000	-0.066993000
6	-3.952168000	4.782607000	-0.601280000
6	-3.659101000	3.562258000	-1.209903000
1	-0.266968000	3.576439000	-0.821431000
1	-0.796953000	5.736245000	0.255100000
1	-3.163030000	6.529248000	0.415962000
1	-4.992278000	5.113893000	-0.538654000
1	-4.450246000	2.927022000	-1.613096000
8	-2.103642000	1.912879000	-1.867816000
1	0.924210000	1.591299000	-2.444935000
1	-1.163381000	1.635886000	-1.757821000

^{5}TS2$_{II,phenol}$:

26	-0.465348000	-0.909453000	19.723445000
8	-1.255788000	-4.954035000	14.744096000
7	-0.390449000	-2.902842000	19.065002000
7	1.441733000	-1.162397000	20.582816000
6	-2.693891000	-3.118861000	15.326798000
7	-0.268905000	-3.004502000	15.461162000
7	-0.690603000	0.775597000	20.968666000
7	-2.528589000	-0.996016000	19.488752000
6	0.304684000	1.446030000	21.628299000
6	-1.401233000	-3.603671000	18.463984000
6	-3.402390000	0.004336000	19.841048000
6	2.305378000	-2.187650000	20.289188000
6	-3.239375000	-1.939869000	18.792554000
6	-0.231987000	2.672741000	22.190775000
1	2.771569000	-4.089372000	19.463681000
6	-1.838367000	1.511582000	21.093335000
6	-1.560116000	2.711943000	21.860459000
6	-3.086017000	1.155069000	20.570064000
6	-0.904730000	-4.910683000	18.061554000
6	0.725782000	-3.699198000	19.059024000
1	-3.903979000	1.856487000	20.739519000
1	2.315491000	1.648380000	22.295256000
6	0.410698000	-4.965746000	18.424836000
6	-2.722653000	-3.148931000	18.284055000
6	1.971549000	-3.357995000	19.593915000
6	-4.624876000	-1.518687000	18.697372000
6	2.148141000	-0.211241000	21.274503000
6	-3.647023000	-4.007473000	16.092438000
6	-3.653297000	-4.027010000	17.504338000
6	1.625386000	0.998608000	21.752892000
6	-4.724003000	-0.320599000	19.349416000
6	3.614228000	-1.880156000	20.826917000
6	3.517092000	-0.655503000	21.436104000
6	-5.392839000	-5.712540000	16.080759000
6	-1.327839000	-3.794207000	15.145831000
6	-4.538353000	-4.880759000	18.179361000
6	-4.519230000	-4.860693000	15.401386000
6	-5.405306000	-5.721837000	17.478157000
1	-2.593936000	-2.137710000	15.806031000
1	-0.478166000	-2.019450000	15.759243000
1	0.339311000	3.397499000	22.769682000
1	-2.294277000	3.476610000	22.110814000
1	-1.488722000	-5.669608000	17.548334000
1	1.114766000	-5.782784000	18.279225000
1	-5.413324000	-2.073755000	18.195086000
1	-5.605834000	0.302076000	19.483107000
1	4.488753000	-2.524866000	20.748631000
1	4.297419000	-0.098949000	21.953896000
1	-6.064898000	-6.366046000	15.519220000
1	-4.537872000	-4.881888000	19.271903000
1	-4.511066000	-4.851045000	14.308733000
1	-6.086298000	-6.381437000	18.021267000
6	0.502886000	0.183862000	18.189460000
8	-0.700914000	-0.398160000	16.177276000
8	1.322278000	0.861548000	17.777176000
6	1.081732000	-3.368002000	15.537924000
6	1.981074000	-2.388490000	16.004966000
6	1.578119000	-4.645181000	15.214045000
6	3.332482000	-2.682111000	16.167306000
6	2.937311000	-4.925270000	15.379660000

6	3.823690000	-3.956449000	15.859580000
1	1.592931000	-1.403064000	16.257226000
1	0.887366000	-5.401830000	14.853747000
1	4.005869000	-1.909275000	16.546656000
1	3.305699000	-5.924330000	15.131156000
1	4.882974000	-4.189342000	15.990198000
1	-3.094089000	-2.952167000	14.313615000
6	-3.246190000	1.461970000	16.784321000
6	-2.224045000	2.280148000	17.336292000
6	-2.532618000	3.428369000	18.063850000
6	-3.860653000	3.826864000	18.259594000
6	-4.880961000	3.047861000	17.696645000
6	-4.585197000	1.894287000	16.972778000
1	-1.185915000	1.981298000	17.194376000
1	-1.718429000	4.019606000	18.493959000
1	-4.096031000	4.725107000	18.835331000
1	-5.926818000	3.341519000	17.830971000
1	-5.383060000	1.278449000	16.550239000
8	-2.994338000	0.357619000	16.115879000
1	-0.229322000	0.129341000	15.515614000
1	-1.852814000	0.014682000	16.153084000

^{5}IM2$_{II,phenol}$:

26	0.568730000	0.558833000	1.442140000
8	-0.225256000	-3.603478000	-3.518646000
7	0.666824000	-1.424076000	0.752912000
7	2.474264000	0.312484000	2.291961000
6	-1.673574000	-1.756824000	-2.989655000
7	0.748950000	-1.625582000	-2.868866000
7	0.328452000	2.240996000	2.694626000
7	-1.482789000	0.480135000	1.180281000
6	1.319016000	2.909328000	3.363190000
6	-0.345771000	-2.131739000	0.161884000
6	-2.365883000	1.471761000	1.538331000
6	3.347204000	-0.705117000	1.994496000
6	-2.186741000	-0.467408000	0.478825000
6	0.774220000	4.125574000	3.941872000
1	3.826193000	-2.606323000	1.173369000
6	-0.822669000	2.968085000	2.826591000
6	-0.553481000	4.160124000	3.610977000
6	-2.064211000	2.613137000	2.287871000
6	0.148710000	-3.446062000	-0.221827000
6	1.780307000	-2.223437000	0.760110000
1	-2.888489000	3.305537000	2.461573000
1	3.328846000	3.116128000	4.030218000
6	1.463580000	-3.498053000	0.141639000
6	-1.667365000	-1.680032000	-0.021107000
6	3.022783000	-1.877753000	1.297623000
6	-3.572897000	-0.053684000	0.381409000
6	3.173259000	1.267007000	2.990923000
6	-2.615119000	-2.616042000	-2.177702000
6	-2.605830000	-2.580863000	-0.766320000
6	2.641909000	2.468252000	3.481642000
6	-3.681649000	1.141383000	1.038611000
6	4.651010000	-0.389123000	2.534860000
6	4.543719000	0.832683000	3.150069000
6	-4.360808000	-4.319810000	-2.106812000
6	-0.305919000	-2.432672000	-3.155980000
6	-3.484965000	-3.408038000	-0.050855000
6	-3.494252000	-3.494915000	-2.826819000
6	-4.359075000	-4.274906000	-0.710057000
1	-1.575338000	-0.755808000	-2.551844000
1	0.526773000	-0.642851000	-2.610968000
1	1.340451000	4.845755000	4.531308000
1	-1.292737000	4.917298000	3.869006000
1	-0.436589000	-4.211339000	-0.723790000
1	2.166162000	-4.318391000	0.007976000
1	-4.356470000	-0.610895000	-0.125854000
1	-4.567814000	1.757235000	1.172913000
1	5.531041000	-1.026024000	2.454621000
1	5.319160000	1.391954000	3.672133000
1	-5.038463000	-4.994071000	-2.636093000
1	-3.473444000	-3.367534000	1.040840000
1	-3.497456000	-3.526568000	-3.919062000
1	-5.034495000	-4.912619000	-0.134841000
6	1.564994000	1.676350000	-0.056176000
8	0.291573000	1.038744000	-2.282555000
8	2.363564000	2.338311000	-0.519346000
6	2.104010000	-1.967135000	-2.771360000
6	2.981899000	-0.967716000	-2.307026000
6	2.620240000	-3.240878000	-3.073235000
6	4.335724000	-1.239173000	-2.127134000
6	3.981544000	-3.498594000	-2.889925000
6	4.848024000	-2.510080000	-2.413786000
1	2.577817000	0.015679000	-2.069931000
1	1.943519000	-4.012109000	-3.429753000
1	4.993685000	-0.452184000	-1.750182000
1	4.367458000	-4.495044000	-3.121275000
1	5.909107000	-2.725587000	-2.269149000
1	-2.084637000	-1.633782000	-4.004533000
6	-2.259910000	2.926050000	-1.566353000
6	-1.201341000	3.716984000	-1.018465000
6	-1.454868000	4.830183000	-0.221153000
6	-2.765529000	5.238917000	0.061761000
6	-3.823509000	4.500852000	-0.488720000
6	-3.584201000	3.380762000	-1.281283000
1	-0.174398000	3.414768000	-1.228524000
1	-0.610736000	5.388850000	0.196273000
1	-2.957656000	6.109936000	0.692970000
1	-4.857134000	4.802035000	-0.287484000
1	-4.415123000	2.800344000	-1.692029000
8	-2.048292000	1.868920000	-2.290053000
1	0.741111000	1.547528000	-2.972420000
1	-0.704950000	1.411032000	-2.285084000

[Fe^{II}(*ortho*-urea-TPP)(HCO_3)]$^{3-}$

Solvent = water:

^{3}RC1$_{III}$:

26	-0.896164000	-0.859808000	19.791686000
8	-0.787363000	-6.652324000	15.577379000
7	-0.403353000	-2.771189000	19.387656000
7	0.946400000	-0.522304000	20.610938000
7	-2.089616000	-4.794285000	15.938939000
7	-0.264023000	-4.643957000	14.561107000
7	-1.476017000	0.935212000	20.529885000
7	-2.826976000	-1.346905000	19.399656000
6	-0.690460000	1.907032000	21.127209000
6	-1.198510000	-3.781737000	18.864228000
6	-3.893072000	-0.469512000	19.428187000
6	2.031285000	-1.365512000	20.515567000
6	-3.324377000	-2.529488000	18.874010000
6	-1.480226000	3.100288000	21.384603000
1	2.932042000	-3.225278000	19.954108000
6	-2.734508000	1.488751000	20.408284000
6	-2.740645000	2.842711000	20.938974000
6	-3.847083000	0.860537000	19.893282000
6	-0.388135000	-4.965063000	18.610109000
6	0.875786000	-3.291614000	19.462237000
1	-4.779820000	1.428538000	19.862668000
1	1.138986000	2.638936000	21.911141000
6	0.887490000	-4.657237000	18.966733000
6	-2.561310000	-3.703848000	18.622029000
6	1.995328000	-2.663055000	19.964384000

6	-4.712992000	-2.391038000	18.576846000
6	1.423710000	0.636261000	21.195527000
6	-2.998593000	-5.434967000	16.799731000
6	-3.270979000	-4.902730000	18.084267000
6	0.644696000	1.784784000	21.442924000
6	-5.069958000	-1.096191000	18.928633000
6	3.198360000	-0.747503000	21.057154000
6	2.813422000	0.515352000	21.489573000
6	-4.582781000	-7.245189000	17.200326000
6	-1.029187000	-5.459683000	15.371040000
6	-4.227635000	-5.555028000	18.883685000
6	-3.656500000	-6.605646000	16.379576000
6	-4.879743000	-6.713022000	18.460516000
1	-2.071328000	-3.744613000	15.928710000
1	-0.638032000	-3.699772000	14.312726000
1	-1.095782000	4.005396000	21.854825000
1	-3.616347000	3.491447000	20.965704000
1	-0.756428000	-5.897697000	18.191205000
1	1.774290000	-5.289285000	18.917015000
1	-5.343905000	-3.166354000	18.149588000
1	-6.047596000	-0.620066000	18.851810000
1	4.184270000	-1.210044000	21.107399000
1	3.422978000	1.287966000	21.958295000
1	-5.081591000	-8.153274000	16.851927000
1	-4.441652000	-5.140726000	19.871492000
1	-3.429238000	-7.004715000	15.390643000
1	-5.607362000	-7.202888000	19.112227000
6	-0.617807000	-0.043877000	18.008053000
8	-1.501987000	0.628405000	17.437083000
8	0.516683000	-0.368971000	17.646059000
1	-1.412064000	0.132858000	15.850281000
8	-1.214015000	-0.172111000	14.916635000
8	-0.968700000	-2.081050000	13.808038000
6	-1.413602000	-1.512368000	14.841554000
8	-2.016052000	-2.075384000	15.789581000
6	0.994976000	-4.920114000	14.028003000
6	1.620795000	-3.885704000	13.293058000
6	1.683283000	-6.141667000	14.187279000
6	2.885361000	-4.071488000	12.738446000
6	2.953410000	-6.307380000	13.627225000
6	3.567568000	-5.284107000	12.899567000
1	1.087769000	-2.939228000	13.180941000
1	1.207356000	-6.942692000	14.745770000
1	3.345929000	-3.254731000	12.175582000
1	3.469110000	-7.262160000	13.764421000
1	4.559691000	-5.427006000	12.465082000

^{3}IM1$_{III}$:

26	-0.878590000	-0.878872000	19.751818000
8	-0.748652000	-6.560304000	15.667507000
7	-0.405158000	-2.754692000	19.360059000
7	1.006762000	-0.575116000	20.612894000
7	-2.121364000	-4.732254000	15.907193000
7	-0.253110000	-4.566311000	14.600658000
7	-1.411507000	0.902408000	20.405157000
7	-2.883776000	-1.411794000	19.514998000
6	-0.591569000	1.899616000	20.918247000
6	-1.215369000	-3.754821000	18.826814000
6	-3.942287000	-0.540393000	19.594444000
6	2.061449000	-1.451595000	20.583126000
6	-3.376927000	-2.571422000	18.968381000
6	-1.363151000	3.108107000	21.148406000
1	2.893484000	-3.350662000	20.059588000
6	-2.686961000	1.451302000	20.360002000
6	-2.650977000	2.830713000	20.808513000
6	-3.848351000	0.800232000	20.000689000
6	-0.419649000	-4.945851000	18.573937000
6	0.861238000	-3.316578000	19.480148000
1	-4.773246000	1.380573000	20.023014000
1	1.246612000	2.662558000	21.645986000
6	0.850175000	-4.674206000	18.972833000
6	-2.590698000	-3.699025000	18.621329000
6	1.985453000	-2.744230000	20.042018000
6	-4.787490000	-2.440444000	18.727732000
6	1.501172000	0.602934000	21.113739000
6	-3.022308000	-5.391261000	16.759013000
6	-3.289458000	-4.894494000	18.059753000
6	0.745853000	1.780593000	21.241676000
6	-5.140991000	-1.167888000	19.129253000
6	3.240721000	-0.833844000	21.112764000
6	2.889158000	0.461294000	21.444443000
6	-4.592657000	-7.223810000	17.138436000
6	-1.023060000	-5.380749000	15.403366000
6	-4.228307000	-5.573762000	18.858185000
6	-3.679998000	-6.557469000	16.323754000
6	-4.877160000	-6.726016000	18.415158000
1	-2.209412000	-3.657327000	15.772054000
1	-0.655076000	-3.614061000	14.301212000
1	-0.948540000	4.034348000	21.544925000
1	-3.523653000	3.480705000	20.864667000
1	-0.797149000	-5.864877000	18.135841000
1	1.720874000	-5.328837000	18.949881000
1	-5.425667000	-3.204466000	18.291669000
1	-6.122063000	-0.695291000	19.088316000
1	4.213738000	-1.315689000	21.205804000
1	3.516924000	1.246727000	21.864494000
1	-5.089540000	-8.126793000	16.774155000
1	-4.431303000	-5.188351000	19.859895000
1	-3.460148000	-6.931247000	15.322859000
1	-5.592676000	-7.236765000	19.064094000
6	-0.511150000	-0.265069000	17.757793000
8	-1.553328000	0.343417000	17.173044000
8	0.489992000	-0.554736000	17.108211000
1	-1.542130000	0.180963000	16.186391000
8	-1.619163000	-0.229232000	14.690815000
8	-1.030733000	-2.182653000	13.773308000
6	-1.680959000	-1.515217000	14.673556000
8	-2.387667000	-2.146942000	15.550102000
6	1.033441000	-4.817951000	14.139985000
6	1.669087000	-3.775030000	13.420455000
6	1.752466000	-6.016489000	14.350238000
6	2.964356000	-3.930761000	12.932604000
6	3.053404000	-6.151440000	13.856634000
6	3.674070000	-5.120489000	13.144885000
1	1.108417000	-2.848923000	13.272818000
1	1.272045000	-6.822822000	14.897502000
1	3.429483000	-3.108110000	12.381544000
1	3.589567000	-7.088619000	14.033702000
1	4.690647000	-5.239550000	12.762541000

^{3}RC2$_{III}$:

26	0.153835000	1.392697000	1.518399000
8	0.125244000	-4.266007000	-2.391549000
7	0.592295000	-0.526768000	1.157670000
7	1.975291000	1.636127000	2.479829000
7	-1.459149000	-2.627133000	-2.118937000
7	0.534322000	-2.094804000	-3.087802000
7	-0.383052000	3.203129000	2.189375000
7	-1.817070000	0.933871000	1.163336000
6	0.417422000	4.150506000	2.792363000
6	-0.240324000	-1.536398000	0.713720000
6	-2.853698000	1.833764000	1.175890000
6	3.029123000	0.767130000	2.408503000
6	-2.347905000	-0.257619000	0.730268000

6	-0.321803000	5.384703000	2.987000000
1	3.884506000	-1.104330000	1.856932000
6	-1.612132000	3.798647000	2.026829000
6	-1.579413000	5.167297000	2.508559000
6	-2.756577000	3.175217000	1.545850000
6	0.519650000	-2.761858000	0.529656000
6	1.842919000	-1.097478000	1.288179000
1	-3.667700000	3.774500000	1.492305000
1	2.240749000	4.817989000	3.654814000
6	1.808840000	-2.483034000	0.866353000
6	-1.624564000	-1.442594000	0.515859000
6	2.971752000	-0.505182000	1.837398000
6	-3.764625000	-0.108922000	0.485578000
6	2.463420000	2.787029000	3.039404000
6	-2.225301000	-3.285332000	-1.134399000
6	-2.372077000	-2.684919000	0.137287000
6	1.734361000	3.968610000	3.192035000
6	-4.076819000	1.194905000	0.758890000
6	4.212185000	1.363416000	2.982340000
6	3.857662000	2.627049000	3.381192000
6	-3.641658000	-5.135457000	-0.439404000
6	-0.234136000	-3.095144000	-2.531758000
6	-3.195412000	-3.314163000	1.085388000
6	-2.850865000	-4.510703000	-1.404703000
6	-3.827797000	-4.527574000	0.806439000
1	-1.740074000	-1.643996000	-2.336032000
1	0.187422000	-1.117031000	-2.937697000
1	0.086279000	6.284225000	3.446315000
1	-2.428238000	5.849960000	2.491415000
1	0.112511000	-3.703502000	0.175036000
1	2.668659000	-3.151805000	0.864485000
1	-4.420910000	-0.897717000	0.127204000
1	-5.043663000	1.690657000	0.679642000
1	5.184044000	0.876687000	3.057998000
1	4.478254000	3.390048000	3.849753000
1	-4.123979000	-6.089904000	-0.664282000
1	-3.316765000	-2.848158000	2.065984000
1	-2.712808000	-4.958703000	-2.389571000
1	-4.454739000	-5.002966000	1.564506000
6	1.017197000	2.230946000	-0.271564000
8	0.257850000	3.178144000	-1.013310000
8	2.166055000	2.112202000	-0.669690000
1	0.858308000	3.485466000	-1.725272000
8	-1.901326000	1.909497000	-2.078286000
8	-0.264313000	0.465035000	-2.585151000
6	-1.504736000	0.677913000	-2.495037000
8	-2.418464000	-0.139211000	-2.755688000
6	1.856593000	-2.185802000	-3.514448000
6	2.563467000	-0.972205000	-3.676909000
6	2.520759000	-3.396895000	-3.799221000
6	3.886485000	-0.973499000	-4.114677000
6	3.848762000	-3.379384000	-4.234357000
6	4.543809000	-2.177193000	-4.398487000
1	2.054076000	-0.036166000	-3.437906000
1	1.988276000	-4.334597000	-3.662280000
1	4.412271000	-0.021695000	-4.229782000
1	4.346826000	-4.329044000	-4.449380000
1	5.581765000	-2.176785000	-4.739072000
1	-1.106301000	2.384874000	-1.736714000

^{3}TS2$_{III}$:

26	-0.853522000	-1.015376000	19.546610000
8	-0.794534000	-6.606706000	15.517069000
7	-0.407940000	-2.961758000	19.235782000
7	0.986246000	-0.711049000	20.375538000
7	-2.308215000	-4.921934000	15.908328000
7	-0.332058000	-4.417993000	14.904578000
7	-1.376535000	0.840733000	20.142172000
7	-2.776095000	-1.445246000	19.110437000
6	-0.566032000	1.809280000	20.689962000
6	-1.235305000	-3.964700000	18.780491000
6	-3.812956000	-0.542271000	19.096076000
6	2.035836000	-1.594872000	20.352276000
6	-3.311237000	-2.645036000	18.692122000
6	-1.297734000	3.047856000	20.845508000
1	2.895798000	-3.485506000	19.898994000
6	-2.599251000	1.433993000	19.952531000
6	-2.562046000	2.814718000	20.383395000
6	-3.734362000	0.797985000	19.459589000
6	-0.481135000	-5.190349000	18.611146000
6	0.841763000	-3.524440000	19.363663000
1	-4.647136000	1.391230000	19.380297000
1	1.285451000	2.495644000	21.483327000
6	0.808225000	-4.911609000	18.961266000
6	-2.611161000	-3.844548000	18.529609000
6	1.978300000	-2.895268000	19.861324000
6	-4.723367000	-2.488936000	18.411900000
6	1.482478000	0.454443000	20.907755000
6	-3.122206000	-5.625657000	16.811102000
6	-3.350435000	-5.069573000	18.091479000
6	0.764449000	1.637544000	21.055142000
6	-5.033707000	-1.184922000	18.663907000
6	3.221944000	-0.981677000	20.908000000
6	2.877029000	0.294319000	21.257226000
6	-4.568009000	-7.499898000	17.369963000
6	-1.117877000	-5.416800000	15.439466000
6	-4.223177000	-5.725104000	18.973028000
6	-3.723710000	-6.847213000	16.470466000
6	-4.833249000	-6.931227000	18.620364000
1	-2.565582000	-3.902523000	15.726512000
1	-0.638923000	-3.421754000	15.102310000
1	-0.882894000	3.965216000	21.260806000
1	-3.406624000	3.501150000	20.339311000
1	-0.886675000	-6.130343000	18.249566000
1	1.667905000	-5.580311000	18.964451000
1	-5.375586000	-3.281579000	18.055790000
1	-5.995980000	-0.684522000	18.564185000
1	4.186852000	-1.476547000	21.010897000
1	3.497898000	1.068206000	21.706361000
1	-5.031345000	-8.449061000	17.089039000
1	-4.401742000	-5.289119000	19.958836000
1	-3.522976000	-7.270132000	15.485662000
1	-5.502579000	-7.431449000	19.324177000
6	0.178545000	-0.384689000	17.667205000
8	-0.893973000	1.085487000	16.656561000
8	1.234731000	-0.369997000	17.198592000
1	-0.238671000	1.231931000	15.958538000
8	-2.594238000	-0.323739000	15.658077000
8	-0.975797000	-1.860960000	15.447953000
6	-2.222001000	-1.573940000	15.482177000
8	-3.129375000	-2.456355000	15.346049000
6	0.936185000	-4.541204000	14.350016000
6	1.621117000	-3.339518000	14.049527000
6	1.574646000	-5.767221000	14.062932000
6	2.894596000	-3.367031000	13.485418000
6	2.852282000	-5.775028000	13.496187000
6	3.525596000	-4.585366000	13.201884000
1	1.125485000	-2.393666000	14.278511000
1	1.059713000	-6.694274000	14.300883000
1	3.402166000	-2.423578000	13.265657000
1	3.328919000	-6.736213000	13.282810000
1	4.524813000	-4.605361000	12.760696000
1	-1.742271000	0.341015000	16.108536000

^{3}IM2$_{III}$:

26	0.054075000	1.229567000	1.744094000
8	0.217197000	-4.104895000	-2.385531000
7	0.462438000	-0.731778000	1.540122000
7	1.925464000	1.570005000	2.452253000
7	-1.300489000	-2.457532000	-1.858869000
7	0.655384000	-1.874641000	-2.852545000
7	-0.391490000	3.163051000	2.094996000
7	-1.867974000	0.834789000	1.309213000
6	0.468884000	4.166303000	2.484738000
6	-0.373416000	-1.721992000	1.074788000
6	-2.893305000	1.750848000	1.276934000
6	2.940203000	0.647755000	2.546307000
6	-2.420543000	-0.365664000	0.915012000
6	-0.225406000	5.433535000	2.531887000
1	3.732640000	-1.310782000	2.313457000
6	-1.612086000	3.765264000	1.908285000
6	-1.520421000	5.183266000	2.175800000
6	-2.783115000	3.110881000	1.542318000
6	0.349690000	-2.972843000	0.973010000
6	1.689305000	-1.325644000	1.735714000
1	-3.688493000	3.713493000	1.451901000
1	2.368661000	4.881361000	3.122267000
6	1.627660000	-2.724770000	1.383558000
6	-1.734820000	-1.574064000	0.770431000
6	2.836817000	-0.696584000	2.206211000
6	-3.832323000	-0.198587000	0.643691000
6	2.475803000	2.776410000	2.818795000
6	-2.141637000	-3.241278000	-1.057963000
6	-2.451555000	-2.774741000	0.242262000
6	1.806141000	3.995775000	2.822003000
6	-4.127587000	1.111202000	0.881022000
6	4.153422000	1.281081000	3.010652000
6	3.865482000	2.606954000	3.178139000
6	-3.579860000	-5.168876000	-0.693662000
6	-0.117536000	-2.914114000	-2.374970000
6	-3.355924000	-3.494835000	1.034587000
6	-2.700571000	-4.449649000	-1.505126000
6	-3.924072000	-4.685531000	0.573539000
1	-1.576979000	-1.413071000	-1.988257000
1	0.337120000	-0.888335000	-2.606084000
1	0.232506000	6.380837000	2.812704000
1	-2.351744000	5.882884000	2.100713000
1	-0.071194000	-3.909016000	0.618769000
1	2.464855000	-3.418262000	1.448602000
1	-4.495277000	-0.990673000	0.306520000
1	-5.085094000	1.620571000	0.781716000
1	5.098180000	0.764913000	3.175791000
1	4.522538000	3.409082000	3.510990000
1	-4.009060000	-6.106042000	-1.057442000
1	-3.592046000	-3.126050000	2.035741000
1	-2.438132000	-4.809973000	-2.500097000
1	-4.620374000	-5.240684000	1.206399000
6	1.209084000	1.486035000	-0.358412000
8	0.220918000	3.858764000	-1.672421000
8	2.264404000	1.501194000	-0.793351000
1	0.968583000	3.426639000	-2.104514000
8	-1.672166000	2.149687000	-2.157558000
8	-0.006552000	0.649617000	-2.222503000
6	-1.281814000	0.934057000	-2.220971000
8	-2.134734000	-0.027964000	-2.296349000
6	1.889975000	-1.951917000	-3.483139000
6	2.510810000	-0.725076000	-3.822314000
6	2.557015000	-3.152986000	-3.810936000
6	3.748649000	-0.704750000	-4.460521000
6	3.797643000	-3.112302000	-4.453783000
6	4.407708000	-1.898325000	-4.784261000
1	1.989506000	0.199684000	-3.565910000
1	2.089765000	-4.098009000	-3.546546000
1	4.205662000	0.257329000	-4.709273000
1	4.296323000	-4.054927000	-4.697926000
1	5.378804000	-1.880494000	-5.284520000
1	-0.517453000	3.182779000	-1.849518000

^{5}RC1$_{III}$:

26	0.008010000	1.569859000	1.827046000
8	0.163060000	-4.383614000	-2.343407000
7	0.546410000	-0.415104000	1.457784000
7	1.860184000	1.871320000	2.802156000
7	-1.096386000	-2.498170000	-1.967984000
7	0.693292000	-2.398457000	-3.398268000
7	-0.666715000	3.339778000	2.704419000
7	-2.001767000	0.981144000	1.527979000
6	0.113862000	4.301135000	3.310318000
6	-0.245063000	-1.417276000	0.931589000
6	-3.073596000	1.844887000	1.569532000
6	2.947867000	1.039240000	2.709885000
6	-2.438739000	-0.191377000	0.949536000
6	-0.709075000	5.472584000	3.596771000
1	3.858588000	-0.818994000	2.156469000
6	-1.944123000	3.842950000	2.604643000
6	-1.971477000	5.191400000	3.162544000
6	-3.039280000	3.171530000	2.081863000
6	0.583888000	-2.601318000	0.704408000
6	1.824867000	-0.916819000	1.589285000
1	-3.986698000	3.716957000	2.064825000
1	1.939062000	5.036879000	4.094850000
6	1.850160000	-2.289063000	1.098344000
6	-1.619424000	-1.343431000	0.692265000
6	2.916746000	-0.263841000	2.142417000
6	-3.826785000	-0.068035000	0.621279000
6	2.289117000	3.045656000	3.378359000
6	-2.016694000	-3.118695000	-1.105428000
6	-2.310244000	-2.557468000	0.162470000
6	1.461016000	4.177062000	3.618241000
6	-4.223737000	1.207474000	1.014132000
6	4.097969000	1.687739000	3.260235000
6	3.683948000	2.949793000	3.680165000
6	-3.609848000	-4.919901000	-0.692493000
6	-0.062895000	-3.189270000	-2.556787000
6	-3.280524000	-3.195041000	0.959022000
6	-2.670401000	-4.297769000	-1.511087000
6	-3.925974000	-4.361793000	0.551835000
1	-1.045988000	-1.451427000	-1.973471000
1	0.332351000	-1.449836000	-3.643662000
1	-0.350442000	6.382833000	4.078077000
1	-2.857010000	5.825417000	3.216744000
1	0.235808000	-3.542901000	0.287070000
1	2.733624000	-2.927918000	1.078851000
1	-4.436733000	-0.834768000	0.149226000
1	-5.212535000	1.657894000	0.921356000
1	5.096781000	1.254611000	3.323942000
1	4.283136000	3.731033000	4.148809000
1	-4.103652000	-5.834669000	-1.030426000
1	-3.510543000	-2.759508000	1.933901000
1	-2.428854000	-4.718220000	-2.487389000
1	-4.663139000	-4.837816000	1.203136000
6	0.527715000	2.403250000	0.009143000
8	-0.435667000	2.969425000	-0.539030000
8	1.693602000	2.198158000	-0.307153000
1	-0.382141000	2.434460000	-2.170800000
8	-0.179567000	2.111865000	-3.088183000
8	0.013376000	0.177209000	-4.163025000

6	-0.371917000	0.764110000	-3.118970000
8	-0.906521000	0.227233000	-2.117669000
6	1.912568000	-2.720437000	-3.995329000
6	2.528712000	-1.714877000	-4.776492000
6	2.568072000	-3.962802000	-3.861096000
6	3.752114000	-1.948583000	-5.400994000
6	3.797198000	-4.176860000	-4.491693000
6	4.401586000	-3.182070000	-5.265495000
1	2.020902000	-0.752847000	-4.871191000
1	2.098302000	-4.742051000	-3.267723000
1	4.205735000	-1.153667000	-5.999539000
1	4.287780000	-5.147285000	-4.373587000
1	5.361248000	-3.362928000	-5.755201000

^{5}TS1$_{III}$:

26	-0.793457000	-0.666678000	19.493295000
8	-0.698873000	-6.627636000	15.695071000
7	-0.381327000	-2.788126000	19.419900000
7	0.988007000	-0.485735000	20.663963000
7	-2.047578000	-4.783834000	15.953309000
7	-0.260648000	-4.676017000	14.532300000
7	-1.527699000	1.004513000	20.617260000
7	-2.864039000	-1.334916000	19.436052000
6	-0.740051000	1.942347000	21.225287000
6	-1.171847000	-3.771454000	18.891639000
6	-3.920180000	-0.465790000	19.495481000
6	2.039980000	-1.354728000	20.637992000
6	-3.321410000	-2.492655000	18.868470000
6	-1.543070000	3.107183000	21.565992000
1	2.915428000	-3.232886000	20.154783000
6	-2.794768000	1.508906000	20.563086000
6	-2.821254000	2.836606000	21.155465000
6	-3.897064000	0.835306000	20.021379000
6	-0.377909000	-4.976669000	18.685219000
6	0.876767000	-3.309039000	19.564724000
1	-4.846847000	1.375740000	20.015639000
1	1.115217000	2.637468000	22.003173000
6	0.892640000	-4.685097000	19.096245000
6	-2.553123000	-3.652650000	18.615288000
6	1.989262000	-2.654243000	20.112385000
6	-4.735803000	-2.350668000	18.547222000
6	1.419850000	0.662823000	21.264679000
6	-2.959349000	-5.413589000	16.813233000
6	-3.260710000	-4.856417000	18.079916000
6	0.625620000	1.794662000	21.509068000
6	-5.105472000	-1.093906000	18.940042000
6	3.203591000	-0.734073000	21.247523000
6	2.818300000	0.522748000	21.636353000
6	-4.541874000	-7.223458000	17.234216000
6	-0.980764000	-5.458178000	15.408842000
6	-4.226032000	-5.492501000	18.881859000
6	-3.605290000	-6.598876000	16.413844000
6	-4.865336000	-6.663389000	18.475208000
1	-2.087061000	-3.722192000	15.844218000
1	-0.693075000	-3.761932000	14.209490000
1	-1.171130000	4.004321000	22.060593000
1	-3.705197000	3.467457000	21.248278000
1	-0.739133000	-5.910443000	18.262301000
1	1.765036000	-5.338398000	19.093235000
1	-5.356868000	-3.111760000	18.081402000
1	-6.087471000	-0.627522000	18.862967000
1	4.180477000	-1.203376000	21.363112000
1	3.417003000	1.285896000	22.133324000
1	-5.029487000	-8.142636000	16.899390000
1	-4.454898000	-5.059741000	19.858333000
1	-3.360223000	-7.020509000	15.438517000
1	-5.602309000	-7.140579000	19.125411000
6	-0.296940000	-0.014688000	17.518375000
8	-1.270785000	0.569367000	16.840094000
8	0.751427000	-0.370032000	16.933149000
1	-1.313627000	0.140844000	15.670580000
8	-1.370089000	-0.309228000	14.576545000
8	-1.141373000	-2.310837000	13.629090000
6	-1.554810000	-1.611584000	14.618671000
8	-2.131246000	-2.135632000	15.627482000
6	0.993598000	-4.945294000	13.992471000
6	1.554835000	-3.950422000	13.155757000
6	1.742393000	-6.117584000	14.234631000
6	2.813493000	-4.126198000	12.585406000
6	3.005747000	-6.273686000	13.656642000
6	3.555216000	-5.289541000	12.829575000
1	0.970380000	-3.044110000	12.981118000
1	1.316096000	-6.888962000	14.870159000
1	3.223083000	-3.340955000	11.943552000
1	3.568667000	-7.189496000	13.859340000
1	4.542839000	-5.424753000	12.382416000

^{5}IM1$_{III}$:

26	0.108781000	1.649312000	1.592727000
8	0.201105000	-4.303890000	-2.204770000
7	0.540903000	-0.451430000	1.436589000
7	1.935654000	1.843518000	2.668235000
7	-1.141594000	-2.447558000	-1.999438000
7	0.696099000	-2.352848000	-3.349712000
7	-0.591199000	3.318180000	2.723746000
7	-1.946914000	0.990318000	1.540192000
6	0.209405000	4.249911000	3.324898000
6	-0.259978000	-1.431434000	0.915740000
6	-3.003145000	1.856366000	1.636462000
6	2.990823000	0.980220000	2.599815000
6	-2.410145000	-0.154634000	0.949893000
6	-0.590009000	5.403192000	3.708833000
1	3.858386000	-0.890170000	2.075903000
6	-1.862372000	3.816254000	2.714298000
6	-1.878168000	5.132658000	3.330875000
6	-2.974132000	3.146406000	2.188720000
6	0.530939000	-2.633616000	0.685471000
6	1.803556000	-0.969981000	1.546480000
1	-3.926244000	3.681956000	2.214965000
1	2.083685000	4.945388000	4.055210000
6	1.809996000	-2.342418000	1.068407000
6	-1.645359000	-1.309669000	0.663189000
6	2.929427000	-0.314721000	2.065130000
6	-3.827696000	-0.004519000	0.649550000
6	2.377969000	2.986044000	3.274257000
6	-2.062636000	-3.066239000	-1.143752000
6	-2.362605000	-2.505900000	0.123388000
6	1.584348000	4.107559000	3.562589000
6	-4.194032000	1.240467000	1.080907000
6	4.167767000	1.599599000	3.183315000
6	3.787082000	2.848401000	3.601511000
6	-3.666069000	-4.860726000	-0.722560000
6	-0.063798000	-3.130615000	-2.503810000
6	-3.334775000	-3.132844000	0.924832000
6	-2.721249000	-4.246091000	-1.540987000
6	-3.985441000	-4.297353000	0.518090000
1	-1.209398000	-1.370074000	-2.160577000
1	0.284740000	-1.414247000	-3.683559000
1	-0.208497000	6.293803000	4.207793000
1	-2.762153000	5.756837000	3.460231000
1	0.163696000	-3.564640000	0.262437000
1	2.683392000	-2.993607000	1.038962000
1	-4.453364000	-0.752588000	0.169398000
1	-5.177053000	1.707791000	1.027177000

1	5.149943000	1.134241000	3.264040000
1	4.395731000	3.607485000	4.092443000
1	-4.162347000	-5.775337000	-1.057478000
1	-3.559875000	-2.698976000	1.901690000
1	-2.477264000	-4.671373000	-2.515133000
1	-4.727525000	-4.766998000	1.168028000
6	0.503312000	2.273606000	-0.410131000
8	-0.560259000	2.815264000	-1.053108000
8	1.491316000	1.938810000	-1.078152000
1	-0.564085000	2.533608000	-2.054533000
8	-0.706434000	1.980934000	-3.471880000
8	-0.126957000	-0.026010000	-4.267769000
6	-0.729879000	0.697316000	-3.378445000
8	-1.353005000	0.123925000	-2.403005000
6	1.963952000	-2.635751000	-3.841276000
6	2.576979000	-1.634796000	-4.635871000
6	2.685505000	-3.825578000	-3.594256000
6	3.853626000	-1.821877000	-5.160197000
6	3.967690000	-3.992546000	-4.125786000
6	4.566076000	-3.002907000	-4.911548000
1	2.013660000	-0.715499000	-4.813021000
1	2.221730000	-4.600549000	-2.990028000
1	4.301524000	-1.031500000	-5.769630000
1	4.506821000	-4.922003000	-3.919575000
1	5.567952000	-3.146972000	-5.322952000

^{5}RC2$_{III}$:

26	0.204981000	1.453660000	1.310468000
8	0.125685000	-4.267820000	-2.355498000
7	0.607520000	-0.618896000	1.159419000
7	2.006645000	1.638518000	2.422515000
7	-1.466989000	-2.632148000	-2.115843000
7	0.545319000	-2.096480000	-3.044585000
7	-0.430550000	3.253089000	2.248765000
7	-1.843260000	0.950098000	1.095907000
6	0.390087000	4.179912000	2.831960000
6	-0.248448000	-1.601453000	0.732949000
6	-2.884407000	1.837340000	1.180213000
6	3.042796000	0.749317000	2.383469000
6	-2.359802000	-0.252667000	0.685117000
6	-0.360902000	5.398868000	3.089968000
1	3.874135000	-1.160736000	1.949381000
6	-1.668947000	3.816304000	2.132045000
6	-1.638721000	5.172810000	2.655356000
6	-2.799723000	3.165110000	1.621281000
6	0.481680000	-2.856948000	0.624139000
6	1.835697000	-1.196655000	1.355349000
1	-3.729457000	3.738729000	1.608845000
1	2.265516000	4.819621000	3.603930000
6	1.771567000	-2.601302000	0.997917000
6	-1.634937000	-1.449727000	0.502411000
6	2.964179000	-0.559522000	1.884796000
6	-3.792831000	-0.115079000	0.486562000
6	2.483069000	2.802964000	2.960343000
6	-2.243757000	-3.289435000	-1.138382000
6	-2.396946000	-2.686840000	0.130676000
6	1.738491000	3.977047000	3.150233000
6	-4.115156000	1.178425000	0.791324000
6	4.232521000	1.364524000	2.942469000
6	3.885387000	2.640640000	3.300850000
6	-3.668275000	-5.134062000	-0.448993000
6	-0.231984000	-3.097574000	-2.502772000
6	-3.225381000	-3.311874000	1.076423000
6	-2.870575000	-4.513255000	-1.411409000
6	-3.859294000	-4.524174000	0.795211000
1	-1.739850000	-1.644721000	-2.323451000
1	0.194690000	-1.118820000	-2.905397000
1	0.043100000	6.297005000	3.556129000
1	-2.490976000	5.850956000	2.689843000
1	0.063617000	-3.802453000	0.291848000
1	2.605555000	-3.300519000	1.049695000
1	-4.456591000	-0.908117000	0.151890000
1	-5.096458000	1.651102000	0.762946000
1	5.202157000	0.877692000	3.043494000
1	4.512070000	3.405816000	3.757923000
1	-4.151695000	-6.087772000	-0.674628000
1	-3.349412000	-2.844892000	2.056247000
1	-2.727921000	-4.963646000	-2.394571000
1	-4.490960000	-4.997553000	1.550618000
6	1.052625000	2.348627000	-0.417105000
8	0.273810000	3.285904000	-1.144097000
8	2.204129000	2.246315000	-0.817307000
1	0.864739000	3.620451000	-1.851618000
8	-1.887671000	1.952325000	-2.163657000
8	-0.260631000	0.468272000	-2.582285000
6	-1.499401000	0.694183000	-2.507596000
8	-2.419479000	-0.127761000	-2.722735000
6	1.867453000	-2.188889000	-3.471170000
6	2.570826000	-0.975370000	-3.649203000
6	2.534567000	-3.401452000	-3.742586000
6	3.893241000	-0.978387000	-4.088725000
6	3.861798000	-3.385486000	-4.180122000
6	4.553446000	-2.183532000	-4.359399000
1	2.059173000	-0.037536000	-3.422529000
1	2.004587000	-4.338796000	-3.594091000
1	4.416021000	-0.026517000	-4.216266000
1	4.361960000	-4.336325000	-4.384821000
1	5.590830000	-2.184350000	-4.701699000
1	-1.089113000	2.444729000	-1.860930000

^{5}TS2$_{III}$:

26	-0.789880000	-0.986888000	19.329029000
8	-0.748109000	-6.590268000	15.509240000
7	-0.400769000	-3.051906000	19.302348000
7	1.018793000	-0.743201000	20.419118000
7	-2.272562000	-4.922488000	15.938624000
7	-0.305936000	-4.383996000	14.941790000
7	-1.381321000	0.898306000	20.068634000
7	-2.818204000	-1.438946000	19.040149000
6	-0.547163000	1.853391000	20.585881000
6	-1.250239000	-4.026601000	18.846246000
6	-3.846299000	-0.534185000	19.046242000
6	2.034545000	-1.657459000	20.480618000
6	-3.334685000	-2.649420000	18.652044000
6	-1.275514000	3.101622000	20.735219000
1	2.839420000	-3.604173000	20.185794000
6	-2.607071000	1.474756000	19.891173000
6	-2.553744000	2.865954000	20.306374000
6	-3.746337000	0.813436000	19.413423000
6	-0.531215000	-5.289375000	18.777521000
6	0.815105000	-3.638370000	19.542627000
1	-4.662274000	1.404694000	19.346335000
1	1.325706000	2.517868000	21.341211000
6	0.745684000	-5.046007000	19.203260000
6	-2.619979000	-3.857563000	18.539192000
6	1.941487000	-2.993533000	20.067736000
6	-4.755174000	-2.498650000	18.387889000
6	1.510905000	0.450596000	20.876066000
6	-3.092712000	-5.641199000	16.821685000
6	-3.355756000	-5.084343000	18.095091000
6	0.790196000	1.651012000	20.947652000
6	-5.072530000	-1.193248000	18.639715000
6	3.222231000	-1.025816000	21.023854000
6	2.897598000	0.282367000	21.269133000

6	-4.525732000	-7.531418000	17.356225000
6	-1.080806000	-5.401290000	15.458898000
6	-4.239358000	-5.746187000	18.959852000
6	-3.670135000	-6.872002000	16.472061000
6	-4.826860000	-6.961208000	18.597878000
1	-2.536560000	-3.899464000	15.766908000
1	-0.614156000	-3.392732000	15.171861000
1	-0.857618000	4.026233000	21.131845000
1	-3.392554000	3.560622000	20.276994000
1	-0.948460000	-6.232013000	18.434864000
1	1.569128000	-5.753626000	19.292917000
1	-5.415143000	-3.295146000	18.054050000
1	-6.044960000	-0.709204000	18.556443000
1	4.176061000	-1.525029000	21.191233000
1	3.531151000	1.067232000	21.680766000
1	-4.970091000	-8.488000000	17.069625000
1	-4.442668000	-5.310238000	19.940956000
1	-3.441497000	-7.296823000	15.494259000
1	-5.505072000	-7.467820000	19.288551000
6	0.300039000	-0.379970000	17.603892000
8	-0.838011000	1.069531000	16.682279000
8	1.341623000	-0.334421000	17.107008000
1	-0.242888000	1.239121000	15.936882000
8	-2.584345000	-0.330306000	15.761507000
8	-0.952891000	-1.852076000	15.560706000
6	-2.206123000	-1.567569000	15.575034000
8	-3.098485000	-2.463563000	15.404026000
6	0.933115000	-4.486505000	14.323214000
6	1.579094000	-3.273147000	13.984415000
6	1.578044000	-5.701522000	14.004653000
6	2.820202000	-3.278860000	13.351937000
6	2.822219000	-5.687274000	13.367792000
6	3.456682000	-4.486312000	13.035399000
1	1.076892000	-2.336348000	14.236139000
1	1.093131000	-6.636869000	14.272114000
1	3.297336000	-2.326861000	13.102607000
1	3.304055000	-6.640088000	13.129812000
1	4.430084000	-4.489180000	12.539435000
1	-1.669901000	0.379567000	16.212048000

^{5}IM2$_{III}$:

26	0.089658000	1.302628000	1.258389000
8	0.130005000	-4.117739000	-2.423732000
7	0.486715000	-0.784390000	1.332778000
7	1.986006000	1.609612000	2.171304000
7	-1.383143000	-2.467930000	-1.920038000
7	0.675824000	-1.892820000	-2.733902000
7	-0.424331000	3.221359000	1.993403000
7	-1.949472000	0.786376000	1.298895000
6	0.436693000	4.206521000	2.393691000
6	-0.378015000	-1.774563000	0.960779000
6	-2.974188000	1.693459000	1.360587000
6	3.008388000	0.698901000	2.164704000
6	-2.486557000	-0.421348000	0.931943000
6	-0.303740000	5.439042000	2.615902000
1	3.804975000	-1.253838000	1.898655000
6	-1.682357000	3.757822000	1.976115000
6	-1.617589000	5.160759000	2.358231000
6	-2.853927000	3.056064000	1.672493000
6	0.349340000	-3.034537000	0.858803000
6	1.726498000	-1.346925000	1.474587000
1	-3.783427000	3.629755000	1.689796000
1	2.379725000	4.913783000	2.896951000
6	1.651096000	-2.768071000	1.177770000
6	-1.763940000	-1.616778000	0.737686000
6	2.887926000	-0.662249000	1.847195000
6	-3.919336000	-0.273273000	0.758909000
6	2.522140000	2.825533000	2.498392000
6	-2.231657000	-3.248818000	-1.108097000
6	-2.493551000	-2.811410000	0.211485000
6	1.808376000	4.031251000	2.600424000
6	-4.221677000	1.033080000	1.037532000
6	4.247546000	1.358961000	2.525155000
6	3.945732000	2.680247000	2.731502000
6	-3.682535000	-5.165082000	-0.765600000
6	-0.169384000	-2.925828000	-2.368661000
6	-3.384027000	-3.546881000	1.006154000
6	-2.818913000	-4.430425000	-1.580611000
6	-3.978627000	-4.716755000	0.525837000
1	-1.576259000	-1.441452000	-1.944793000
1	0.396147000	-0.945806000	-2.448926000
1	0.133163000	6.382132000	2.942882000
1	-2.474282000	5.830113000	2.430358000
1	-0.083238000	-3.989694000	0.573435000
1	2.488474000	-3.463826000	1.218677000
1	-4.600814000	-1.066616000	0.461784000
1	-5.198583000	1.515007000	1.016648000
1	5.217063000	0.868888000	2.608890000
1	4.619349000	3.487921000	3.016298000
1	-4.135475000	-6.084413000	-1.144911000
1	-3.588288000	-3.205562000	2.023635000
1	-2.593446000	-4.758773000	-2.595578000
1	-4.661760000	-5.283304000	1.162943000
6	0.772766000	1.396978000	-0.738061000
8	0.193759000	4.234819000	-1.613888000
8	1.915805000	1.603528000	-1.109857000
1	0.988826000	3.694046000	-1.509727000
8	-1.716558000	2.292590000	-1.703838000
8	-0.028743000	0.789689000	-1.794716000
6	-1.391724000	1.106192000	-1.896760000
8	-2.117266000	0.147872000	-2.234995000
6	1.973591000	-2.003833000	-3.242087000
6	2.788933000	-0.852189000	-3.204687000
6	2.496203000	-3.188589000	-3.798955000
6	4.086405000	-0.890052000	-3.714685000
6	3.800014000	-3.207648000	-4.301395000
6	4.606589000	-2.066243000	-4.267548000
1	2.403645000	0.064585000	-2.750282000
1	1.878189000	-4.082196000	-3.818407000
1	4.701334000	0.013215000	-3.671646000
1	4.188020000	-4.136690000	-4.728290000
1	5.624850000	-2.092787000	-4.662396000
1	-0.513226000	3.543634000	-1.638109000

[Fe^{II}(*ortho*-urea-TPP)(phenol)]$^{2-}$

Solvent = water:

^{5}RC1$_{III,phenol}$:

26	-1.156527000	-1.008028000	19.673277000
8	-0.894653000	-5.573290000	15.070751000
7	-0.587345000	-2.989622000	19.315830000
7	0.847800000	-0.514568000	19.986845000
7	-2.354033000	-4.121715000	16.107201000
7	-0.223036000	-3.434408000	15.630669000
7	-1.670735000	0.731064000	20.754765000
7	-3.127965000	-1.661196000	19.820432000
6	-0.829836000	1.762736000	21.084200000
6	-1.429115000	-4.009149000	18.951468000
6	-4.204386000	-0.848615000	20.138669000
6	1.934204000	-1.319188000	19.686685000
6	-3.625984000	-2.818353000	19.274722000
6	-1.597477000	2.874250000	21.566225000
1	2.823040000	-3.150592000	19.120751000
6	-2.961865000	1.166585000	20.982842000

6	-2.933209000	2.498675000	21.501164000
6	-4.128932000	0.421973000	20.688304000
6	-0.653732000	-5.164494000	18.608809000
6	0.708309000	-3.445039000	19.155755000
1	-5.080759000	0.920440000	20.890536000
1	1.120891000	2.638305000	21.238627000
6	0.684057000	-4.805917000	18.733314000
6	-2.859864000	-3.909613000	18.841643000
6	1.871401000	-2.652574000	19.320717000
6	-5.084307000	-2.730171000	19.225683000
6	1.340030000	0.689706000	20.431543000
6	-3.215807000	-5.090226000	16.669068000
6	-3.525130000	-4.985198000	18.050720000
6	0.581583000	1.737177000	20.938506000
6	-5.434013000	-1.529809000	19.776218000
6	3.159776000	-0.561486000	19.893532000
6	2.796328000	0.671868000	20.351510000
6	-4.635099000	-7.046511000	16.477307000
6	-1.125610000	-4.473045000	15.559347000
6	-4.421318000	-5.911669000	18.602613000
6	-3.762565000	-6.121467000	15.896542000
6	-4.976600000	-6.935960000	17.828640000
1	-2.330543000	-3.245326000	16.634645000
1	-0.541295000	-2.615673000	16.160262000
1	-1.183605000	3.824945000	21.902996000
1	-3.809764000	3.085777000	21.776222000
1	-1.056399000	-6.119731000	18.279422000
1	1.562499000	-5.417744000	18.528875000
1	-5.745622000	-3.500905000	18.836268000
1	-6.436455000	-1.127415000	19.923358000
1	4.165152000	-0.947373000	19.724091000
1	3.442981000	1.504649000	20.626805000
1	-5.057538000	-7.848695000	15.867002000
1	-4.663310000	-5.836228000	19.665421000
1	-3.503160000	-6.185239000	14.839867000
1	-5.664720000	-7.652759000	18.283373000
6	-1.496651000	-0.432042000	17.748579000
8	-1.589927000	0.799296000	17.612616000
8	-1.609337000	-1.345514000	16.917713000
6	1.149228000	-3.485654000	15.380323000
6	1.943096000	-2.462766000	15.937806000
6	1.773467000	-4.490615000	14.616842000
6	3.322372000	-2.448255000	15.742375000
6	3.158709000	-4.462516000	14.429858000
6	3.944747000	-3.449166000	14.986488000
1	1.468706000	-1.696389000	16.552596000
1	1.167022000	-5.286540000	14.192529000
1	3.915628000	-1.652526000	16.199088000
1	3.627964000	-5.251598000	13.835966000
1	5.026711000	-3.439383000	14.836842000
6	1.240890000	2.525703000	17.422510000
6	2.336061000	3.365868000	17.698273000
6	3.583675000	3.112794000	17.127221000
6	3.768103000	2.021798000	16.268708000
6	2.678068000	1.191068000	15.986405000
6	1.423980000	1.433082000	16.551935000
1	2.185500000	4.209174000	18.376121000
1	4.423164000	3.773958000	17.359257000
1	4.745772000	1.824032000	15.823613000
1	2.799766000	0.340611000	15.312205000
1	0.576196000	0.785353000	16.324695000
8	0.058021000	2.814212000	17.988655000
1	-0.567061000	2.046916000	17.904576000

^{5}TS1$_{III,phenol}$:

26	-1.085240000	-0.991390000	19.608354000
8	-0.907646000	-5.597719000	15.085736000
7	-0.545728000	-2.982520000	19.262310000
7	0.917731000	-0.538067000	19.939667000
7	-2.381059000	-4.134624000	16.084945000
7	-0.250766000	-3.443994000	15.606313000
7	-1.574277000	0.752870000	20.681601000
7	-3.063698000	-1.607476000	19.755955000
6	-0.714809000	1.751363000	21.065226000
6	-1.404033000	-3.998478000	18.921186000
6	-4.129981000	-0.767509000	20.038759000
6	1.993160000	-1.350956000	19.617906000
6	-3.577850000	-2.767114000	19.226211000
6	-1.462758000	2.866963000	21.564120000
1	2.859252000	-3.184583000	19.025941000
6	-2.859306000	1.219563000	20.892894000
6	-2.806132000	2.534141000	21.449505000
6	-4.037982000	0.506947000	20.573720000
6	-0.647214000	-5.165898000	18.582757000
6	0.741620000	-3.456378000	19.088198000
1	-4.981393000	1.027766000	20.756938000
1	1.252145000	2.574078000	21.275898000
6	0.696265000	-4.820544000	18.681899000
6	-2.832555000	-3.880476000	18.820339000
6	1.915015000	-2.677854000	19.237554000
6	-5.032319000	-2.652707000	19.157769000
6	1.432314000	0.647759000	20.413403000
6	-3.231913000	-5.096436000	16.676381000
6	-3.518681000	-4.966072000	18.059919000
6	0.695572000	1.695162000	20.944418000
6	-5.366101000	-1.432911000	19.673528000
6	3.229890000	-0.616803000	19.839124000
6	2.886753000	0.607622000	20.332345000
6	-4.650736000	-7.056457000	16.544555000
6	-1.146523000	-4.490060000	15.551439000
6	-4.406396000	-5.880010000	18.644293000
6	-3.788368000	-6.142332000	15.931987000
6	-4.972487000	-6.919147000	17.898290000
1	-2.362785000	-3.252112000	16.599906000
1	-0.579918000	-2.616459000	16.109570000
1	-1.030538000	3.794285000	21.940206000
1	-3.673141000	3.136547000	21.721210000
1	-1.065454000	-6.119917000	18.270421000
1	1.565132000	-5.443505000	18.471196000
1	-5.702056000	-3.421705000	18.779580000
1	-6.362082000	-1.007983000	19.798451000
1	4.228135000	-1.014650000	19.656657000
1	3.545363000	1.423297000	20.628327000
1	-5.081614000	-7.870971000	15.957006000
1	-4.632942000	-5.783037000	19.708792000
1	-3.545270000	-6.226081000	14.872722000
1	-5.653888000	-7.626717000	18.376868000
6	-1.483472000	-0.426213000	17.720723000
8	-1.617229000	0.815081000	17.475073000
8	-1.668535000	-1.344632000	16.923772000
6	1.127426000	-3.487063000	15.383055000
6	1.888179000	-2.399299000	15.857468000
6	1.787496000	-4.545574000	14.730804000
6	3.270859000	-2.373880000	15.691589000
6	3.176176000	-4.506125000	14.573114000
6	3.929712000	-3.428821000	15.048680000
1	1.387257000	-1.584343000	16.382328000
1	1.205590000	-5.389775000	14.370499000
1	3.836170000	-1.524826000	16.082744000
1	3.674177000	-5.338182000	14.067900000
1	5.014576000	-3.410837000	14.922615000
6	1.031127000	2.403650000	17.496632000
6	2.093964000	3.247414000	17.917599000

6	3.379454000	3.115245000	17.396970000
6	3.670433000	2.139013000	16.432803000
6	2.634023000	1.304376000	15.996475000
6	1.340830000	1.427919000	16.508240000
1	1.871571000	4.000295000	18.677998000
1	4.172014000	3.781162000	17.752644000
1	4.679797000	2.035293000	16.027735000
1	2.831583000	0.543093000	15.236736000
1	0.545969000	0.774090000	16.144772000
8	-0.169124000	2.554857000	18.006350000
1	-0.903122000	1.641431000	17.800219000

^{5}**IM1**$_{III,phenol}$:

26	-1.005993000	-0.945365000	19.261796000
8	-1.032100000	-5.717239000	15.141674000
7	-0.508157000	-3.020154000	19.138827000
7	1.026546000	-0.645861000	19.904609000
7	-2.549642000	-4.225557000	16.037236000
7	-0.476023000	-3.490116000	15.433370000
7	-1.453267000	0.723899000	20.546116000
7	-2.988908000	-1.644198000	19.762444000
6	-0.572645000	1.703503000	20.915514000
6	-1.383751000	-4.016958000	18.818442000
6	-4.026929000	-0.805382000	20.058983000
6	2.069754000	-1.490744000	19.638652000
6	-3.528097000	-2.796169000	19.258031000
6	-1.302030000	2.857478000	21.414026000
1	2.874359000	-3.357551000	19.015263000
6	-2.711319000	1.202820000	20.782197000
6	-2.632382000	2.548853000	21.326991000
6	-3.902779000	0.503727000	20.544334000
6	-0.642547000	-5.220468000	18.470105000
6	0.756801000	-3.523308000	18.990187000
1	-4.833075000	1.037866000	20.751798000
1	1.389123000	2.478657000	21.188667000
6	0.685718000	-4.911344000	18.577289000
6	-2.795472000	-3.910947000	18.803607000
6	1.945006000	-2.813424000	19.196992000
6	-4.981143000	-2.687106000	19.240830000
6	1.561635000	0.510677000	20.398996000
6	-3.342974000	-5.174463000	16.720637000
6	-3.529083000	-5.017622000	18.116522000
6	0.821041000	1.611025000	20.847706000
6	-5.289229000	-1.456563000	19.753583000
6	3.327029000	-0.832847000	19.948477000
6	3.010092000	0.410319000	20.428002000
6	-4.733844000	-7.160301000	16.750768000
6	-1.316471000	-4.579481000	15.497190000
6	-4.354363000	-5.924120000	18.794463000
6	-3.937029000	-6.250874000	16.050952000
6	-4.955614000	-6.991838000	18.121271000
1	-2.547550000	-3.286382000	16.455806000
1	-0.875939000	-2.626426000	15.829225000
1	-0.846158000	3.777252000	21.779849000
1	-3.487142000	3.162415000	21.610872000
1	-1.082938000	-6.168812000	18.173223000
1	1.543154000	-5.555860000	18.387376000
1	-5.667711000	-3.452949000	18.887361000
1	-6.276018000	-1.018399000	19.901317000
1	4.314465000	-1.277860000	19.828728000
1	3.686087000	1.192338000	20.772202000
1	-5.193530000	-7.997302000	16.219521000
1	-4.501120000	-5.800477000	19.869915000
1	-3.773629000	-6.360474000	14.978566000
1	-5.586662000	-7.696313000	18.668019000
6	-1.498277000	-0.510503000	17.183938000
8	-1.456097000	0.656803000	16.551413000
8	-2.001656000	-1.448494000	16.511946000
6	0.911230000	-3.496508000	15.281305000
6	1.616066000	-2.380748000	15.777667000
6	1.632194000	-4.546807000	14.683341000
6	3.003536000	-2.315419000	15.677048000
6	3.025477000	-4.469619000	14.594166000
6	3.722524000	-3.360780000	15.084313000
1	1.064764000	-1.583765000	16.279976000
1	1.092621000	-5.414675000	14.311727000
1	3.525538000	-1.447769000	16.087254000
1	3.572699000	-5.294993000	14.130613000
1	4.811310000	-3.312931000	15.010496000
6	1.017119000	2.532911000	17.478606000
6	1.845448000	3.554181000	18.043070000
6	3.216205000	3.612211000	17.800085000
6	3.849810000	2.664090000	16.981438000
6	3.062398000	1.654661000	16.407989000
6	1.689535000	1.582948000	16.642058000
1	1.361046000	4.294011000	18.687317000
1	3.807053000	4.412206000	18.259282000
1	4.925211000	2.712560000	16.794010000
1	3.528902000	0.907494000	15.757148000
1	1.092959000	0.789554000	16.187559000
8	-0.255482000	2.494736000	17.712261000
1	-0.950081000	1.405135000	17.103282000

^{5}**RC2**$_{III,phenol}$:

26	-0.845791000	1.201758000	2.035904000
8	-0.081544000	-2.650986000	-3.984020000
7	0.198888000	-0.609523000	2.331407000
7	0.520070000	2.126021000	3.351983000
7	-0.543080000	-2.054635000	-1.803133000
7	0.313730000	-0.505287000	-3.249736000
7	-2.148211000	2.819334000	2.358870000
7	-2.460447000	0.087110000	1.327488000
6	-1.836972000	4.007269000	2.963678000
6	-0.094901000	-1.788906000	1.703622000
6	-3.660897000	0.611291000	0.929828000
6	1.695870000	1.585949000	3.811632000
6	-2.345565000	-1.161702000	0.777743000
6	-2.975343000	4.911001000	2.871874000
1	3.086177000	-0.008984000	4.022997000
6	-3.434713000	2.915767000	1.900066000
6	-3.964418000	4.234939000	2.215030000
6	-4.124995000	1.904645000	1.223369000
6	0.985605000	-2.740019000	1.939941000
6	1.422513000	-0.749264000	2.928795000
1	-5.128831000	2.150514000	0.869840000
1	-0.522578000	5.296964000	4.025845000
6	1.918044000	-2.098624000	2.704936000
6	-1.244449000	-2.036513000	0.925427000
6	2.114265000	0.262111000	3.604949000
6	-3.543004000	-1.455767000	0.012284000
6	0.457771000	3.422893000	3.795106000
6	-0.902604000	-3.283766000	-1.239219000
6	-1.271063000	-3.305203000	0.133135000
6	-0.623774000	4.297206000	3.597890000
6	-4.362799000	-0.362110000	0.118843000
6	2.421918000	2.589822000	4.559448000
6	1.655408000	3.728158000	4.548464000
6	-1.266083000	-5.688810000	-1.335879000
6	-0.096368000	-1.806968000	-3.091967000
6	-1.631039000	-4.516860000	0.731390000
6	-0.905713000	-4.493680000	-1.960938000
6	-1.633323000	-5.714078000	0.011224000
1	-0.526252000	-1.259902000	-1.163849000
1	0.308318000	0.087659000	-2.411044000

1	-3.003198000	5.923964000	3.271827000
1	-4.967229000	4.581404000	1.967746000
1	1.008593000	-3.764513000	1.577166000
1	2.862736000	-2.489894000	3.080204000
1	-3.730898000	-2.382572000	-0.525107000
1	-5.349460000	-0.218526000	-0.319955000
1	3.387889000	2.435631000	5.038784000
1	1.871689000	4.687233000	5.017595000
1	-1.258373000	-6.613213000	-1.919231000
1	-1.911198000	-4.508998000	1.787362000
1	-0.621385000	-4.475465000	-3.008472000
1	-1.917456000	-6.650266000	0.496441000
6	0.162124000	1.435417000	0.199794000
8	1.451554000	2.000046000	0.261117000
8	-0.086912000	0.924143000	-0.890537000
6	0.849759000	0.094353000	-4.391051000
6	1.354670000	1.402521000	-4.241335000
6	0.914535000	-0.517379000	-5.657509000
6	1.910103000	2.077838000	-5.325192000
6	1.477440000	0.173238000	-6.734812000
6	1.978029000	1.469456000	-6.584651000
1	1.336085000	1.864794000	-3.253204000
1	0.534571000	-1.527747000	-5.777983000
1	2.301490000	3.088407000	-5.181660000
1	1.522728000	-0.318183000	-7.710557000
1	2.416345000	1.998239000	-7.434024000
6	3.267719000	-0.116692000	-1.745749000
6	2.657414000	-1.018716000	-0.856805000
6	2.608127000	-2.378217000	-1.171603000
6	3.164121000	-2.862097000	-2.358891000
6	3.789808000	-1.964098000	-3.232037000
6	3.845079000	-0.603863000	-2.930852000
1	2.210890000	-0.651354000	0.067802000
1	2.116470000	-3.061961000	-0.478388000
1	3.106300000	-3.924944000	-2.603298000
1	4.228210000	-2.324183000	-4.166629000
1	4.306601000	0.108690000	-3.617127000
8	3.319311000	1.213258000	-1.516065000
1	1.574061000	2.331483000	1.166805000
1	2.685837000	1.469727000	-0.804050000

^{5}TS2$_{\text{III,phenol}}$:

26	-1.358122000	-0.213444000	20.372975000
8	-0.844839000	-4.091744000	14.270202000
7	-0.352632000	-2.044932000	20.670732000
7	-0.109402000	0.647373000	21.805382000
7	-1.083801000	-3.551922000	16.496259000
7	-0.247902000	-2.004364000	15.035462000
7	-2.692842000	1.390039000	20.635092000
7	-2.933206000	-1.309879000	19.527005000
6	-2.408529000	2.566708000	21.273556000
6	-0.632830000	-3.229063000	20.041814000
6	-4.115180000	-0.777998000	19.078919000
6	1.049360000	0.108325000	22.298229000
6	-2.823577000	-2.574362000	19.007563000
6	-3.520622000	3.488878000	21.107019000
1	2.434530000	-1.487689000	22.538901000
6	-3.936076000	1.513697000	20.079594000
6	-4.468409000	2.835245000	20.369507000
6	-4.585906000	0.515614000	19.345056000
6	0.415125000	-4.193115000	20.346977000
6	0.829545000	-2.202701000	21.343819000
1	-5.563465000	0.770192000	18.930201000
1	-1.151256000	3.825853000	22.437386000
6	1.316508000	-3.557526000	21.153385000
6	-1.751057000	-3.471623000	19.216772000
6	1.485503000	-1.206450000	22.078020000
6	-3.998128000	-2.863604000	18.204948000
6	-0.183150000	1.943059000	22.247329000
6	-1.449354000	-4.770706000	17.076814000
6	-1.786412000	-4.761331000	18.456581000
6	-1.238604000	2.830899000	21.995856000
6	-4.800219000	-1.755324000	18.258335000
6	1.747686000	1.104954000	23.084978000
6	0.984314000	2.242065000	23.052766000
6	-1.836170000	-7.167592000	17.020768000
6	-0.726523000	-3.287065000	15.187214000
6	-2.137996000	-5.961525000	19.082492000
6	-1.480918000	-5.985277000	16.368602000
6	-2.166198000	-7.167948000	18.378031000
1	-0.967649000	-2.794889000	17.161090000
1	-0.064985000	-1.472485000	15.880757000
1	-3.562285000	4.497746000	21.515484000
1	-5.443867000	3.200378000	20.050699000
1	0.441345000	-5.221469000	19.996567000
1	2.231540000	-3.960128000	21.585242000
1	-4.184180000	-3.799375000	17.683233000
1	-5.768839000	-1.604831000	17.783535000
1	2.695309000	0.945077000	23.597558000
1	1.181788000	3.198391000	23.535199000
1	-1.852887000	-8.101136000	16.452718000
1	-2.395717000	-5.937759000	20.143760000
1	-1.225496000	-5.982089000	15.312764000
1	-2.445363000	-8.094545000	18.883894000
6	-0.503224000	0.110403000	18.486183000
8	1.399963000	0.481805000	18.903416000
8	-0.569500000	-0.006090000	17.335204000
6	0.169310000	-1.371298000	13.858080000
6	0.905579000	-0.177946000	13.998480000
6	-0.122709000	-1.849897000	12.568209000
6	1.327039000	0.524989000	12.871578000
6	0.313767000	-1.134925000	11.448668000
6	1.035275000	0.053891000	11.585721000
1	1.175511000	0.169897000	14.998431000
1	-0.678581000	-2.776759000	12.455361000
1	1.900409000	1.446589000	13.001200000
1	0.079368000	-1.519036000	10.452316000
1	1.370160000	0.604722000	10.703915000
6	2.628522000	-1.535714000	16.711168000
6	2.023357000	-2.491214000	17.574616000
6	1.977840000	-3.843638000	17.232480000
6	2.520698000	-4.308153000	16.030455000
6	3.136990000	-3.383389000	15.174641000
6	3.198568000	-2.032758000	15.505899000
1	1.579146000	-2.149397000	18.510406000
1	1.494714000	-4.544223000	17.916271000
1	2.464184000	-5.366228000	15.764844000
1	3.570999000	-3.722671000	14.228801000
1	3.667206000	-1.312848000	14.830822000
8	2.657586000	-0.250074000	16.973312000
1	1.633827000	-0.017761000	19.699929000
1	2.025884000	0.093552000	18.005215000

^{5}IM2$_{\text{III,phenol}}$:

26	-0.683152000	1.174327000	1.901944000
8	-0.812363000	-2.510084000	-3.950115000
7	0.266025000	-0.675332000	2.241710000
7	0.515471000	2.011353000	3.385530000
7	-0.150389000	-2.374271000	-1.756312000
7	0.630575000	-0.843398000	-3.260751000
7	-2.010011000	2.800355000	2.142165000
7	-2.279109000	0.090490000	1.061612000
6	-1.721853000	3.975784000	2.779774000
6	-0.048439000	-1.875756000	1.660455000

6	-3.432771000	0.648238000	0.567246000
6	1.654778000	1.454562000	3.905234000
6	-2.195513000	-1.188721000	0.570277000
6	-2.807354000	4.920410000	2.570384000
1	2.999495000	-0.165595000	4.200604000
6	-3.230680000	2.947451000	1.543489000
6	-3.743678000	4.282145000	1.805474000
6	-3.878132000	1.957097000	0.798122000
6	0.948414000	-2.867755000	2.033731000
6	1.416057000	-0.856613000	2.962813000
1	-4.834878000	2.228982000	0.347439000
1	-0.471157000	5.216645000	3.971316000
6	1.852920000	-2.235751000	2.840133000
6	-1.162972000	-2.115209000	0.834823000
6	2.073335000	0.132227000	3.705044000
6	-3.353545000	-1.457524000	-0.260343000
6	0.458819000	3.311365000	3.817786000
6	-0.786897000	-3.501917000	-1.220170000
6	-1.248198000	-3.425974000	0.115744000
6	-0.568573000	4.220721000	3.534321000
6	-4.121061000	-0.323161000	-0.254993000
6	2.354195000	2.442407000	4.701869000
6	1.613283000	3.592833000	4.647356000
6	-1.547276000	-5.800722000	-1.343924000
6	-0.160534000	-1.955451000	-3.072774000
6	-1.826773000	-4.555618000	0.704393000
6	-0.949027000	-4.692857000	-1.946087000
6	-1.979164000	-5.743748000	-0.015197000
1	0.293972000	-1.762244000	-1.079872000
1	1.234780000	-0.575513000	-2.484987000
1	-2.840263000	5.932863000	2.970645000
1	-4.700084000	4.665062000	1.451869000
1	0.937564000	-3.910434000	1.725010000
1	2.736742000	-2.656264000	3.317403000
1	-3.552601000	-2.396554000	-0.770927000
1	-5.069352000	-0.152251000	-0.762664000
1	3.287210000	2.267157000	5.235699000
1	1.817765000	4.548275000	5.128532000
1	-1.666773000	-6.721961000	-1.919341000
1	-2.178318000	-4.486869000	1.736373000
1	-0.606008000	-4.737561000	-2.977903000
1	-2.440097000	-6.614828000	0.455311000
6	-0.064897000	1.573667000	-0.068812000
8	2.179214000	2.218503000	0.773874000
8	0.032032000	1.664345000	-1.197650000
6	0.735138000	-0.029555000	-4.397321000
6	1.480305000	1.161508000	-4.270334000
6	0.140333000	-0.339823000	-5.634138000
6	1.623922000	2.015928000	-5.362395000
6	0.291837000	0.533545000	-6.715574000
6	1.031285000	1.712712000	-6.594158000
1	1.945125000	1.412736000	-3.313105000
1	-0.430729000	-1.258988000	-5.732591000
1	2.206371000	2.933758000	-5.245910000
1	-0.177202000	0.278888000	-7.669765000
1	1.145530000	2.386835000	-7.446082000
6	3.381609000	0.130303000	-1.547303000
6	2.817423000	-0.808931000	-0.619586000
6	2.929567000	-2.188537000	-0.816894000
6	3.567422000	-2.715438000	-1.944602000
6	4.119266000	-1.820204000	-2.875111000
6	4.036533000	-0.444554000	-2.686584000
1	2.307499000	-0.420233000	0.264746000
1	2.496348000	-2.861561000	-0.073985000
1	3.633774000	-3.795101000	-2.096426000
1	4.622222000	-2.207918000	-3.767220000
1	4.467891000	0.244976000	-3.417360000
8	3.289367000	1.410662000	-1.402819000
1	2.484439000	1.620365000	1.469747000
1	2.639265000	1.890775000	-0.075595000

[Fe^{II}(*ortho*-urea-TPP)(HCO_3)]$^{3-}$
Solvent = n,n-dimethylformamide:

^{3}RC1$_{III, solvent = DMF}$:

26	-0.897067000	-0.856550000	19.796257000
8	-0.784669000	-6.655526000	15.574621000
7	-0.402203000	-2.767330000	19.392478000
7	0.943494000	-0.518559000	20.621191000
7	-2.082236000	-4.795271000	15.942203000
7	-0.261591000	-4.647348000	14.557741000
7	-1.479152000	0.938654000	20.532533000
7	-2.826672000	-1.344303000	19.399720000
6	-0.695732000	1.910210000	21.132753000
6	-1.195543000	-3.778069000	18.867163000
6	-3.892978000	-0.467076000	19.425034000
6	2.028849000	-1.361360000	20.528402000
6	-3.322324000	-2.527200000	18.873692000
6	-1.486382000	3.103550000	21.387456000
1	2.931983000	-3.219940000	19.966748000
6	-2.737320000	1.492085000	20.406805000
6	-2.745258000	2.846037000	20.937357000
6	-3.848284000	0.863347000	19.889015000
6	-0.383763000	-4.960767000	18.613799000
6	0.877155000	-3.286823000	19.469459000
1	-4.780960000	1.431304000	19.855149000
1	1.130753000	2.641887000	21.923877000
6	0.890886000	-4.652130000	18.972978000
6	-2.558155000	-3.701303000	18.623415000
6	1.994932000	-2.658218000	19.975313000
6	-4.710396000	-2.389420000	18.573224000
6	1.418417000	0.639485000	21.208409000
6	-2.991798000	-5.435100000	16.802404000
6	-3.266549000	-4.900925000	18.085764000
6	0.638247000	1.787806000	21.453602000
6	-5.068537000	-1.094463000	18.923273000
6	3.193910000	-0.743737000	21.074630000
6	2.807147000	0.518614000	21.507201000
6	-4.576812000	-7.244799000	17.203133000
6	-1.025247000	-5.462250000	15.369894000
6	-4.224760000	-5.552024000	18.884328000
6	-3.648955000	-6.606680000	16.383139000
6	-4.876260000	-6.710608000	18.461894000
1	-2.062747000	-3.745069000	15.932125000
1	-0.634177000	-3.701381000	14.312270000
1	-1.103507000	4.008644000	21.859023000
1	-3.621045000	3.494813000	20.960897000
1	-0.750550000	-5.893473000	18.193683000
1	1.778327000	-5.283368000	18.924306000
1	-5.339819000	-3.165016000	18.144293000
1	-6.045987000	-0.618431000	18.843318000
1	4.179810000	-1.206052000	21.127437000
1	3.414964000	1.291022000	21.978561000
1	-5.074926000	-8.153470000	16.855155000
1	-4.440441000	-5.135964000	19.871034000
1	-3.419681000	-7.007490000	15.395415000
1	-5.605175000	-7.199340000	19.113047000
6	-0.615803000	-0.040082000	18.011795000
8	-1.499800000	0.629629000	17.438432000
8	0.520635000	-0.363500000	17.655706000
1	-1.408313000	0.130756000	15.851398000
8	-1.209201000	-0.175141000	14.918492000
8	-0.963786000	-2.084449000	13.810667000

6	-1.407252000	-1.515535000	14.844851000
8	-2.007077000	-2.078735000	15.794389000
6	0.993260000	-4.926752000	14.017349000
6	1.619445000	-3.892362000	13.282502000
6	1.677411000	-6.151777000	14.168454000
6	2.880045000	-4.081556000	12.720227000
6	2.943631000	-6.320830000	13.600694000
6	3.558087000	-5.297605000	12.873212000
1	1.089582000	-2.943358000	13.176736000
1	1.201083000	-6.952486000	14.727070000
1	3.340965000	-3.264631000	12.157855000
1	3.456080000	-7.278257000	13.731759000
1	4.547253000	-5.443064000	12.432812000

^{3}IM1$_{III,\ solvent\ =\ DMF}$:

26	-0.880576000	-0.875374000	19.756857000
8	-0.749486000	-6.563826000	15.664010000
7	-0.405008000	-2.749281000	19.362789000
7	1.004200000	-0.570908000	20.624131000
7	-2.116805000	-4.732291000	15.908964000
7	-0.253823000	-4.571351000	14.594206000
7	-1.415439000	0.905121000	20.409306000
7	-2.886716000	-1.410326000	19.517441000
6	-0.597230000	1.902013000	20.926309000
6	-1.213677000	-3.749847000	18.827715000
6	-3.945438000	-0.538958000	19.593305000
6	2.059955000	-1.445665000	20.592046000
6	-3.377798000	-2.570143000	18.969887000
6	-1.369964000	3.110349000	21.153285000
1	2.894711000	-3.341523000	20.060351000
6	-2.691025000	1.453801000	20.359236000
6	-2.656379000	2.833203000	20.807668000
6	-3.851557000	0.802493000	19.997796000
6	-0.415186000	-4.938331000	18.571117000
6	0.862935000	-3.308318000	19.480462000
1	-4.776201000	1.383422000	20.016718000
1	1.237001000	2.665243000	21.663069000
6	0.854372000	-4.664531000	18.969145000
6	-2.589093000	-3.696584000	18.622990000
6	1.985738000	-2.736382000	20.045182000
6	-4.787698000	-2.440021000	18.725891000
6	1.496101000	0.606298000	21.128998000
6	-3.017647000	-5.390501000	16.760727000
6	-3.286185000	-4.892772000	18.060986000
6	0.738633000	1.783146000	21.255987000
6	-5.142676000	-1.167002000	19.125631000
6	3.237586000	-0.828258000	21.125205000
6	2.883579000	0.465549000	21.461298000
6	-4.587928000	-7.223432000	17.140645000
6	-1.022313000	-5.383584000	15.400453000
6	-4.225663000	-5.571754000	18.859018000
6	-3.674707000	-6.557618000	16.326311000
6	-4.873907000	-6.724573000	18.416672000
1	-2.202068000	-3.655424000	15.775005000
1	-0.654109000	-3.616716000	14.296571000
1	-0.957063000	4.036611000	21.551609000
1	-3.529136000	3.483437000	20.860123000
1	-0.790545000	-5.857298000	18.130957000
1	1.726622000	-5.316992000	18.943072000
1	-5.424365000	-3.204197000	18.287882000
1	-6.123713000	-0.694603000	19.081117000
1	4.211401000	-1.308737000	21.217285000
1	3.509976000	1.250512000	21.884355000
1	-5.084122000	-8.126940000	16.776590000
1	-4.429567000	-5.185287000	19.860146000
1	-3.453623000	-6.932430000	15.326104000
1	-5.589883000	-7.234896000	19.065491000
6	-0.506628000	-0.262136000	17.767043000
8	-1.546847000	0.341907000	17.177588000
8	0.501166000	-0.548910000	17.127761000
1	-1.532348000	0.177811000	16.191250000
8	-1.599404000	-0.232609000	14.700003000
8	-1.025111000	-2.186048000	13.773731000
6	-1.667692000	-1.518114000	14.679039000
8	-2.373568000	-2.148967000	15.556949000
6	1.030128000	-4.826400000	14.129002000
6	1.665517000	-3.785535000	13.406082000
6	1.747415000	-6.026426000	14.337429000
6	2.958492000	-3.944680000	12.913445000
6	3.046112000	-6.164745000	13.838941000
6	3.666424000	-5.135863000	13.123879000
1	1.106510000	-2.858094000	13.260349000
1	1.267230000	-6.831007000	14.887485000
1	3.423380000	-3.123359000	12.360157000
1	3.580853000	-7.102987000	14.014944000
1	4.681343000	-5.257474000	12.737837000

^{3}RC2$_{III,\ solvent\ =\ DMF}$:

26	0.152994000	1.395786000	1.520871000
8	0.128008000	-4.271117000	-2.393642000
7	0.591763000	-0.522521000	1.157196000
7	1.973992000	1.637721000	2.484766000
7	-1.455607000	-2.631514000	-2.118776000
7	0.536394000	-2.099365000	-3.089326000
7	-0.384244000	3.205101000	2.193877000
7	-1.817459000	0.938038000	1.162265000
6	0.415281000	4.151005000	2.800629000
6	-0.240467000	-1.531681000	0.711560000
6	-2.853405000	1.838651000	1.172538000
6	3.028255000	0.769561000	2.411137000
6	-2.347615000	-0.252316000	0.725542000
6	-0.324265000	5.384728000	2.996867000
1	3.884258000	-1.100260000	1.854731000
6	-1.612970000	3.801171000	2.030396000
6	-1.580988000	5.168602000	2.515484000
6	-2.756528000	3.179269000	1.545410000
6	0.519933000	-2.756814000	0.526204000
6	1.842643000	-1.092927000	1.286692000
1	-3.667162000	3.779192000	1.490558000
1	2.237154000	4.816956000	3.667166000
6	1.809016000	-2.477894000	0.862767000
6	-1.624478000	-1.437698000	0.512286000
6	2.971261000	-0.501421000	1.836879000
6	-3.763153000	-0.101995000	0.475407000
6	2.461387000	2.787505000	3.047112000
6	-2.221690000	-3.286962000	-1.132985000
6	-2.371830000	-2.680796000	0.135708000
6	1.731593000	3.968426000	3.201887000
6	-4.075441000	1.201509000	0.749845000
6	4.210918000	1.364997000	2.986410000
6	3.855608000	2.627463000	3.388600000
6	-3.637019000	-5.135759000	-0.432156000
6	-0.231429000	-3.100123000	-2.533010000
6	-3.196987000	-3.306392000	1.084669000
6	-2.844778000	-4.514674000	-1.398619000
6	-3.827330000	-4.521872000	0.810108000
1	-1.737505000	-1.648664000	-2.338257000
1	0.190203000	-1.121476000	-2.936404000
1	0.083094000	6.283165000	3.458972000
1	-2.429683000	5.851439000	2.498166000
1	0.113411000	-3.698331000	0.170553000
1	2.669151000	-3.146242000	0.859369000
1	-4.418299000	-0.889565000	0.112304000
1	-5.041427000	1.698341000	0.667031000

1	5.183141000	0.878740000	3.060532000
1	4.475849000	3.389715000	3.858875000
1	-4.117331000	-6.092077000	-0.653489000
1	-3.320936000	-2.835899000	2.062799000
1	-2.703552000	-4.967254000	-2.380918000
1	-4.455583000	-4.994304000	1.568972000
6	1.016347000	2.232162000	-0.267744000
8	0.253993000	3.174220000	-1.012342000
8	2.165120000	2.114098000	-0.665891000
1	0.852291000	3.478554000	-1.727355000
8	-1.904112000	1.897478000	-2.070828000
8	-0.263031000	0.458354000	-2.579976000
6	-1.503982000	0.668664000	-2.491585000
8	-2.415643000	-0.149392000	-2.757223000
6	1.857428000	-2.190512000	-3.518908000
6	2.565062000	-0.977158000	-3.680045000
6	2.519811000	-3.401562000	-3.808156000
6	3.886869000	-0.978628000	-4.121304000
6	3.846642000	-3.384191000	-4.246775000
6	4.542330000	-2.182223000	-4.409937000
1	2.057249000	-0.041293000	-3.436930000
1	1.986822000	-4.339072000	-3.671658000
1	4.413253000	-0.026994000	-4.235146000
1	4.343315000	-4.333813000	-4.465289000
1	5.579427000	-2.181918000	-4.753212000
1	-1.110492000	2.374626000	-1.728637000

^{3}TS2$_{\text{III, solvent = DMF}}$:

26	-0.855558000	-1.015034000	19.550850000
8	-0.794209000	-6.607702000	15.509605000
7	-0.410224000	-2.962751000	19.248560000
7	0.979674000	-0.710594000	20.391271000
7	-2.301676000	-4.920665000	15.916235000
7	-0.329181000	-4.416793000	14.906395000
7	-1.379802000	0.843679000	20.136538000
7	-2.776778000	-1.444518000	19.107435000
6	-0.570807000	1.813493000	20.684365000
6	-1.235649000	-3.965436000	18.789810000
6	-3.813007000	-0.541136000	19.085973000
6	2.028479000	-1.595428000	20.375914000
6	-3.311253000	-2.645651000	18.692836000
6	-1.301071000	3.054223000	20.828766000
1	2.889782000	-3.487071000	19.929073000
6	-2.600213000	1.438003000	19.936556000
6	-2.563010000	2.820913000	20.360180000
6	-3.734207000	0.800835000	19.442403000
6	-0.480660000	-5.191002000	18.622689000
6	0.838799000	-3.525506000	19.382357000
1	-4.645791000	1.394915000	19.355957000
1	1.276556000	2.500430000	21.486664000
6	0.807038000	-4.912503000	18.979046000
6	-2.610844000	-3.845446000	18.535108000
6	1.972727000	-2.896481000	19.886098000
6	-4.722788000	-2.490395000	18.408668000
6	1.474180000	0.456242000	20.921947000
6	-3.115855000	-5.626706000	16.816109000
6	-3.348202000	-5.071190000	18.096076000
6	0.756902000	1.641087000	21.059275000
6	-5.033206000	-1.185301000	18.654094000
6	3.212059000	-0.981774000	20.936306000
6	2.866538000	0.295772000	21.279664000
6	-4.558833000	-7.503941000	17.372392000
6	-1.114900000	-5.416514000	15.439602000
6	-4.221614000	-5.728085000	18.975772000
6	-3.713781000	-6.849931000	16.474579000
6	-4.828509000	-6.935523000	18.621985000
1	-2.557401000	-3.898947000	15.738719000
1	-0.631932000	-3.420046000	15.110918000
1	-0.887059000	3.973049000	21.241684000
1	-3.406006000	3.508667000	20.307121000
1	-0.884593000	-6.130772000	18.258549000
1	1.666698000	-5.581211000	18.985531000
1	-5.374094000	-3.284381000	18.053898000
1	-5.994836000	-0.684733000	18.549163000
1	4.176046000	-1.477106000	21.045558000
1	3.485924000	1.070559000	21.729338000
1	-5.019353000	-8.454284000	17.090688000
1	-4.402959000	-5.292205000	19.961141000
1	-3.509240000	-7.273006000	15.490684000
1	-5.498445000	-7.437023000	19.324349000
6	0.192276000	-0.393516000	17.679167000
8	-0.871003000	1.085411000	16.672779000
8	1.250398000	-0.380918000	17.214961000
1	-0.217347000	1.226855000	15.972190000
8	-2.576078000	-0.322258000	15.681720000
8	-0.960427000	-1.861179000	15.463110000
6	-2.206310000	-1.571823000	15.501853000
8	-3.115049000	-2.453387000	15.366166000
6	0.932078000	-4.540846000	14.337139000
6	1.614321000	-3.339409000	14.029240000
6	1.566236000	-5.767209000	14.041745000
6	2.880655000	-3.367597000	13.449456000
6	2.836643000	-5.775558000	13.459027000
6	3.507169000	-4.586225000	13.157111000
1	1.121853000	-2.393502000	14.264940000
1	1.053362000	-6.693869000	14.285692000
1	3.386178000	-2.424336000	13.224127000
1	3.309910000	-6.737004000	13.239236000
1	4.500805000	-4.606660000	12.703443000
1	-1.720785000	0.343558000	16.130399000

^{3}IM2$_{\text{III, solvent = DMF}}$:

26	0.055263000	1.229762000	1.742028000
8	0.216376000	-4.105203000	-2.389483000
7	0.462419000	-0.732317000	1.541075000
7	1.926806000	1.569888000	2.450947000
7	-1.299447000	-2.458015000	-1.855957000
7	0.654465000	-1.873944000	-2.852181000
7	-0.390135000	3.163071000	2.094387000
7	-1.867547000	0.834719000	1.309900000
6	0.470740000	4.166421000	2.482676000
6	-0.373723000	-1.722422000	1.076321000
6	-2.892622000	1.750944000	1.277924000
6	2.941187000	0.647350000	2.545197000
6	-2.420364000	-0.365563000	0.915910000
6	-0.223407000	5.433750000	2.529784000
1	3.732805000	-1.311639000	2.313160000
6	-1.610765000	3.765230000	1.908368000
6	-1.518786000	5.183356000	2.175104000
6	-2.782167000	3.110855000	1.543470000
6	0.349119000	-2.973547000	0.974792000
6	1.689241000	-1.326297000	1.736536000
1	-3.687459000	3.713631000	1.453298000
1	2.371130000	4.881616000	3.118146000
6	1.627191000	-2.725592000	1.384922000
6	-1.735138000	-1.574255000	0.771917000
6	2.837069000	-0.697261000	2.206124000
6	-3.832196000	-0.198181000	0.644646000
6	2.477643000	2.776354000	2.816296000
6	-2.139963000	-3.242354000	-1.055833000
6	-2.451752000	-2.775030000	0.243833000
6	1.808195000	3.995889000	2.819047000
6	-4.127233000	1.111497000	0.882434000
6	4.154793000	1.280544000	3.008537000

6	3.867383000	2.606713000	3.175152000
6	-3.576674000	-5.171106000	-0.690801000
6	-0.117937000	-2.914296000	-2.375615000
6	-3.356451000	-3.494983000	1.035788000
6	-2.697073000	-4.452065000	-1.502063000
6	-3.923143000	-4.686651000	0.575369000
1	-1.576670000	-1.412062000	-1.983549000
1	0.336651000	-0.887921000	-2.602796000
1	0.234887000	6.381201000	2.809490000
1	-2.350137000	5.882967000	2.100213000
1	-0.072016000	-3.909756000	0.620915000
1	2.464270000	-3.419241000	1.449799000
1	-4.495000000	-0.990156000	0.306945000
1	-5.084523000	1.621222000	0.782903000
1	5.099479000	0.764194000	3.173563000
1	4.524905000	3.408869000	3.507043000
1	-4.004429000	-6.109192000	-1.053997000
1	-3.593832000	-3.125338000	2.036346000
1	-2.432662000	-4.813356000	-2.496124000
1	-4.619815000	-5.241697000	1.207937000
6	1.208139000	1.484299000	-0.358525000
8	0.221571000	3.857347000	-1.671520000
8	2.263422000	1.501489000	-0.793916000
1	0.966380000	3.422243000	-2.105620000
8	-1.673314000	2.147047000	-2.148940000
8	-0.006729000	0.648162000	-2.215998000
6	-1.282581000	0.932169000	-2.212412000
8	-2.134573000	-0.030918000	-2.286432000
6	1.887629000	-1.950092000	-3.485271000
6	2.507661000	-0.722626000	-3.823932000
6	2.554401000	-3.150596000	-3.815970000
6	3.744294000	-0.701228000	-4.464329000
6	3.793802000	-3.108751000	-4.461046000
6	4.403043000	-1.894208000	-4.790982000
1	1.986649000	0.201640000	-3.565126000
1	2.087674000	-4.095961000	-3.551828000
1	4.200662000	0.261325000	-4.712505000
1	4.292244000	-4.050966000	-4.707355000
1	5.373229000	-1.875447000	-5.293001000
1	-0.518484000	3.182422000	-1.844476000

^{5}RC1$_{\text{III, solvent = DMF}}$:

26	0.007428000	1.573158000	1.832540000
8	0.166679000	-4.386935000	-2.347147000
7	0.546885000	-0.411802000	1.465753000
7	1.855944000	1.874577000	2.815626000
7	-1.087041000	-2.499026000	-1.964112000
7	0.695931000	-2.401897000	-3.402762000
7	-0.670124000	3.343741000	2.707055000
7	-2.001308000	0.984882000	1.526177000
6	0.108123000	4.304791000	3.316181000
6	-0.242625000	-1.413760000	0.936556000
6	-3.073017000	1.848850000	1.563413000
6	2.943617000	1.042175000	2.727667000
6	-2.436363000	-0.187873000	0.947165000
6	-0.715659000	5.476569000	3.598917000
1	3.856255000	-0.815903000	2.176854000
6	-1.946963000	3.847127000	2.601879000
6	-1.976331000	5.195656000	3.159351000
6	-3.040213000	3.175701000	2.075057000
6	0.587186000	-2.597786000	0.711902000
6	1.824778000	-0.913435000	1.601863000
1	-3.987373000	3.721477000	2.053871000
1	1.930150000	5.040079000	4.108587000
6	1.851954000	-2.285576000	1.110544000
6	-1.616257000	-1.340075000	0.693153000
6	2.914476000	-0.260707000	2.159505000
6	-3.823331000	-0.064660000	0.614091000
6	2.282717000	3.048613000	3.393820000
6	-2.008560000	-3.118561000	-1.102737000
6	-2.305613000	-2.554884000	0.163355000
6	1.453891000	4.180280000	3.630095000
6	-4.221392000	1.211178000	1.004537000
6	4.091619000	1.690116000	3.283026000
6	3.676222000	2.952254000	3.701570000
6	-3.602599000	-4.919251000	-0.690027000
6	-0.057792000	-3.191832000	-2.558240000
6	-3.277960000	-3.190927000	0.958584000
6	-2.661052000	-4.298812000	-1.507434000
6	-3.922300000	-4.358583000	0.552248000
1	-1.034252000	-1.451918000	-1.968663000
1	0.336605000	-1.451386000	-3.644767000
1	-0.358771000	6.386847000	4.081513000
1	-2.861931000	5.829945000	3.209573000
1	0.240593000	-3.539208000	0.292887000
1	2.735610000	-2.924247000	1.093716000
1	-4.431494000	-0.831568000	0.140016000
1	-5.209688000	1.661881000	0.907661000
1	5.090065000	1.256676000	3.350553000
1	4.273708000	3.733320000	4.172728000
1	-4.095246000	-5.834889000	-1.027423000
1	-3.510397000	-2.753201000	1.931909000
1	-2.416607000	-4.721569000	-2.481964000
1	-4.661102000	-4.833337000	1.202667000
6	0.535224000	2.405533000	0.015532000
8	-0.426416000	2.970748000	-0.536219000
8	1.702570000	2.200445000	-0.294552000
1	-0.372853000	2.432113000	-2.167837000
8	-0.171471000	2.109150000	-3.085281000
8	0.019610000	0.174586000	-4.160660000
6	-0.361528000	0.761192000	-3.114739000
8	-0.890258000	0.223500000	-2.110785000
6	1.909684000	-2.727295000	-4.008487000
6	2.524641000	-1.722266000	-4.791417000
6	2.560947000	-3.972811000	-3.882151000
6	3.742518000	-1.959555000	-5.425159000
6	3.784598000	-4.190386000	-4.522058000
6	4.387734000	-3.196164000	-5.297588000
1	2.020081000	-0.757914000	-4.879843000
1	2.091942000	-4.751380000	-3.287242000
1	4.195296000	-1.164868000	-6.024686000
1	4.271917000	-5.163193000	-4.409863000
1	5.343199000	-3.379745000	-5.794501000

^{5}TS1$_{\text{III, solvent = DMF}}$:

26	-0.795359000	-0.659718000	19.505073000
8	-0.698720000	-6.636847000	15.686053000
7	-0.379529000	-2.778231000	19.420634000
7	0.982816000	-0.477075000	20.677687000
7	-2.036870000	-4.786639000	15.954696000
7	-0.257908000	-4.685710000	14.523261000
7	-1.535934000	1.009775000	20.625655000
7	-2.864970000	-1.330455000	19.438746000
6	-0.751170000	1.947027000	21.238599000
6	-1.167263000	-3.762894000	18.890937000
6	-3.921878000	-0.461881000	19.493868000
6	2.036389000	-1.344099000	20.651445000
6	-3.318299000	-2.487170000	18.866099000
6	-1.557156000	3.109573000	21.580550000
1	2.916063000	-3.219059000	20.163256000
6	-2.803863000	1.511952000	20.570282000
6	-2.833933000	2.837829000	21.166460000
6	-3.902993000	0.838005000	20.023081000
6	-0.370256000	-4.966267000	18.684338000

6	0.879586000	-3.296678000	19.567357000
1	-4.853586000	1.377013000	20.014557000
1	1.100086000	2.643183000	22.024508000
6	0.899030000	-4.672166000	19.096941000
6	-2.548174000	-3.646286000	18.613220000
6	1.989043000	-2.641775000	20.120377000
6	-4.730969000	-2.345577000	18.537808000
6	1.410927000	0.670768000	21.282293000
6	-2.949587000	-5.413344000	16.814777000
6	-3.254196000	-4.851329000	18.078809000
6	0.613618000	1.800727000	21.526660000
6	-5.103551000	-1.089704000	18.930948000
6	3.196983000	-0.723210000	21.265591000
6	2.808411000	0.532136000	21.657051000
6	-4.533747000	-7.221518000	17.239009000
6	-0.976696000	-5.465490000	15.403050000
6	-4.221793000	-5.484403000	18.880513000
6	-3.594875000	-6.600416000	16.418751000
6	-4.860511000	-6.656647000	18.476987000
1	-2.072194000	-3.723396000	15.846179000
1	-0.686446000	-3.767411000	14.205334000
1	-1.187856000	4.006073000	22.078326000
1	-3.719206000	3.466918000	21.258886000
1	-0.728950000	-5.900699000	18.260631000
1	1.772973000	-5.323405000	19.094013000
1	-5.348720000	-3.106097000	18.066704000
1	-6.085326000	-0.623626000	18.849071000
1	4.174716000	-1.190714000	21.381514000
1	3.404777000	1.295203000	22.156997000
1	-5.020568000	-8.141955000	16.906333000
1	-4.452792000	-5.047745000	19.854752000
1	-3.347113000	-7.026141000	15.445971000
1	-5.599228000	-7.131140000	19.127243000
6	-0.291488000	-0.001562000	17.536200000
8	-1.272778000	0.563394000	16.853005000
8	0.766451000	-0.338241000	16.958137000
1	-1.302595000	0.135438000	15.683382000
8	-1.348559000	-0.314534000	14.587753000
8	-1.126904000	-2.313146000	13.631815000
6	-1.535964000	-1.616196000	14.625057000
8	-2.110751000	-2.143148000	15.633587000
6	0.990663000	-4.961636000	13.974412000
6	1.552576000	-3.968400000	13.136028000
6	1.733627000	-6.139398000	14.208458000
6	2.805790000	-4.151291000	12.556149000
6	2.991631000	-6.302512000	13.620886000
6	3.541599000	-5.320149000	12.792031000
1	0.973081000	-3.057597000	12.968196000
1	1.306778000	-6.909119000	14.845627000
1	3.215976000	-3.367038000	11.913426000
1	3.549982000	-7.222473000	13.817573000
1	4.525103000	-5.460770000	12.337426000

^{5}IM1$_{III, solvent = DMF}$:

26	0.109297000	1.667011000	1.619749000
8	0.226480000	-4.313795000	-2.205978000
7	0.544227000	-0.434225000	1.471786000
7	1.917071000	1.860888000	2.728770000
7	-1.104274000	-2.450246000	-1.982962000
7	0.705593000	-2.370630000	-3.371099000
7	-0.610344000	3.334804000	2.742603000
7	-1.946064000	1.006401000	1.538490000
6	0.179382000	4.265795000	3.358877000
6	-0.247664000	-1.414668000	0.938737000
6	-3.003406000	1.872835000	1.614427000
6	2.974116000	0.999005000	2.674119000
6	-2.399464000	-0.139906000	0.943617000
6	-0.626650000	5.418793000	3.729541000
1	3.852828000	-0.868089000	2.157161000
6	-1.880989000	3.833011000	2.710550000
6	-1.907807000	5.148892000	3.327812000
6	-2.983564000	3.163489000	2.165470000
6	0.549072000	-2.614555000	0.714836000
6	1.806201000	-0.950253000	1.597334000
1	-3.935732000	3.699544000	2.174490000
1	2.040596000	4.960719000	4.122547000
6	1.822247000	-2.321407000	1.115324000
6	-1.630173000	-1.295183000	0.670215000
6	2.922967000	-0.294420000	2.134554000
6	-3.811893000	0.009229000	0.619344000
6	2.348824000	3.002582000	3.343651000
6	-2.027520000	-3.065577000	-1.129293000
6	-2.340554000	-2.495508000	0.130579000
6	1.549984000	4.123323000	3.620457000
6	-4.185079000	1.255577000	1.040544000
6	4.141312000	1.618229000	3.277044000
6	3.752813000	2.865625000	3.692446000
6	-3.631038000	-4.860440000	-0.706162000
6	-0.039644000	-3.140659000	-2.505344000
6	-3.318930000	-3.117545000	0.928352000
6	-2.680267000	-4.251098000	-1.521322000
6	-3.963650000	-4.286956000	0.526428000
1	-1.166934000	-1.369120000	-2.143582000
1	0.292732000	-1.429650000	-3.698271000
1	-0.254118000	6.308875000	4.236231000
1	-2.793948000	5.773086000	3.441619000
1	0.189556000	-3.545343000	0.284368000
1	2.697644000	-2.970277000	1.095037000
1	-4.428913000	-0.740242000	0.130256000
1	-5.166779000	1.723229000	0.968340000
1	5.122795000	1.153904000	3.371287000
1	4.353158000	3.624322000	4.194146000
1	-4.121977000	-5.779205000	-1.037777000
1	-3.553483000	-2.675297000	1.899232000
1	-2.426454000	-4.685006000	-2.488986000
1	-4.710705000	-4.752460000	1.173682000
6	0.535130000	2.285019000	-0.378416000
8	-0.525962000	2.813027000	-1.035803000
8	1.534704000	1.956802000	-1.032001000
1	-0.519194000	2.525877000	-2.036054000
8	-0.652785000	1.970784000	-3.451858000
8	-0.118642000	-0.039183000	-4.271432000
6	-0.689576000	0.687686000	-3.364147000
8	-1.295022000	0.116924000	-2.375554000
6	1.952636000	-2.669587000	-3.904011000
6	2.544399000	-1.683088000	-4.732361000
6	2.673064000	-3.862729000	-3.669684000
6	3.799182000	-1.887402000	-5.300985000
6	3.933172000	-4.047121000	-4.246319000
6	4.510500000	-3.071763000	-5.065040000
1	1.982429000	-0.761206000	-4.900026000
1	2.225148000	-4.626520000	-3.039804000
1	4.230763000	-1.107914000	-5.935744000
1	4.471794000	-4.978882000	-4.049182000
1	5.495300000	-3.229250000	-5.511374000

^{5}RC2$_{III, solvent = DMF}$:

26	0.204271000	1.454631000	1.311649000
8	0.125634000	-4.270191000	-2.358609000
7	0.606694000	-0.617714000	1.160523000
7	2.005020000	1.639318000	2.425715000
7	-1.465399000	-2.633369000	-2.114707000
7	0.546308000	-2.097692000	-3.043921000
7	-0.431451000	3.254270000	2.249909000

7	-1.843790000	0.951490000	1.095610000
6	0.388644000	4.180776000	2.834276000
6	-0.248872000	-1.599933000	0.732837000
6	-2.884710000	1.839025000	1.178639000
6	3.041173000	0.750231000	2.386195000
6	-2.360106000	-0.251034000	0.684083000
6	-0.362520000	5.399681000	3.091908000
1	3.873035000	-1.159314000	1.950900000
6	-1.669653000	3.817554000	2.131989000
6	-1.639898000	5.173858000	2.655778000
6	-2.800045000	3.166678000	1.619891000
6	0.481562000	-2.855294000	0.623096000
6	1.834857000	-1.195346000	1.356353000
1	-3.729572000	3.740627000	1.606404000
1	2.263529000	4.820107000	3.607941000
6	1.771183000	-2.599707000	0.997545000
6	-1.635292000	-1.448186000	0.501846000
6	2.962895000	-0.558332000	1.886633000
6	-3.792847000	-0.113016000	0.483604000
6	2.481316000	2.803553000	2.963961000
6	-2.241937000	-3.290232000	-1.137282000
6	-2.397061000	-2.685620000	0.130667000
6	1.736703000	3.977648000	3.153761000
6	-4.115168000	1.180491000	0.788113000
6	4.230758000	1.365165000	2.945647000
6	3.883528000	2.641122000	3.304656000
6	-3.665040000	-5.135257000	-0.445956000
6	-0.231162000	-3.099336000	-2.503478000
6	-3.226113000	-3.309678000	1.076491000
6	-2.866893000	-4.515436000	-1.408609000
6	-3.858485000	-4.523133000	0.796785000
1	-1.738199000	-1.645479000	-2.321765000
1	0.196454000	-1.119738000	-2.902401000
1	0.041075000	6.297678000	3.558726000
1	-2.492132000	5.852096000	2.689397000
1	0.064002000	-3.800537000	0.289404000
1	2.605391000	-3.298716000	1.048498000
1	-4.456118000	-0.905710000	0.147190000
1	-5.096203000	1.653614000	0.757978000
1	5.200466000	0.878373000	3.046273000
1	4.510193000	3.406193000	3.761965000
1	-4.146994000	-6.090042000	-0.670308000
1	-3.351510000	-2.841043000	2.055359000
1	-2.722248000	-4.967519000	-2.390676000
1	-4.490587000	-4.995769000	1.552326000
6	1.054468000	2.348427000	-0.414895000
8	0.276363000	3.284617000	-1.144059000
8	2.206484000	2.245915000	-0.813209000
1	0.868120000	3.617144000	-1.851821000
8	-1.885514000	1.950206000	-2.161585000
8	-0.258370000	0.465815000	-2.578730000
6	-1.497143000	0.691770000	-2.504158000
8	-2.417209000	-0.130493000	-2.718378000
6	1.867301000	-2.190444000	-3.473278000
6	2.571445000	-0.977157000	-3.650032000
6	2.532758000	-3.403055000	-3.748759000
6	3.892844000	-0.980468000	-4.092441000
6	3.858996000	-3.387316000	-4.189230000
6	4.551359000	-2.185599000	-4.367394000
1	2.061183000	-0.039421000	-3.419789000
1	2.002187000	-4.340163000	-3.600804000
1	4.416247000	-0.028755000	-4.218676000
1	4.357859000	-4.338193000	-4.397052000
1	5.588044000	-2.186577000	-4.711895000
1	-1.086951000	2.442750000	-1.859178000

^{5}TS2$_{III}$, solvent = DMF:

26	-0.790006000	-0.987692000	19.329141000
8	-0.746956000	-6.590749000	15.505655000
7	-0.401986000	-3.053043000	19.306163000
7	1.017455000	-0.744151000	20.422412000
7	-2.269718000	-4.922904000	15.941863000
7	-0.305273000	-4.383065000	14.942513000
7	-1.380328000	0.899219000	20.067033000
7	-2.818016000	-1.438560000	19.040527000
6	-0.546169000	1.854281000	20.584029000
6	-1.251499000	-4.027274000	18.849505000
6	-3.845257000	-0.532929000	19.043837000
6	2.032531000	-1.659042000	20.485598000
6	-3.334617000	-2.648661000	18.651655000
6	-1.273869000	3.103174000	20.731286000
1	2.836805000	-3.606408000	20.193147000
6	-2.605520000	1.476091000	19.887809000
6	-2.551816000	2.867756000	20.301471000
6	-3.744755000	0.814912000	19.409781000
6	-0.533176000	-5.290662000	18.782019000
6	0.813153000	-3.640143000	19.548068000
1	-4.660122000	1.406816000	19.340595000
1	1.326620000	2.517984000	21.340321000
6	0.743283000	-5.047950000	19.209161000
6	-2.620716000	-3.857415000	18.540531000
6	1.939184000	-2.995529000	20.073881000
6	-4.754372000	-2.496778000	18.384294000
6	1.510180000	0.449892000	20.878077000
6	-3.089882000	-5.642269000	16.823582000
6	-3.356225000	-5.084174000	18.095907000
6	0.790571000	1.651138000	20.947431000
6	-5.071165000	-1.191146000	18.635080000
6	3.220226000	-1.027661000	21.028829000
6	2.896382000	0.281130000	21.272292000
6	-4.521626000	-7.533551000	17.357957000
6	-1.079366000	-5.401497000	15.458682000
6	-4.241168000	-5.745593000	18.959526000
6	-3.664790000	-6.874557000	16.474698000
6	-4.826614000	-6.961762000	18.597956000
1	-2.534985000	-3.899100000	15.770341000
1	-0.613251000	-3.391989000	15.175509000
1	-0.855771000	4.028017000	21.127193000
1	-3.390186000	3.562890000	20.270289000
1	-0.950514000	-6.233108000	18.438898000
1	1.566260000	-5.756000000	19.299688000
1	-5.413897000	-3.292759000	18.048396000
1	-6.042836000	-0.706115000	18.548805000
1	4.173650000	-1.527338000	21.197205000
1	3.530305000	1.066098000	21.683200000
1	-4.964029000	-8.491217000	17.071827000
1	-4.446857000	-5.308497000	19.939640000
1	-3.433005000	-7.300635000	15.498217000
1	-5.505924000	-7.468115000	19.287775000
6	0.302158000	-0.382640000	17.605137000
8	-0.835461000	1.066905000	16.680774000
8	1.344079000	-0.337328000	17.109089000
1	-0.241968000	1.228706000	15.932385000
8	-2.585870000	-0.333769000	15.768242000
8	-0.952680000	-1.853698000	15.567459000
6	-2.206317000	-1.570183000	15.581650000
8	-3.097535000	-2.467582000	15.410352000
6	0.932122000	-4.484300000	14.321069000
6	1.577125000	-3.270213000	13.982742000
6	1.576669000	-5.698695000	13.999086000
6	2.816716000	-3.274701000	13.347409000
6	2.819318000	-5.683120000	13.359340000
6	3.452762000	-4.481496000	13.027348000
1	1.075265000	-2.334002000	14.237379000

1	1.092477000	-6.634459000	14.266486000
1	3.293080000	-2.322142000	13.098668000
1	3.300846000	-6.635481000	13.118784000
1	4.425046000	-4.483327000	12.529128000
1	-1.669431000	0.377767000	16.216169000

^{5}IM2$_{III, solvent = DMF}$:

26	0.093530000	1.302327000	1.260511000
8	0.125044000	-4.117428000	-2.428364000
7	0.492730000	-0.784469000	1.333140000
7	1.992771000	1.610497000	2.167222000
7	-1.386011000	-2.468988000	-1.913464000
7	0.667946000	-1.891487000	-2.737243000
7	-0.419445000	3.220446000	1.997253000
7	-1.945097000	0.784120000	1.308245000
6	0.442038000	4.206141000	2.395030000
6	-0.372304000	-1.775079000	0.963209000
6	-2.970267000	1.690322000	1.373332000
6	3.016033000	0.700910000	2.155857000
6	-2.482350000	-0.423989000	0.942997000
6	-0.298685000	5.438026000	2.619848000
1	3.813702000	-1.250727000	1.884866000
6	-1.677913000	3.755771000	1.984125000
6	-1.613085000	5.158717000	2.366245000
6	-2.849939000	3.053065000	1.684545000
6	0.356081000	-3.034138000	0.856632000
6	1.733663000	-1.345755000	1.469277000
1	-3.779783000	3.626110000	1.704676000
1	2.386151000	4.914919000	2.891810000
6	1.658732000	-2.766593000	1.170797000
6	-1.759234000	-1.618580000	0.745466000
6	2.895748000	-0.660161000	1.837891000
6	-3.915851000	-0.277144000	0.774793000
6	2.528963000	2.826864000	2.492350000
6	-2.230532000	-3.251105000	-1.099102000
6	-2.489323000	-2.813788000	0.221212000
6	1.814482000	4.031934000	2.597225000
6	-4.218294000	1.028942000	1.054389000
6	4.255962000	1.362085000	2.511438000
6	3.953616000	2.682989000	2.719593000
6	-3.677396000	-5.169606000	-0.751749000
6	-0.174654000	-2.925733000	-2.369586000
6	-3.376506000	-3.550328000	1.018545000
6	-2.817154000	-4.433979000	-1.569412000
6	-3.970760000	-4.721226000	0.540300000
1	-1.580720000	-1.442429000	-1.937408000
1	0.389122000	-0.945129000	-2.448941000
1	0.138525000	6.381461000	2.945445000
1	-2.470094000	5.827397000	2.440905000
1	-0.076412000	-3.989344000	0.571282000
1	2.497125000	-3.461385000	1.207007000
1	-4.597504000	-1.070994000	0.479444000
1	-5.195576000	1.510216000	1.036324000
1	5.226373000	0.873018000	2.590683000
1	4.627594000	3.491314000	3.001709000
1	-4.129864000	-6.089851000	-1.129468000
1	-3.578169000	-3.208990000	2.036543000
1	-2.593671000	-4.762454000	-2.584758000
1	-4.651251000	-5.288621000	1.179511000
6	0.770208000	1.395035000	-0.738414000
8	0.183448000	4.236298000	-1.605414000
8	1.912281000	1.600570000	-1.113635000
1	0.978972000	3.696995000	-1.497379000
8	-1.723731000	2.289053000	-1.692826000
8	-0.035049000	0.787291000	-1.791345000
6	-1.399006000	1.103185000	-1.887578000
8	-2.124993000	0.144146000	-2.223036000
6	1.962394000	-2.000424000	-3.253746000
6	2.777208000	-0.848290000	-3.218731000
6	2.482201000	-3.183522000	-3.816763000
6	4.071275000	-0.884054000	-3.737341000
6	3.782672000	-3.200435000	-4.327808000
6	4.588636000	-2.058518000	-4.296548000
1	2.394262000	0.067023000	-2.759333000
1	1.864617000	-4.077489000	-3.834156000
1	4.685830000	0.019551000	-3.695975000
1	4.168520000	-4.128207000	-4.759455000
1	5.604322000	-2.083369000	-4.698143000
1	-0.522432000	3.544105000	-1.628133000
